# Customized Nanostructured Ceramics via Microphase Separation 3D Printing

**DOI:** 10.1002/advs.202304734

**Published:** 2023-09-26

**Authors:** Valentin A. Bobrin, Haira G. Hackbarth, Yin Yao, Nicholas M. Bedford, Jin Zhang, Nathaniel Corrigan, Cyrille Boyer

**Affiliations:** ^1^ Cluster for Advanced Macromolecular Design School of Chemical Engineering University of New South Wales Sydney NSW 2052 Australia; ^2^ School of Chemical Engineering University of New South Wales Sydney NSW 2052 Australia; ^3^ Electron Microscope Unit Mark Wainwright Analytical Centre University of New South Wales Sydney NSW 2052 Australia; ^4^ School of Mechanical and Manufacturing Engineering University of New South Wales Sydney NSW 2052 Australia; ^5^ Australian Centre for Nanomedicine School of Chemical Engineering University of New South Wales Sydney NSW 2052 Australia

**Keywords:** 3D printing, nanostructured ceramics, photoreversible addition‐fragmentation chain transfer (RAFT), polymerization induced microphase separation, self‐assembly of block copolymers

## Abstract

To date, the restricted capability to fabricate ceramics with independently tailored nano‐ and macroscopic features has hindered their implementation in a wide range of crucial technological areas, including aeronautics, defense, and microelectronics. In this study, a novel approach that combines self‐ and digital assembly to create polymer‐derived ceramics with highly controlled structures spanning from the nano‐ to macroscale is introduced. Polymerization‐induced microphase separation of a resin during digital light processing generates materials with nanoscale morphologies, with the distinct phases consisting of either a preceramic precursor or a sacrificial polymer. By precisely controlling the molecular weight of the sacrificial polymer, the domain size of the resulting material phases can be finely tuned. Pyrolysis of the printed objects yields ceramics with complex macroscale geometries and nanoscale porosity, which display excellent thermal and oxidation resistance, and morphology‐dependent thermal conduction properties. This method offers a valuable technological platform for the simplified fabrication of nanostructured ceramics with complex shapes.

## Graphical abstract

This work demonstrates a novel process that combines self‐assembly and additive manufacturing technique to create polymer‐derived silicon oxycarbide ceramics with independently tailored nano‐ and macroscopic features. This enables the fabrication of ceramics with excellent thermal and oxidation resistance, as well as tunable thermal conductivity. Such progress holds significant potential across diverse industries, including aeronautics, defense, microelectronics, and more.

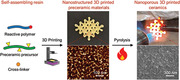

## 1 Introduction

Polymer‐derived ceramics are an important class of materials and have been widely used in electronics, energy, and extreme environment applications due to their impressive properties, including high thermal, and chemical stability.^[^ ^1^ ^]^ These ceramics are synthesized through thermal treatment of preceramic polymers, which provides advantages in terms of processability and shaping in comparison with conventional methods for ceramic preparation.^[^ ^2^ ^]^ Polymer‐derived ceramics can broadly be categorized into two classes based on their physical structure: conventional bulk ceramics and nanostructured ceramics.^[^ ^1a^ ^]^ The primary difference between these two classes of ceramic products is the presence of nanoscale structure, which can significantly enhance the material properties or confer new ones. Indeed, it has been shown that nanoscale features, e.g., nanoscale pores and voids, greatly enhances high temperature thermal insulation, crack inhibition, toughness, and energy absorption properties of nanostructured ceramics compared to their bulk counterparts.^[^ ^3^ ^]^ Therefore, substantial effort has been devoted to the development of robust and cost‐effective methods to fabricate nanoscale polymer‐derived ceramic structures.

Nanostructured polymer‐derived ceramics are traditionally obtained by thermal treatment of ordered preceramic templates. Conventional synthetic approaches for controlled fabrication of nanostructured preceramics rely on i) soft/hard templating,^[^ ^4^ ^]^ ii) self‐assembly of organic–inorganic block copolymers^[^ ^5^ ^]^ or co‐assembly of inorganic precursors with organic block copolymers,^[^ ^6^ ^]^ or iii) organic–inorganic hybrid polymerization‐induced microphase separation (PIMS).^[^ ^7^ ^]^ In the first approach, a preceramic precursor co‐assembles with soft organic materials or is filled into a preordered hard template followed by ceramization and selective template removal. The second and third approaches involve the generation of microphase separated structures of silicon‐containing block copolymers or inorganic precursors with organic block copolymers, which are then pyrolyzed to convert them into nanostructured ceramics. Although all three approaches produce ceramics with highly controlled nanoscale features, they are generally restricted to the fabrication of nanostructured ceramics with predominantly simple geometries, such as flat prisms. The demand for an effective fabrication technique capable of producing ceramics with intricate nano‐ and macroscopic structures has been a significant barrier to their broader adoption in critical technologies, such as microelectromechanical systems, filtration components, and customized biomedical implants.^[^ ^1^, ^8^ ^]^

With the progression of 3D printing technologies in recent years, particularly photoinduced 3D printing methods, the capability to produce a wide range of materials with complex geometries has considerably improved.^[^ ^9^ ^]^ More specifically, in the context of ceramics, recent advancements in this direction have enabled 3D printing of bulk silicon oxycarbide microlattice and honeycomb cellular materials by stereolithography,^[^ ^10^ ^]^ ultra‐lightweight 3D octet trusses and lattices produced by the combination of two‐photon lithography and atomic layer deposition,^[^ ^11^ ^]^ as well as direct writing of self‐assembling preceramic polymer‐triblock copolymer inks.^[^ ^3a^ ^]^ These approaches represent distinct technological pathways, each with advantages and shortcomings, toward the digital fabrication of ceramics. Self‐propagating photopolymer waveguide technology^[^ ^10^, ^12^ ^]^ and two‐photon lithography^[^ ^11^ ^]^ have successfully produced ceramic materials with high geometric complexity and controlled structures at sub‐micrometer scale, however, these ceramics lack controlled nanoscale features. Furthermore, these processes are driven by precisely engineered and specialized hardware and require specific monomers, thereby limiting their widespread use. While direct ink writing of preceramic polymers with organic block copolymers can lead to nanoscale morphologies as previously demonstrated by Lewis and co‐workers in a seminal paper,^[^ ^3a^ ^]^ this technique suffers from the typical geometrical limitations of extrusion‐based technologies.^[^ ^13^ ^]^ Therefore, the advancement of nanostructured polymer‐derived ceramics largely depends on the development of robust and versatile methods that enable precise control over the nano‐ and macrostructure of printed objects via inexpensive and unmodified equipment.

**Figure 1:**
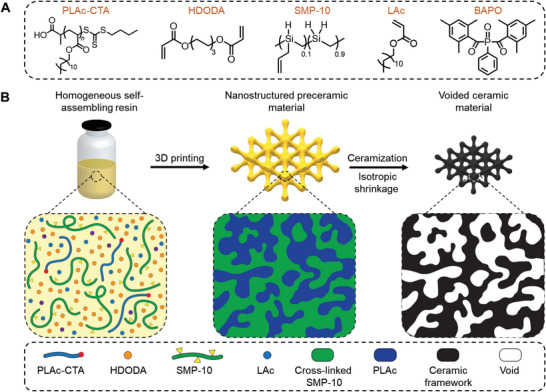
Fabrication route to 3D printed nanostructured ceramic materials. A) Chemical structures of resin components; B) A homogeneous self‐assembling resin composed of a macroCTA, photoinitiator, reactive preceramic polymer, and multifunctional monomers is used to fabricate complex‐shaped preceramic materials with well‐defined nanostructured domains of macroCTA and cross‐linked preceramic phases. These preceramic materials are then pyrolyzed to form nanostructured ceramics.

In this study, we report on a simple digital light‐processing 3D printing platform to rapidly fabricate customized nanostructured polymer‐derived ceramics (**Figure** **1**). The proposed method uses a photocurable self‐assembling preceramic resin composed of a reactive polymer, i.e., a macromolecular chain transfer agent (macroCTA), and a mixture of preceramic polymer and multifunctional monomers (Figure 1A). Upon 3D printing, photopolymerization of resin components results in the in situ chain extension of macroCTA to form block copolymers, which subsequently microphase separate to generate materials with nanoscale morphologies that are eventually arrested by cross‐linking (Figure 1B). Depending on the macroCTA chain length, 3D printed preceramic materials are shown to have precisely tunable internal nanostructures. Upon pyrolysis, the resulting nanostructured ceramic materials consist mostly of silicon oxycarbide (SiO*_x_* C*_y_*) and demonstrate tunable surface area and pore structure pre‐determined by the initial morphology of the preceramic materials, in addition to excellent temperature and oxidation resistance. We envision that the outcomes of this work will increase the scope and precision to which 3D printed ceramic materials may be fabricated and create pathways to their more advanced applications in the aerospace, defense, and biomedical sectors.

## 2 Results and Discussion

### 2.1 Development of Preceramic PIMS Resins

Nanostructured preceramic structures were fabricated using the PIMS 3D printing method.^[^ ^14^ ^]^ This process enables a wide range of 3D printed nanostructured polymeric materials^[^ ^15^ ^]^ to be produced by combining the accurate geometric control offered by modern digital light processing (DLP)^[^ ^16^ ^]^ with the precise control over nanostructuration traditionally achieved by PIMS processes.^[^ ^17^ ^]^ For successful 3D printing of nanostructured preceramic materials, the resin components should i) initially form a homogeneous solution, but undergo microphase separation upon photopolymerization, ii) polymerize quickly under open‐to‐air conditions, and iii) lead to a mechanically stable high‐resolution 3D structure. Considering these conditions, resin components were screened, and their compositions optimized accordingly. A commercially available allyl‐containing polycarbosilane, SMP‐10 (the ratio of allyl‐SiC_2_H/SiC_2_H_2_ = 1/9 per a polymer chain), was selected as the preceramic precursor. SMP‐10 was selected due to its ability to generate silicon carbide and silicon oxycarbide ceramic phases with high yield upon thermal treatment,^[^ ^1a^ ^]^ and also to copolymerize with acrylate monomers to form polymer network materials.^[^ ^8a^ ^]^ A difunctional acrylate, 1,6‐hexanediol diacrylate (HDODA), was used as a cross‐linker to copolymerize with the preceramic polymer in the presence of macroCTA to achieve a continuous polymer network with sufficient strength to fabricate mechanically stable 3D printed objects, as well as to provide additional cross‐linking points to increase dimensional stability of the preceramic phase upon pyrolysis.^[^ ^18^ ^]^ As a pyrolytic sacrificial block, a poly(lauryl acrylate) macroCTA (PLAc‐CTA) was selected due to its solubility in the preceramic precursor. PLAc‐CTA was synthesized by reversible addition‐fragmentation chain transfer (RAFT) polymerization^[^ ^19^ ^]^ of lauryl acrylate using 2‐(*n*‐butylthiocarbonothioylthio) propanoic acid as RAFT agent and 2,2′‐azobisisobutyronitrile as thermal initiator at 60 °C. The molar mass of PLAc‐CTAs was varied, as this parameter can enable the nanoscale morphology of 3D printed materials to be tuned.^[^ ^15a,c^ ^]^ Specifically, PLAc‐CTAs with five different number‐average molecular weights (*M*_n_) in the range of 6.9–69.2 kg mol^−1^ and low dispersities (*Ɖ* ≤ 1.3) were synthesized (Figure S1, Supporting Information). The degree of polymerization (*X*_n_) of the five PLAc‐CTAs as determined by proton nuclear magnetic resonance (^1^H NMR) spectroscopy were 28, 69, 103, 137, and 287 (Figure S2, Supporting Information). Further details on the PLAc‐CTA synthesis and characterization can be found in Table S1 (Supporting Information). Besides these core components, the resins also contained a small amount of lauryl acrylate (9 wt.%) to aid in solubilization of PLAc‐CTA in the mixture of SMP‐10, HDODA, and phenylbis(2,4,6‐trimethylbenzoyl)phosphine oxide (BAPO) (Figure S3, Supporting Information), a Norrish Type I radical photoinitiator commonly used for vat photopolymerization‐based 3D printing techniques.^[^ ^20^ ^]^ Photocurable preceramic resins were formulated upon mixing SMP‐10, HDODA, LAc, PLAc‐CTA, and BAPO in predetermined weight ratios. It was found that PLAc‐CTAs with *X*_n_ = 28, 69, 103, and 137 formed a homogeneous and an optically transparent solution at 14.9 wt.% macroCTA loading and SMP‐10/HDODA = 1/1 wt. ratio, indicating full solubilization of resin components (Figure S4, Supporting Information). However, the resins formulated with larger PLAc‐CTA chain length (*X*_n_ = 287) appeared as a turbid solution, indicating that PLAc_287_‐CTA was not completely soluble in the mixture of SMP‐10/HDODA = 1/1 w/w, and therefore this formulation was not used further (Figure S4, Supporting Information). In addition, the resins formulated with higher loading of PLAc‐CTA (20 wt.%) in the mixture of SMP‐10/HDODA = 1/1 w/w also resulted in the formation of a turbid solution (Figure S5, Supporting Information). Thus, for further study, a typical resin was formulated by dissolving 14.9 wt.% of PLAc‐CTA with varied *X*_n_ = 28, 69, 103, and 137 in a mixture of SMP‐10/HDODA = 1/1 w/w with 1.8 wt.% BAPO.

As a short inhibition period and rapid gelation of resins underpin successful RAFT‐mediated 3D printing,^[^ ^21^ ^]^ the photopolymerization kinetics of each preceramic resin was investigated in open‐to‐air conditions under 2.06 mW cm^−2^ violet light irradiation (*λ*_max_ = 405 nm) (Table S2, Supporting Information). As the absorption peak at 1630 cm^−1^ assigned to Si─C─C═C allylic overtone of SMP‐10 significantly overlaps with C═C stretching overtone of HDODA and LAc at 1600–1650 cm^−1^, the overall double bond conversion was monitored by following the decrease in the absorption peak at 1600–1650 cm^−1^ upon light irradiation of each resin (Figure S6, Supporting Information). Regardless of PLAc‐CTA *X*_n_, all resin formulations exhibited rapid gelation without a noticeable inhibition period, reaching ≈65% double bond conversion within 50 s (Figure S7, Supporting Information). This fast rate of polymerization and lack of inhibition period can be attributed to the Trommsdorff–Norrish effect^[^ ^22^ ^]^ in conjunction with the large radical influx produced from the photolytic decomposition of BAPO facilitating oxygen tolerance of our system via a polymerizing‐through mechanism.^[^ ^23^ ^]^ The double bond conversion leveled off at ≈80% after 180 s, presumably due to the formation of highly cross‐linked polymer network, thus inhibiting the efficient chain and monomer diffusion between an active radical and a dormant RAFT‐capped species required for the RAFT process.^[^ ^24^ ^]^ Overall, the kinetics results demonstrated the suitability of PIMS preceramic resins for photoinduced 3D printing.

### 2.2 Morphology Control in 3D Printed Preceramic Materials

**Figure 2:**
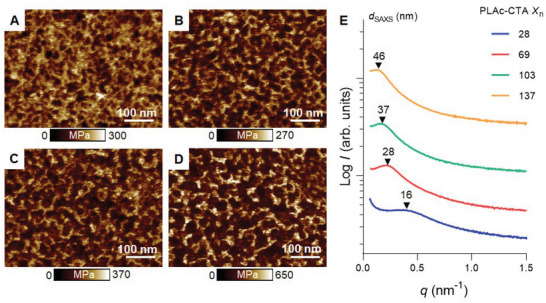
Characterization of 3D printed PIMS preceramic materials. Nanoscale morphology of preceramic materials was determined by A–D) AFM. PLAc‐CTA (15 wt.%) with various degree of polymerization (*X*_n_) of PLAc‐CTA, *X*_n_ = A) 28, B) 69, C) 103, and D) 137. E) SAXS profiles and corresponding domain spacing (*d*_SAXS_) values of preceramic materials 3D printed using 15 wt.% PLAc‐CTA with *X*_n_ = 28, 69, 103, and 137. SAXS profiles were shifted vertically for clarity.

To investigate the phase separation behavior of resins, we prepared four formulations with fixed chemical composition (Table S2, Supporting Information), but varied PLAc‐CTA *X*_n_, and applied them to 3D print preceramic objects in a prism shape using a commercial DLP 3D printer (Anycubic Photon Mono SE, *λ*_max_ = 405 nm, *I*_0_ = 2 mW cm^−2^). The layer slicing thickness and cure time were set to 100 µm and 60 s/layer, respectively. After 3D printing and post‐cure treatment under violet light (*λ*_max_  =  405 nm) for 40 min, the conversions of allyl bonds of SMP‐10 and vinyl bonds of HDODA and LAc were determined by Fourier transform near‐infrared spectroscopy (FTNIR) (Figures S8 and S9, Supporting Information). For all resin formulations, the resulting allyl bond conversion was in the range of 55–67%, while the conversion of vinyl bonds reached 97–99% (Table S3, Supporting Information), which is in agreement with the more active nature of acrylate groups compared to the allyl groups.^[^ ^25^ ^]^ All 3D printed preceramic materials were well‐defined, mechanically robust objects, however, visual transparency of objects decreased upon increasing PLAc‐CTA *X*_n_ from 28 to 137 (Figure S10, Supporting Information), which was attributed to changes in the characteristic length scale of microphase‐separated structures. To better understand the microphase separation behavior of resins and the effect of varying PLAc‐CTA *X*_n_ on the nanostructure of the printed preceramic objects, the materials were examined by atomic force microscopy (AFM). For all materials, the co‐existence of two distinct phases with different modulus was observed, confirming the nanostructuration of preceramic materials (**Figure** **2**A–D). For the preceramic material 3D printed with PLAc_28_‐CTA, we observed the generation of discrete globular PLAc domains (dark brown domains) distributed in the continuous *net*‐P(SMP‐10‐*stat*‐HDODA‐*stat*‐LAc) network (light brown domains) (Figure 2A). Increasing the *X*_n_ of the PLAc block resulted in elongation and increased continuity of PLAc domains, ultimately leading to the formation of bicontinuous morphologies when the *X*_n_ reached 137 (Figure 2B–D). Such morphological transition with increasing PLAc‐CTA *X*_n_ is a result of increasing block copolymer segregation strength, (*χ*_eff_·*N*, where *χ*_eff_ is the interaction parameter and *N* is the degree of polymerization) at larger *N*, which is in alignment with previous PIMS systems reported by our group^[^ ^15a^ ^]^ and other groups.^[^ ^26^ ^]^ Furthermore, close inspection of the AFM images revealed that the PLAc domain width monotonically increased from 9 to 20 nm with increasing *X*_n_ of the PLAc block from 28 to 137 (Figure S11A, Supporting Information), which is in agreement with previous findings for other systems.^[^ ^15^, ^26^ ^]^

As AFM only provides information on the nanostructure on the surface of 3D printed materials, the presence of internal nanoscale structures was confirmed by small‐angle X‐ray scattering (SAXS) analysis (Figure 2E). For all 3D printed preceramic materials, the presence of a single broad scattering peak without higher‐order peaks was observed, indicating the formation of disordered microphase‐separated morphology typically observed in PIMS systems.^[^ ^17^, ^26^ ^]^ Upon increasing the *X*_n_ of PLAc‐CTA from 28 to 137, a scattering peak maximum position shifted from 0.39 to 0.14 nm^−1^, indicating the increase in domain spacing (*d*_SAXS_) from 16 to 46 nm. This observation is consistent with previous reports^[^ ^15^, ^26^ ^]^ and reflects the increase in the average block copolymer size when using larger *X*_n_ macroCTAs, due to the lower number of polymeric chains containing RAFT groups at fixed mass fraction of macroCTA. Interestingly, the domain spacing scaling exponent (*δ* = 2/3) was found to be slightly larger than the scaling exponent reported for other PIMS systems (*δ* ≈3/5)^[^ ^15^, ^26^ ^]^ (Figure S11B, Supporting Information). This suggests that in our system, polymer chains are more stretched compared to those in previously reported PIMS systems. Such stretched conformation is typically associated with the strong segregation limit (*δ* = 2/3),^[^ ^27^ ^]^ which can be attributed to the increased block copolymer segregation strength in our system compared to other PIMS systems. As a result, the polymer chains adopt a perturbated and more stretched configuration, leading to the observed relationship of *d*_SAXS_ ≈*N* ^2/3^. Another potential factor contributing to the observed scaling exponent for domain spacings could be the co‐existence of PLAc‐*b*‐P(LAc‐*stat*‐HDODA) and unreacted SMP‐10 species, which may selectively swell PLAc and *net*‐P(SMP‐10‐*stat*‐HDODA‐*stat*‐LAc) nanodomains generated during PIMS (Figure S12, Supporting Information). The fitting of SAXS spectra to the Teubner–Strey (T–S) model,^[^ ^28^ ^]^ which is commonly used for structural analysis of PIMS materials,^[^ ^15^, ^26^ ^]^ allowed us to extract the three following structural parameters: the domain spacing (*d*_TS_), the correlation length (*ξ*), and the amphiphilicity factor (*f*_a_), which reflect the periodic spacing between domains, the spatial coherence of the interfaces, and the segregation strength at the interfaces, respectively (Table S4 and Figure S13, Supporting Information). In addition, the ratio of *ξ*/*d*_TS_ represents a measure of the domain size dispersity, with smaller values corresponding to domains with higher dispersity.^[^ ^29^ ^]^ The *f*_a_ and *ξ*/*d*_TS_ values for all samples were in the range from −0.43 to −0.58 and from 0.25 to 0.31, respectively, suggesting the formation of well‐structured domains with sharp interfaces and broad dispersity. For the lowest molecular weight macroCTA (PLAc_28_‐CTA), the *f*_a_ and *ξ*/*d*_TS_ values were −0.43 and 0.25, respectively. Comparatively, when using PLAc_69_‐CTA the *f*_a_ value decreased to −0.58, while the *ξ*/*d*_TS_ value increased to 0.31, indicating the generation of domains with sharper interfaces and low dispersity. This is likely a result of increased segregation strength between PLAc_69_‐CTA and *net*‐P(SMP‐10‐*stat*‐HDODA‐*stat*‐LAc) blocks. Further increasing the PLAc‐CTA *X*_n_ to 103 and 137 resulted in the *f*_a_ values of −0.53 and −0.48, and *ξ*/*d*_TS_ values of 0.29 and 0.27, respectively, demonstrating less well‐defined domain interfaces and higher domain dispersity, which can be attributed to the lower chain mobility due to higher viscosity and higher molecular weight of macroCTAs.

Importantly, preceramic materials 3D printed using either a non‐reactive PLAc_103_ (i.e., a polymer without CTA end group obtained via aminolysis of PLAc_103_‐CTA, Figures. S14 and S15, Supporting Information) or the mixture of non‐reactive PLAc_103_ and BTPA, were opaque (Figure S16A,D, Supporting Information). These results indicate the generation of materials with macrophase separated morphologies which were confirmed by AFM and SAXS (Figure S16B,C,E,F, Supporting Information). Moreover, as expected, preceramic materials 3D printed through terpolymerization of LAc, SMP‐10 and HDODA were transparent (Figure S17A,D, Supporting Information) and exhibited the generation of ill‐defined nanoscale morphologies as revealed by AFM and SAXS measurements (Figure S17B,C,E,F, Supporting Information). Altogether, these results demonstrate that the generation of 3D printed nanostructured preceramic materials follows a traditional PIMS process through the reactivation and further chain extension and microphase separation of macroCTA in the mixture of monomer/cross‐linker.

### 2.3 Preceramic‐to‐Ceramic Thermal Transformation

To evaluate the ceramic yield, SMP‐10, PLAc macroCTA, and 3D printed preceramic PIMS materials were characterized by thermogravimetric analysis (TGA, Figure S18, Supporting Information). SMP‐10 exhibited a single stage weight‐loss starting at ≈440°C with a ceramic yield of 89 wt.% at 900 °C, while PLAc‐CTA showed a significant weight loss of ≈95 wt.% from 250 to ≈420 °C and was completely decomposed by 600 °C. All 3D printed preceramic materials displayed nearly identical TGA profiles, exhibiting a significant weight loss of ≈55 wt.% in the temperature range of 250–550°C, associated with thermal decomposition of the PLAc‐CTA domains as well as the thermal reconstruction of *net*‐P(SMP‐10‐*stat*‐HDODA‐*stat*‐LAc) domains. These TGA profiles plateaued at temperatures above 550 °C, producing ceramics in 42–44 wt.% yield at 900°C, which were higher than the expected value (33 wt.%) presumably due to the formation of carbon phase in the ceramic structure as previously observed in other ceramics systems.^[^ ^10^, ^30^ ^]^ 3D printed preceramic materials were then subjected to a three‐stage pyrolysis protocol. First, the preceramic objects were heated under argon flow at 1°C min^−1^ to 160 °C and held at 160 °C for 2 h, followed by heating at 1°C min^−1^ to 230°C and holding at 230°C for 2 h. This process was used to thermally post‐cure the remaining allyl bonds of SMP‐10 to near‐quantitative conversion, which was confirmed by FTNIR (Figure S9 and Table S3, Supporting Information). Then, the thermally post‐cured preceramic materials were further heated to either 800 or 1200 °C using a constant heating rate of 1 °C min^−1^ to complete the pyrolysis process. Importantly, ceramic materials retained their original shape upon pyrolysis with no noticeable deformation (Figure S19, Supporting Information). Pyrolysis resulted in an isotropic linear shrinkage of the printed objects of ≈30 and ≈35% at 800 and 1200 °C, respectively, with ceramic yields of ≈44 wt.%, which was consistent with the TGA data (Figure S18 and Table S5, Supporting Information).

During the pyrolysis process, cross‐linked preceramic domains converted to a ceramic solid framework, while domains consisting of the macroCTA phase thermally decomposed to generate nanoscale voids. To monitor chemical changes associated with these two processes, attenuated total reflectance‐Fourier transform infrared spectroscopy analysis was performed (Figure S20, Supporting Information). The absorption peaks assigned to PLAc‐CTA and other organic components as well as some of the peaks of SMP‐10 precursor, e.g., Si─CH_3_ and Si─H, completely disappeared upon thermal treatment. For materials pyrolyzed at 800 or 1200 °C, two peaks assigned to Si─C stretching (≈ 800 cm^−1^) and Si─O transverse (≈1010 cm^−1^) phase vibration modes were observed (Figure S20B,C, Supporting Information), suggesting the formation of a silicon oxycarbide phase (SiOC).^[^ ^31^ ^]^ These results indicate the loss of all organic constituents during pyrolysis and completion of the ceramization process.

**Figure 3:**
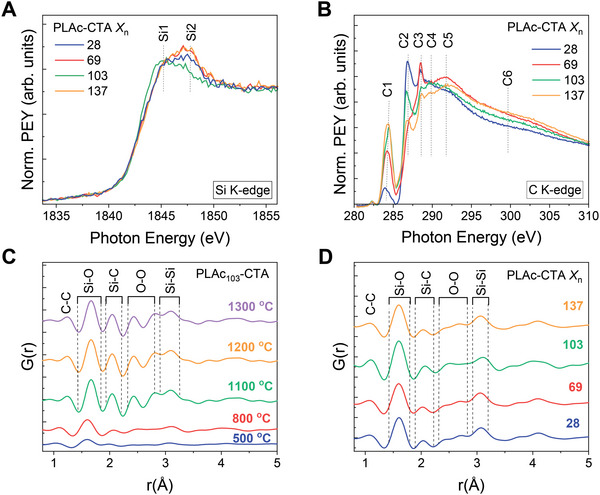
Characterization of preceramic‐to‐ceramic conversion by structural methods. NEXAFS spectra of the ceramic materials pyrolyzed at 800°C in the A) Si and B) C edges. Assignments of the peaks are given in Table S6 (Supporting Information). C) PDF profile of the ceramic materials prepared with PLAc_103_‐CTA upon in situ heating at different temperatures. D) PDF profiles of the ceramic materials 3D printed using PLAc‐CTA with *X*_n_ = 28, 69, 103, and 137 upon in situ heating at 800 °C.

To investigate the effect of PLAc‐CTA *X*_n_ on the local structure of 3D printed ceramics, near‐edge X‐ray absorption fine structure spectroscopy (NEXAFS) studies were performed on the Si and C K‐edges. For the ceramic materials pyrolyzed at 800°C, the Si K‐edge spectra exhibited broad absorption peaks near 1845 (peak Si1) and 1847 eV (peak Si2) (**Figure** **3**A), assigned to Si─C and Si─O bonding, respectively.^[^ ^32^ ^]^ The presence of overlapped peaks suggests a mixed environment of Si─C and Si─O bonding, which is likely attributed to the dominant SiOC phases. Interestingly, for the ceramic materials prepared with PLAc‐CTA *X*_n_ = 28, 69, and 137, the peak position was centered at 1847 eV, which suggests the presence of Si atoms in proximity to oxygen within Si‐O‐C units. Conversely, for ceramic materials 3D printed with PLAc_103_‐CTA, the pronounced peak at 1845 eV indicates a greater prevalence of Si‐C geometries.^[^ ^32^, ^33^ ^]^ C K‐edge NEXAFS provided further structural information on the ceramic materials from the perspective of C atoms (Figure 3B). The spectra exhibited features at energies between 284.3 to 299.7 eV (Table S6, Supporting Information), which were attributed to the transitions of C 1s orbitals to σ* occur from sp^3^ bonded C.^[^ ^32^, ^34^ ^]^ The peak fitting analysis of C K‐edge NEXAFS (Figure S21A–E, Supporting Information) revealed that the PLAc‐CTA *X*_n_ plays a significant role in the formation of sp^2^ bonded C atoms (284.3 eV, peak C1). In particular, the content of sp^2^‐hybridized C atoms increased as the PLAc‐CTA *X*_n_ increased from 28 to 137 (Figure S21F, Supporting Information), indicating the formation of C domains on the surface. The formation of sp^2^ C bonds is widely reported in Si‐based polymer‐derived ceramics and originates from C‐containing side groups from the polymer backbones.^[^ ^35^ ^]^ The increase in the content of sp^2^‐hybridized C phase with increasing PLAc‐CTA *X*_n_ is a result of increasing the average size of block copolymer of PLAc‐*b*‐(P(SMP‐10‐*stat*‐HDODA‐*stat*‐LAc)). Generally, longer polymer chains tend to result in higher amounts of sp^2^‐hybridized carbon compared to shorter chains due to multiple chain scission events, a higher probability of cross‐linking reactions during heat treatment, and diffusion limitations.^[^ ^36^ ^]^ Furthermore, HDODA and LAc units within *net*‐P(SMP‐10‐*stat*‐HDODA‐*stat*‐LAc) domains can serve as an additional source for the formation of sp^2^ C phase. Additional features observed in the C K‐edge spectra at 287.0 eV (peak C2), 288.4 eV (peak C3), and 289.7 eV (peak C4), were assigned to C─H, C─O, and C─C bonding environment, respectively. These features can be attributed to the presence of terminated C domains, which can exist in an amorphous sp^3^ configuration within C─H and C─C environments, as well as oxidized C sites located on the surface.^[^ ^32^, ^34^, ^37^ ^]^ Moreover, silicon oxycarbide ceramics are renowned for exhibiting multiple phases arising from SiC_4_ *‐_x_* O*_x_* (*x* = 0–4).^[^ ^32^, ^38^ ^]^ In the high energy spectra region, the relatively broad σ^*^ features ranging from 290 to 302 eV (peak C5 and C6) were attributed to sp^3^‐hybridized orbitals of C, which are in close contact with Si atoms.^[^ ^32^, ^34^, ^39^ ^]^

The application of in situ heating during total scattering measurements provides an opportunity to uncover any structural variations identified in NEXAFS. To evaluate the impact of the preceramic morphology and processing temperature on the atomic‐scale structure of the ceramic materials, high‐energy X‐ray diffraction (HE‐XRD) coupled with pair distribution function (PDF) analysis was conducted on materials pyrolyzed up to 1300°C. PDF analysis is ideal for probing materials with limited order, as both Bragg and diffuse features are considered in the Fourier transform into real space atomic pairs distances.^[^ ^40^ ^]^ The in situ HE‐XRD analysis revealed a lack of Bragg features in the long‐range, indicating that the materials were predominantly disordered at 1300 °C (Figure S22, Supporting Information). Typically, polymer‐derived ceramics obtained from polycarbosilanes crystallize into an ordered SiC phase at temperatures near 1200 °C.^[^ ^1^, ^41^ ^]^ However, our analysis suggests that the pyrolysis of 3D printed PIMS preceramic materials is likely to produce SiOC ceramics instead of a pure SiC phase, in line with prior observations.^[^ ^3a^ ^]^ SiOC ceramics are well known for their resistance to crystallization,^[^ ^38^, ^41^ ^]^ which explains the observed lack of long‐range order. To better comprehend the local structure information of the obtained ceramics, the HE‐XRD data were converted into their structure functions F(Q) (Figure S23, Supporting Information), and then Fourier transformed into atomic PDFs (Figure S24, Supporting Information). The atomic PDFs of the 3D printed ceramic materials exhibited prominent peaks at 1.6, 2.0, 2.6, and 3.05 Å, representing Si─O, Si─C, O─O, and Si─Si interatomic distances, respectively (Figure 3C,D) These features are likely to arise from the mixed Si─C and Si─O bond tetrahedrons of SiO*_x_* C_4_ *_−x_* units (*x* = 0–4). Additionally, the small contribution of C–C pairs was observed across all materials, as evidenced by the minor feature ≈1.3–1.4 Å. Interestingly, the PDF data under in situ heating at different temperatures exhibited notable differences, particularly in the Si─C, O─O and Si─Si contributions from 500 to 1300 °C (Figure 3C). These changes can be attributed to the generation of SiOC networks. Previous studies suggest that SiOC ceramics are formed by the redistribution reactions involving Si─O and Si─C bonds.^[^ ^38^ ^]^ However, PDF profiles of materials obtained at temperatures above 1100°C exhibit great similarities, suggesting comparable local structure. A comparison between the PDFs obtained at 800°C (Figure 3D) suggests slight differences in the local structure for the ceramic material prepared using PLAc_103_‐CTA. Specifically, this material displayed a higher weight of the Si‐C feature, which is consistent with the pronounced Si‐C absorption peak in the Si K‐edge NEXAFS (Figure 3A). Taken together, the NEXAFS and HE‐XRD data indicate that the ceramic materials exist in the form of amorphous SiOC phase along with C sp^2^ domains. These results were further supported by the energy‐dispersive X‐ray spectroscopy mapping and X‐ray photoelectron spectroscopy data (Figures S25–S28, Table S7, Supporting Information).

**Figure 4:**
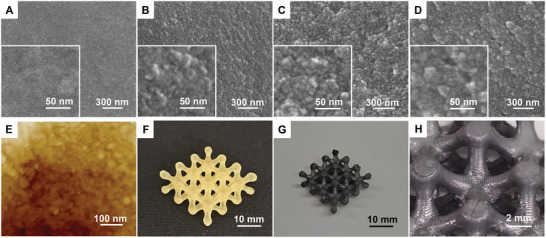
Ceramic PIMS materials. A–D) SEM images (cross‐section) of the pyrolyzed preceramic object (800 °C, under argon) prepared using PLAc‐CTA with *X*_n_ = A) 28, B) 69, C) 103, and D) 137. E) AFM height view of ceramic materials (800 °C, under argon) prepared using PLAc_137_‐CTA. F) 3D printed PIMS preceramic object prepared using PLAc_103_‐CTA. G) Pyrolyzed object (1200 °C, 1°C min^−1^ under argon). H) Close‐up view of (G).

To visualize the nanoscale morphology of ceramic objects, the cross‐sectional material surfaces were probed by scanning electron microscopy (SEM). All ceramic materials exhibited so‐called nanocoral morphologies^[^ ^3^, ^6^ ^]^ with the presence of nanoscale features (nanovoids) of progressively evolving size and definition in accordance with an increase in *X*_n_ of the PLAc block (**Figure** **4**A–D). AFM analysis performed on the surface of ceramic materials also confirmed the generation of nanocoral structure (Figure 4E). This morphology is formed upon pyrolytic removal of macroCTA domains yielding nanovoids embedded in the interconnected ceramic matrix. For the ceramic materials obtained upon pyrolysis at 1200 °C, SEM analysis of the cross‐section revealed that regardless of the PLA‐CTA *X*_n_, relatively similar nanoscale features were generated for all samples, which is a result of the sintering process at the higher pyrolysis temperature (Figure S29, Supporting Information). Further structural analysis of nanostructured ceramics was accomplished using SAXS. It was found that thermal decomposition and ceramization of PLAc and *net*‐P(SMP‐10‐*stat*‐HDODA‐*stat*‐LAc) domains, respectively, resulted in considerable changes in the SAXS profiles, with a significant decrease in the scattering peak sharpness and shift in the peak position to higher *q* (smaller *d*_SAXS_) compared to preceramic PIMS materials (Figure S30A, Supporting Information; Figure 2E). The *d*_SAXS_ values of ceramic samples calculated from the Lorentz‐corrected SAXS profiles were 9, 15, 17, and 14 nm for PLA‐CTA *X*_n_ = 28, 69, 103, and 137, respectively, which represents a significant *d*_SAXS_ reduction of 44–70% compared to the preceramic materials (Figure S30B, Supporting Information). This indicated that the extent of nanoscopic contraction is higher than the macroscopic one, suggesting the partial pore collapse within the ceramic framework due to high Laplace pressure, i.e., the pressure difference between the inside and outside of a curved liquid meniscus in a pore.^[^ ^42^ ^]^

To demonstrate the versatility of the preceramic PIMS 3D printing process, an object with complex macroscale architecture, a lattice structure with dimensions of 27 × 26 × 10 mm and a strut width of 1.8 mm, was designed and 3D printed using the preceramic resin containing PLAc_137_‐CTA. The 3D printed object replicated the original CAD model with high fidelity (Figure 4F; Figure S31, Supporting Information), with 3D printed dimensions of 27.6 × 27.1 × 9.7 mm and a strut width of 2.1 mm as measured using a digital caliper. Subsequently, the 3D printed preceramic lattice was pyrolyzed at 1200°C under argon with the heating rate of 1°C min^−1^ and hold at 1200°C for 60 min. Notably, the preceramic lattice object maintained its macroscopic architecture with isotropic shrinkage of ≈30% and no noticeable deformation (Figure 4G,H). Analogously, a preceramic lattice pyrolyzed at 800 °C also maintained its macroscopic shape (Figure S32, Supporting Information). These results demonstrate the ability to fabricate hierarchically structured ceramic objects using a commercial 3D printer, where macro‐ and nano‐architectures are controlled by digital‐ and self‐assembly afforded by DLP and PIMS preceramic resins, respectively.

### 2.4 Porosity Analysis by Gas Adsorption–Desorption Isotherms

**Figure 5:**
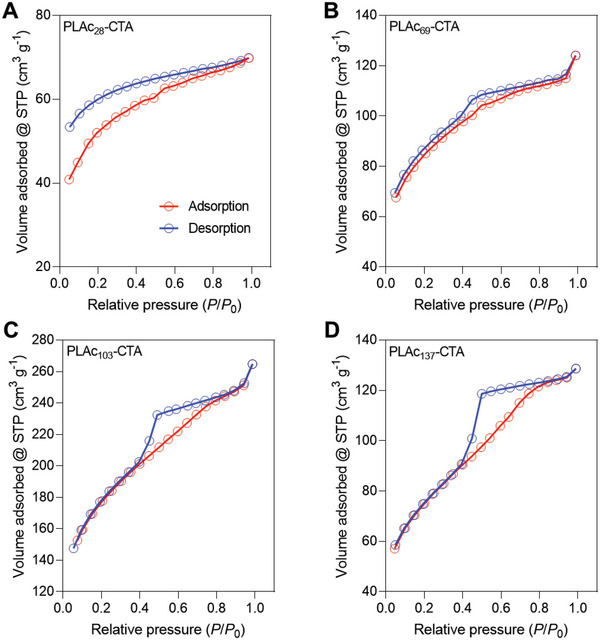
N_2_ at 77 K adsorption–desorption isotherms for ceramic materials prepared using PLAc‐CTA with various *X*_n_. *X*_n_ = A) 28, B) 69, C) 103, and D) 137. STP, standard temperature and pressure. Ceramic materials were obtained upon pyrolysis at 800 °C.

The porosity of 3D printed nanostructured ceramic materials pyrolyzed at 800 °C was probed by analyzing nitrogen adsorption‐desorption measurements. The shapes of the isotherms differed when the *X*_n_ of the PLAc‐CTA was varied, suggesting changes in pore structures (**Figure** **5**). The adsorption branch of the ceramic material 3D printed using PLAc_28_‐CTA resembled that of the type I isotherms characteristic of microporous materials.^[^ ^43^ ^]^ At low relative pressures (*P*/*P*_0_), the desorption and adsorption branches did not overlap with each other, resulting in the open type isotherm previously observed for some nanoporous PIMS materials^[^ ^26b^ ^]^ and materials with open slit‐like pores (Figure 5A).^[^ ^44^ ^]^ Such sorption behavior can be attributed to partial dissolution of nitrogen within the ceramic walls due to the high Laplace pressure and the capillary condensation within the micro‐ and mesopores.^[^ ^45^ ^]^ The ceramic materials prepared using PLAc‐CTAs with *X*_n_ = 69, 103, and 137 displayed a comparable isotherm shape similar to type IV with H2‐type hysteresis at *P*/*P*_0_ of 0.4 to ≈0.85,^[^ ^43^ ^]^ typically observed for disordered PIMS mesoporous materials (Figure 5B–D, Supporting Information).^[^ ^46^ ^]^ The hysteresis became more pronounced upon increasing *X*_n_ from 69 to 103 and 137, indicating a greater degree of capillary condensation, which may suggest the generation of more complex pore structures.^[^ ^47^ ^]^

Brunauer−Emmett−Teller (BET) surface areas and pore characteristics of 3D printed nanostructured ceramics as a function of *X*_n_ of the PLAc block are summarized in **Table** **1**. BET surface area (*S*_BET_) and pore volume (*V*_total_) of the ceramic materials increased from 164 to 589 m^2^ g^−1^ and from 0.11 to 0.41 cm^3^ g^−1^, respectively, upon increasing *X*_n_ of PLAc‐CTA from 28 to 103. Further increasing the PLAc‐CTA *X*_n_ from 103 to 137 resulted in lower *S*_BET_ and *V*_total_ of 260 m^2^ g^−1^ and 0.20 cm^3^ g^−1^, respectively, which can be attributed to the partial collapse or shrinkage of the pores due to lower degree of structural support provided by the surrounding matrix. Notably, for the 3D printed ceramic material prepared using PLAc_103_‐CTA, the determined *V*_total_ (0.41 cm^3^ g^−1^) was in close agreement with the expected pore volume (0.44 cm^3^ g^−1^). The other ceramic materials displayed *V*_total_ values less than expected, suggesting partial pore collapse or the generation of closed pores. Furthermore, apparent nitrogen sorption at low *P*/*P*_0_ indicated the formation of micropores (Figure 5). The contributions of micro‐ and mesopores to the overall surface area and pore volume were estimated using *t*‐plot method.^[^ ^47^, ^48^ ^]^ Upon increasing *X*_n_ of PLAc‐CTA from 28 to 137, the contribution of micropores to the total surface area (*S*_micro_/*S*_BET_) and pore volume (*V*_micro_/*V*_total_) decreased from 59 to 35% and from 45 to 20%, respectively, which can be attributed to the formation of nanovoids in the mesopore regime (below 50 nm) due to the use of sacrificial macroCTAs with larger *X*_n_. For materials pyrolyzed at 1200 °C, the pore structure could not be maintained when PLAc_24_‐CTA was used, however, ceramic materials produced using PLA‐CTAs with larger *X*_n_ demonstrated mesoporous structures, albeit at the cost of a reduced surface area and pore volume compared to the ceramics obtained at 800°C due to the ceramic framework densification (Figure S33, Supporting Information). The decrease in the porosity of ceramics resulted in the increase in the Vickers hardness from 2.3–4.4 to 6.8–11.6 GPa (Table S8 and Figure S34, Supporting Information). This observation agrees with the previous works regarding the dependency of the Vickers hardness of ceramics on porosity.^[^ ^49^ ^]^

**Table 1:** Surface areas and pore volumes of 3D printed ceramic materials prepared using PLAc‐CTA with various *X*_n_. Ceramic materials were obtained upon pyrolysis at 800 °C.

*X*_n_ of PLAc‐CTA	Surface area [m^2^ g^−1^]			Pore volume [cm^3^ g^−1^]		
	*S*_micro_ ^a)^	*S*_meso_	*S*_BET_	*V*_micro_ ^a)^	*V*_meso_	*V*_total_
28	97	67	164	0.05	0.06	0.11
69	136	142	278	0.07	0.12	0.19
103	296	293	589	0.16	0.25	0.41
137	91	169	260	0.04	0.16	0.20

^a)^ Determined by the *t*‐plot method. The mesopore surface area and pore volume were calculated by deducting the micropore area and volume from BET area and total pore volume, respectively, as proposed by Zhou et al.^[^ ^50^ ^]^ Note: the pore characteristics of the ceramic materials were determined based on open porosity.

The pore size distributions (PSDs) of the ceramic materials were evaluated using non‐local density functional theory (NLDFT), which provides an accurate method to characterize materials with complex pore structures,^[^ ^51^ ^]^ including nanoporous PIMS materials as shown in previous works.^[^ ^26a^ ^]^ For all *X*_n_ of PLAc‐CTA, broad PSDs were observed (Figure S35A, Supporting Information) presumably due to the generation of PLAc domains with the large dispersity (Table S4, Supporting Information), which upon decomposition gave rise to void channels with various sizes. Upon varying *X*_n_ from 28 to 103, the distribution shifted to a higher pore width area, indicating the formation of voids with larger size. A further increase of *X*_n_ to 137 did not result in a significant change in PSD. Similar observations were found for ceramic materials pyrolyzed at 1200 °C (Figure S35B, Supporting Information). Importantly, the ceramic materials prepared in the absence of PLAc‐CTA demonstrated different *S*_BET_, *V*_total_ and pore characteristics in comparison with PIMS ceramic materials with analogous chemical composition (Figures S36 and S37, Supporting Information). For instance, the ceramic materials prepared using either non‐reactive PLAc_103_ or the mixture of PLAc_103_ and BTPA exhibited largely meso‐ and macro‐porous structures and their resulting *S*_BET_ and *V*_total_ values were lower compared to the PIMS counterpart 3D printed using PLAc_103_‐CTA (Figure S36, Supporting Information). Analogously, the ceramic materials prepared by terpolymerization of SMP‐10, HDODA and LAc in the presence of BTPA displayed open‐type nitrogen sorption isotherms characteristic for slit‐like micropores (Figure S37, Supporting Information), which differ from their PIMS counterparts prepared using PLAc‐CTA with *X*_n_ = 69 and 103 (Figure 5B,C). The observation of pore populations with sizes between 2 and 4 nm for non‐PIMS materials (Figures S36B,E and S37B,E, Supporting Information) suggest that these pores possibly originated from the cross‐linked *net*‐P(SMP‐10‐*stat*‐HDODA‐*stat*‐LAc) domains rather than the sacrificial PLAc macroCTA domains. This can be attributed to the elimination of carbon and oxygen during ceramic formation.

### 2.5 Thermal Properties of 3D Printed PIMS Ceramics

**Figure 6:**
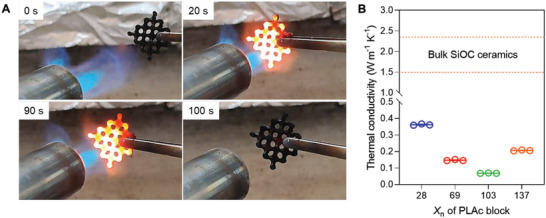
Thermal properties of PIMS ceramics. A) Optical images of 3D printed PIMS ceramic lattice demonstrating thermal shock resistance during heating in a butane gas flame (*T* > 1200 °C). The gas flame was turned off after 90 s. B) Thermal conductivity values at 400°C for PIMS SiOC ceramics versus PLAc‐CTA *X*_n_. Thermal conductivity of PIMS SiOC ceramics were lower than bulk polymer derived SiOC ceramics (1.5–2.4 W m^−1^ K^−1^).^[^ ^53^ ^]^ Data are shown as mean value (solid line) ± SD from three independent measurements (*n* = 3, shown as circles). Error bars fall within the size of the markers.

In comparison with bulk analogues, the 3D printed micro‐ and mesoporous ceramics prepared in this work have tunable surface area and void size. To explore whether these materials display distinct properties, the thermal and oxidative stability and thermal conduction properties of PIMS ceramic materials were studied. TGA of 3D printed PIMS ceramic materials recorded under nitrogen up to 900 °C (heating rate of 10°C min^−1^) revealed minor weight loss in the range of 1–4 wt.%, which is likely due to the degradation of low molar mass oligomers and the residual segregated carbon (Figure S38, Supporting Information). Under an air atmosphere, the weight loss was more pronounced, up to 10 wt.% of original mass for the material 3D printed using PLAc‐CTA *X*_n_ = 69, due to oxidative degradation of free carbon (Figure S38, Supporting Information).^[^ ^30^ ^]^ After two cycles of heating to 900 °C in air, the PIMS ceramic material exhibited no observable weight loss upon further heating (Figure S39, Supporting Information). Importantly, a PIMS 3D printed ceramic lattice demonstrated exceptional resistance to sudden and extreme changes in temperature, successfully withstanding exposure to a butane gas torch flame with a temperature exceeding 1200 °C (**Figure** **6**A; Figure S40 and Video S1, Supporting Information). This remarkable performance suggests that these materials hold great promise for potential applications in high‐temperature environments, such as hot gas filtration.^[^ ^52^ ^]^ Notably, the exposed ceramic material exhibited no dimensional changes or weight loss, and it retained its nanocoral morphology with the presence of nanoscale voids/pores (Table S9 and Figure S41, Supporting Information).

Finally, the thermal conductivities of PIMS ceramics as a function of temperature were measured and compared with bulk counterparts. For all samples, the obtained thermal conductivity values increased in the temperature range of 25–100 °C, and then gradually decreased until 400 °C (Figure S42, Supporting Information). Importantly, the obtained values were significantly lower than the thermal conductivity of bulk polymer derived SiOC ceramics (1.5–2.4 W m^−1^ K^−1^).^[^ ^53^ ^]^ This was attributed to the presence of nanovoids, which act as obstacles scattering the heat and resulting in its dissipation, along with the presence of trapped air within these nanovoids, which is a poor heat conductor.^[^ ^8c^ ^]^ The combined effect of these two factors resulted in the reduction of the overall thermal conductivity of the material. The comparison of thermal conductivity values obtained at 400 °C between PIMS ceramics revealed the effect of PLAc‐CTA chain length on the ceramic thermal conduction properties. As shown in Figure 6B, upon increasing PLAc‐CTA *X*_n_ from 28 to 103, the thermal conductivity values significantly decreased from 0.36 ± 0.004 to 0.07 ± 0.001 W m^−1^ K^−1^. Further increasing the PLAc‐CTA *X*_n_ to 137 resulted in the higher thermal conductivity value of 0.21 ± 0.003 W m^−1^ K^−1^. The thermal conduction property changes that occurred with changing *X*_n_ of PLAc‐CTA are related to the variations in pore structure of PIMS ceramics. As the *X*_n_ of PLAc block increased from 28 to 103, the porosity and the degree of pore interconnectivity of the resulting PIMS ceramic materials increased from 14 to 42% and from 25 to 93%, respectively (Table S10, Supporting Information). The higher porosity results in lower thermal conductivity due to the increased number of voids which act as air‐filled insulating pockets resulting in the overall reduction in the rate of heat transfer.^[^ ^54^ ^]^ In addition, the higher degree of pore interconnectivity can create more tortuous pathways for heat to travel, leading to a further reduction in thermal conductivity.^[^ ^55^ ^]^ Importantly, non‐PIMS ceramics prepared by terpolymerization of SMP‐10, HDODA and LAc in the presence of BTPA displayed higher thermal conductivity (0.34 ± 0.011 W m^−1^ K^−1^) than PIMS ceramic analogue prepared using PLAc_103_‐CTA (0.07 ± 0.001 W m^−1^ K^−1^). Altogether, these results demonstrated that the thermal conductivity of 3D printed nanoporous ceramics depends on their pore structure, which is pre‐defined by the preceramic morphology. This finding could be valuable for practical applications of these ceramics as thermal interface materials used as insulators to prevent heat from escaping a system, or as heat sinks to prevent sensitive components from overheating.

## 3 Conclusion

In summary, we have developed a DLP 3D printing platform that exploits photocurable self‐assembling hybrid resins to create ceramic materials with controlled nano‐ and macrostructures. Our method to fabricate nanostructured polymer‐derived ceramics offers several advantages over conventional approaches, including precise control over nano‐ and macroscopic features, compatibility with inexpensive and unmodified equipment, and the absence of structure‐directing block copolymer templates and organic solvents. These advantages significantly reduce the number of steps required for the preparation of nanostructured polymer‐derived ceramics with tailorable macroscopic geometries. In our method, photocurable resins containing preceramic precursors microphase separate and self‐assemble during DLP 3D printing to generate materials with disordered and kinetically trapped nanoscale morphologies. By carefully selecting the macroCTA *X*_n_, the domain spacing of nanostructured 3D printed materials was able to be finely tuned from 16 to 46 nm. Thermal treatment transforms the materials into SiOC‐based ceramics with disordered micro‐ and mesoporous structures, exhibiting excellent thermal and oxidative stability. The surface area and pore volume of these materials can be tailored between 164 to 589 m^2^ g^−1^ and 0.11 to 0.41 cm^3^ g^−1^, respectively. The thermal conductivity of PIMS ceramics is highly dependent on their pore structure, which is pre‐determined by the initial nanoscale morphology of the preceramic materials. The thermal conductivity value can be as low as 0.07 ± 0.001 W m^−1^ K^−1^, which is 21 times lower than the thermal conductivity of bulk silicon oxycarbide.

Notably, the approach developed in this work has the potential to be applied to other photoinduced 3D printing techniques, e.g., stereolithography, volumetric 3D printing, and two‐photon polymerization. This would expand the range of available macro‐ and microscopic ceramic geometries with controlled internal nanostructure. By providing a simplified pathway to nanostructured ceramics with designer shapes, our proposed method represents a significant advancement in the field of ceramic materials, opening new possibilities for diverse applications. For example, the resulting materials could be used to fabricate intricate thermal interface components, customized catalyst supports, porous ceramic fuel cells and porous burners for aerospace, energy, and engineering sectors, respectively.

## Conflict of Interest

The authors declare no conflict of interest.

## Supporting information

Supporting Information

Supplemental Video 1

## Data Availability

The data that support the findings of this study are available from the corresponding author upon reasonable request.
