# Supplementary material for: Customized Nanostructured Ceramics via Microphase Separation 3D Printing
Source: Adv Sci (Weinh). 2023 Sep 26;10(32):2304734. doi: 10.1002/advs.202304734 (PMC10646229; doi:10.1002/advs.202304734)
Supplement: Supplementary file 1 — Supporting Information [file ADVS-10-2304734-s002.pdf]

ADVANCED SCIENCE
Open Access

Supporting Information

for *Adv. Sci.*, DOI 10.1002/advs.202304734

Customized Nanostructured Ceramics via Microphase Separation 3D Printing

Valentin A. Bobrin, Haira G. Hackbarth, Yin Yao, Nicholas M. Bedford, Jin Zhang, Nathaniel Corrigan* and Cyrille Boyer**

Supporting Information

Customized Nanostructured Ceramics via Microphase Separation 3D Printing

Valentin A. Bobrin,[1] Haira G. Hackbarth,[2] Yin Yao,[3] Nicholas M. Bedford,[2] Jin Zhang[*,4], Nathaniel Corrigan[*,1], Cyrille Boyer[*,1,5]

[1]Cluster for Advanced Macromolecular Design, School of Chemical Engineering, University of New South Wales, Sydney, NSW 2052, Australia

[2]School of Chemical Engineering, University of New South Wales, Sydney, NSW 2052, Australia

[3]Electron Microscope Unit, Mark Wainwright Analytical Centre, University of New South Wales, Sydney, NSW 2052, Australia

[4]School of Mechanical and Manufacturing Engineering, University of New South Wales, Sydney, NSW 2052, Australia

[5]Australian Centre for Nanomedicine, School of Chemical Engineering, University of New South Wales, Sydney, NSW 2052, Australia

*Email: cboyer@unsw.edu.au; n.corrigan@unsw.edu.au; jin.zhang6@unsw.edu.au.

List of abbreviations

AFM	atomic force microscopy
ATR-FTIR	attenuated total reflectance-Fourier transform infrared spectroscopy
BET	Brunauer−Emmett−Teller
DLP	digital-light processing
FTNIR	Fourier transform near-infrared spectroscopy
HE-XRD	high-energy X-ray diffraction
MacroCTA	macromolecular chain transfer agent

NEXAFS	near-edge X-ray absorption fine structure spectroscopy
PDF	pair distribution function
PIMS	polymerization-induced microphase separation
RAFT	reversible addition-fragmentation chain transfer
SAXS	small-angle X-ray scattering
SEM	scanning electron microscopy
TGA	thermogravimetric analysis

Materials and Methods

Materials

Unless otherwise stated, all chemicals were used as received. The solvents were of either HPLC or AR grade; these included toluene (Chem-Supply, AR), acetonitrile (RCI Labscan Limited, RCI Premium) and tetrahydrofuran (THF, RCI Labscan Limited, HPLC). Aluminium oxide basic (Acros Organics, Brockmann I, 50–200 µm, 60A), 2-(*n*-butylthiocarbonothioylthio)propanoic acid (BTPA, Boron Molecular, >95%), (phenylphosphoryl)bis(mesitylmethanone) (BAPO, AmBeed, 99%), 2,2'-azobis(2-methylpropionitrile) solution (AIBN solution, Sigma-Aldrich, 0.2M in toluene), starPCS™ SMP-10 (Starfire® Systems, Inc.) and 1,6-hexanediol diacrylate (HDODA, Sigma-Aldrich, technical grade, 80%) were used as received. Lauryl acrylate (LAc, Sigma-Aldrich, technical grade, 90%) was passed through a basic aluminium oxide column to remove inhibitor prior to use.

Characterization methods

Nuclear magnetic resonance (NMR)

All NMR spectra were recorded on Bruker Avance III 400 MHz spectrometer using an external lock (toluene-d8 or chloroform-d).

Size exclusion chromatography (SEC)

Analysis of the molecular weight distributions of the polymers were determined using a Shimadzu modular system composed of an SIL-10AD auto-injector, a Polymer Laboratories 5.0 µm bead-size guard column (50 × 7.5 mm^2) followed by four linear PL (Styragel) columns (10^5, 10^4, 10^3 and 500 Å), and a UV detector. The eluent was THF at 40°C, run at a flow rate of 1.0 mL/min. The SEC was calibrated using narrow polystyrene (PSTY) standards with molecular weights of 200 – 10^6 g/mol.

Attenuated total reflectance-Fourier transform infrared (ATR-FTIR) spectroscopy

ATR-FTIR spectroscopy was performed to monitor photopolymerization kinetics using the Bruker Alpha FTIR spectrometer equipped with room temperature DTGS detectors. After taking a background reading of the empty plate, 20 µL of polymerization resin was pipetted onto the ATR crystal plate. An absorption

spectrum was then obtained by scanning the droplet from 400 to 4000 cm^{-1}. After an initial reading, the droplet was irradiated with a Thorlabs mounted LED with a collimation adapter (λ_{max} = 405 nm, I_0 = 2.06 mW cm^{-2}) and subsequently the IR absorption spectra were obtained at various times to determine the integral of the vinylic peak at t_x. Double bond conversions were calculated from the disappearance of peaks at 1600-1650 cm^{-1} (overlapped peaks of Si-C-C=C allyl overtone at 1630 cm^{-1} and C=C stretching overtone at 1600-1650 cm^{-1}) normalized to the C=O stretching peak at 1760 cm^{-1} as an internal standard using equation S1:

$$Conversion\ (\%) = 100\ \times \left(1 - \frac{int_x/std_x}{int_0/std_0}\right) \qquad \textbf{(Eq. S1)}$$

Where int_x is the integral of the 1600-1650cm^{-1} peak at x min of irradiation, std_x is the integral of the 1670-1800 cm^{-1} peak at x min of irradiation, int_0 is the initial integral of the 1600-1650 cm^{-1} peak before irradiation, and std_0 is the initial integral of the 1670-1800 cm^{-1} peak before irradiation. The conversion was monitored using 10 s intervals between 0 to 1 min, then every 30 s between 1 and 2 min, and every 1 min between 2 and 5 min. All FTIR measurements were performed in triplicate.

Fourier transform near-infrared (FTNIR) spectroscopy

FTNIR spectroscopy was performed using a Bruker Vertex 70 Fourier transform spectrometer. FTNIR spectroscopy was used to determine vinyl and allyl bonds conversions of solid samples by comparing the integral of the C-H vinylic stretching overtone at 6120-6220 cm^{-1} and the C-H allylic stretching overtone at 6080-6150 cm^{-1} between solid printed samples and a sample of uncured resin. Uncured resin (3 mL) was placed in a 1×1 cm quartz cuvette, and an absorption spectrum was obtained by scanning from 4000 to 8000 cm^{-1}. A solid 3D printed sample was measured using a digital caliper, placed in the cuvette and another absorption spectra was measured. The final conversion was calculated using Equation S2:

$$Conversion\ (\%) = 100\ \times \left(1 - \left(\frac{int_f}{int_0} \times \frac{t_{cuvette}}{t_{sample}}\right)\right) \qquad \textbf{(Eq. S2)}$$

Where int_f is the integral of the peak from either 6080-6150 or 6120-6220 cm^{-1} for the 3D printed sample, int_0 is the integral of the peak from either 6080-6150 or 6120-6220 cm^{-1} for the unpolymerized resin, t_{sample} is the thickness of the sample, and $t_{cuvette}$ is the path length of the cuvette. Integrals were calculated using OPUS software 7.5.

A similar protocol was utilized to monitor the conversions of allyl bonds of SMP-10 and vinyl bonds of HDODA and LAc of the resin (SMP-10 (36.9 wt%), HDODA (36.9 wt%), LAc (9.4 wt%), BAPO (1.8 wt%) and $PLAc_{103}$-CTA (14.9 wt%)). Specifically, 600 µL of the resin was placed in a quartz cuvette (2 mm path length) and an absorption spectrum was obtained by scanning the resin from 4000 to 8000 cm^{-1}. After an initial reading, the resin was irradiated with a Thorlabs mounted LED with a collimation adapter (λ_{max} = 405 nm, I_0 = 2.06 mW cm^{-2}) and subsequently the absorption spectra were obtained at various times to determine vinyl and allyl bonds conversions by comparing the integrals of the C-H vinylic stretching overtone at 6120-6220 cm^{-1} and the C-H allylic stretching overtone at 6080-6150 cm^{-1} at x min of irradiation and before irradiation.

Atomic force microscopy (AFM)

All AFM measurements were performed on the Bruker Dimension ICON SPM, with a Nanoscope V controller (software version 9.70). Mechanical property measurements were performed using peak force tapping mode on a top layer of printed object using the SCANASYST probe (from www.brukerafmprobes.com). The scan size was set to 1 µm and 300 nm. The scan rate was set at around 0.7 to 0.8Hz with a peakforce of approximately 500 pN. The feedback gain was adjusted accordingly to optimize tracking of the specimen surface. The resolution of the image was set to 512 pixels per line for 1 µm scan size and 256 samples/line for 300 nm scan size. For peakforce mechanical measurements, the tip was calibrated using the thermal tunning method. AFM images were analysed using the NanoScope Analysis software (version 1.7).

Small-angle X-ray scattering (SAXS)

SAXS experiments were performed on an Anton Paar SAXSPoint 2.0 system with a Cu K_α (λ = 0.154 nm) microfocus X-ray source (50 kV/1 mA) and Dectris Eiger 1M detector. Data were collected at room temperature, under vacuum for 5 min from a sample at a sample-to-detector distance of 0.575 m. Samples were 3D printed at the thickness of 2×100 µm layers. Data were reduced to 1D by radial averaging the 2D detector after converting pixel positions to $q = (4\pi/\lambda)\sin\theta$, where 2θ is the scattering angle). The domain spacing was calculated using equation S3:

$$d_{SAXS} = \frac{2\pi}{q} \quad \textbf{(Eq. S3)}$$

SAXS fitting using the Teubner-Strey (T-S) model

The position and the sharpness of SAXS peaks of microphase-separated 3D printed preceramic materials were fitted using the T-S model[1] in SasView software. According to T-S model (Equation S4)

$$I(q) = \frac{1}{a_2 + c_1 q^2 + c_2 q^4} + b \quad \textbf{(Eq. S4)}$$

Where $q = (4\pi/\lambda)sin\theta$, λ is the wavelength, 2θ is the scattering angle; b is background scattering; a_2, c_1, c_2 are fitting parameters used to calculate domain spacing (d_{TS}), correlation length (ξ) and the amphiphilicity factor (f_a) using equations S5-7 below:

$$d_{TS} = 2\pi \left[\frac{1}{2}\left(\frac{a_2}{c_2}\right)^{1/2} - \frac{1}{4}\frac{c_1}{c_2}\right]^{-1/2} \quad \textbf{(Eq. S5)}$$

$$\xi = \left[\frac{1}{2}\left(\frac{a_2}{c_2}\right)^{1/2} + \frac{1}{4}\frac{c_1}{c_2}\right]^{-1/2} \quad \textbf{(Eq. S6)}$$

$$f_a = \frac{c_1}{\sqrt{4a_2c_2}} \quad \textbf{(Eq. S7)}$$

Scanning electron microscopy (SEM)

SEM images were obtained using a field-emission NanoSEM 230 instrument with a 2 – 5 kV accelerating voltage and a secondary electron detector. EDX analysis was performed using the Nova NanoSEM 230, equipped with a silicon drift detector for EDX analysis (X-MAX Oxford Instruments), the acceleration voltage was set to around 10 kV with a spot size of around 5, the captured data were processed using the Aztec 6.0 software.

X-ray photoelectron spectroscopy (XPS)

X-ray photoelectron spectroscopy (XPS) measurements were performed on a K-Alpha X-ray Photoelectron spectrometer system (Thermo Fisher Scientific, U.K.) with a monochromatic Al Kα source (energy 1480 eV) at 120 W over 500 μm at a 90° angle, background vacuum pressure was set at 2×10^{-9} mbar.

Physisorption analysis

The surface area, pore volume and pore size distribution (PSD) of ceramic materials were determined from N_2 (77 K) adsorption-desorption isotherms using a NOVAtouch LX^2 gas sorption analyzer. Samples were loaded in type B long non-elutriating cell kit, 9 mm large bulb with dimple and degassed for 10 h at 350 °C before measurement. The surface area was calculated using a Brunauer-Emmett-Teller (BET) method in the linear range of P/P_0 = ~ 0.1–0.3. The total pore volume was measured using a single point absorption at P/P_0 of ~0.99. PSD was estimated using the non-local density functional theory (NLDFT), kernel: N_2@77 K, silica/zeolite, NLDFT adsorption model. The micropore volume and surface area were determined using the t-plot method.

Thermogravimetric analysis (TGA)

TGA of 3D printed preceramic and ceramic materials was performed at a rate of 10 °C min^{-1} under nitrogen as well as under air using a TA Instrument TGA Q5000 thermogravimetric analyzer.

Near-edge X-ray absorption fine structure spectroscopy (NEXAFS)

Near-edge X-Ray absorption fine structure (NEXAFS) measurements were conducted at the SXR beamline of the Australian synchrotron to probe the C K-edge (280-320 eV) and Si K-edge (1820-1870 eV). The powdered samples were pressed onto copper (Cu) tape for partial electron yield detection. All NEXAFS data reduction and processing was performed using the QANT program[2]. For Si K-edge, a set of 3 repeat scans were recorded and averaged for each sample. C K-edge measurements were double normalized against an in-line photodiode to account for carbon contamination in the X-ray optics. The C K-edge peaks in the NEXAFS spectra were decomposed into Gaussian peaks using the semi-automated peak fitting methods in QANT in Igor ProGUI[2b], to assign transitions. The C K-edge spectra were fitted with six main peaks around 284.3, 287.0, 288.4, 289.7, 291.7 and 299.7 eV.

In situ high-energy X-ray diffraction (HE-XRD)

In situ HE-XRD measurements were performed at the 11-ID-C beamline (Advanced Photon Source, Argonne National Laboratory) using a 105.7 keV irradiation (λ = 0.11730 Å). The heating of 3D-printed materials was conducted using two experiment configurations to account for two distinct heating ranges. For *in situ* heating to 500 and 800 ^{o}C, a capillary flow cell[3] was utilized. Prior to loading into quartz capillary sample holders, approximately 10 mg of 3D printed materials underwent grinding using a mortar and pestle. Quartz filter paper was positioned on both sides of the powders to confine them to the heating element region, ensuring a uniform heating gradient. The temperature during pyrolysis was monitored using a K-type thermocouple in contact with the powder sample within the capillary. Materials were heated at a rate of 20 °C/min and 100 °C/min for cooling under a constant argon flow. In-situ PDF measurements taken at 1100, 1200 and 1300 ^{o}C implement a TS1500 heating stage (Linkam Scientific)[4] modified with Kapton windows and sample holder configuration to account for possible sample expansion during pyrolysis. Printed materials examined in the TS1500 stage were pre-pyrolyzed in a bench-top alumina tube furnace, using a 1 °C/min ramp rate to 1000 ^{o}C and 1 h of dwell time under argon flow. The pre-pyrolyzed materials were then loaded into an alumina capillary with both sides covered with paper filter and placed into the TS1500 crucible with the axis of the capillary parallel to the incident X-rays. Materials were ramped to 1000 C using a ramp rate of 100 °C/min and then 20 °C/min for each of the 100 °C increments under a constant argon flow. Scattering was measured for both the empty quartz capillary and the alumina cup covered with paper filter at the temperatures investigated to account for their possible thermal expansion in the background subtraction procedure. A CeO_2 standard was used for calibration and to determine sample to detector distance for both configurations. The *in situ* HE-XRD patterns were background-corrected, transformed into reduced structure factors, and Fourier-transformed into their atomic pair distribution functions (PDFs) using the program PDFGetX3[5].

Measurement of specific heat capacity of ceramic materials

Specific heat capacity was measured according to ASTM standard E1269, which includes the running of three scans, baseline scan (empty pan run), sapphire standard scan, and sample scan using the digital scanning calorimetry technique (TA instruments, Q20). Specific heat capacity was calculated using equation S8:

$$Specific\ heat\ capacity = \frac{Signal\ difference\ (Sample - Baseline)}{Mass\ (sample) * heating\ rate * sensitivity} \qquad \textbf{(Eq. S8)}$$

where sensitivity was derived based on the sapphire and baseline scans using equation S9:

$$Sensitivity = \frac{Signal\ difference\ (Sapphire - Baseline)}{Mass\ (sapphire) * heating\ rate * theoretical\ Cp\ (sapphire)} \qquad \textbf{(Eq. S9)}$$

Thermal conductivity measurement

Thermal conductivity (κ) was calculated using equation S10:

$$\kappa\ =\ density\ \times specific\ heat\ capacity\ \times thermal\ diffusivity \quad \textbf{(Eq. S10)}$$

Thermal diffusivity was determined at T = 298, 323, 373, 473, 573 and 673 K under air conditions using laser flash apparatus (NETZSCH, LFA 467 HyperFlash). Samples were dried in a vacuum oven at 70 °C for 4 h prior to measuring their mass and volume. The average value of three measurements was taken at each temperature.

Estimation of the theoretical pore volume of ceramic materials

It was assumed that the pores (micro- and mesopores) mostly stem from the thermal decomposition of organic constituents, which include PLAc-CTA, HDODA, and LAc. We estimated the theoretical pore volume using the weight fraction of organic constituents in a feedstock resin using equation S11:

$$w_{org.const.}(\%) = w_{PLAc-CTA} +\ w_{HDODA} +\ w_{LAc} = 15 + 37 + 9 = 61\% \quad \textbf{(Eq. S11)}$$

In 1 g of 3D printed preceramic material, *net*-P(SMP-10-*stat*-HDODA-*stat*-LAc)-*b*-PLAc, the mass of organic constituents is

$$m_{org.const.} =\ m_{preceramic\ material}\ \times\ w_{org.const.} = 1\ g\ \times 0.61 = 0.61\ g \quad \textbf{(Eq. S12)}$$

Converting $m_{org.const.}$ to volume using equation S13 below:

$$V_{org.const.} = \frac{m_{org.const.}}{\rho_{org,const.}} = \frac{0.61\ g}{1\ g\ cm^{-3}} = 0.61\ cm^3 \quad \textbf{(Eq. S13)}$$

assuming the density of organic constituents is $\rho_{org,const.}$ = 1 g cm^{-3}.

The ceramic yield of *net*-P(SMP-10-*stat*-HDODA-*stat*-LAc)-*b*-PLAc is ~44 wt% according to TGA data (**Table S5**). Therefore, the mass of ceramics from 1 g of *net*-P(SMP-10-*stat*-HDODA-*stat*-LAc)-*b*-PLAc is

$$m_{ceramics} = 1\ g\ \times 44\% = 0.44\ g \quad \textbf{(Eq. S14)}$$

The theoretical pore volume can be estimated using equation S15:

$$V_{pore,theorectical} = V_{shrink}\%\ \times\ \frac{V_{org.const.}}{m_{ceramics}} = 32\%\ \times\ \frac{0.61\ cm^3}{0.44\ g} = 0.44\ cm^3\ g^{-1} \quad \textbf{(Eq. S15)}$$

where $V_{shrink}\%$ is the extent of the volumetric shrinkage of preceramic material during pyrolysis (**Table S5**). $V_{shrink}\%$ was averaged among all pyrolysis experiments and estimated to be 32%.

Vickers microhardness testing

Vickers hardness (HV) was determined by Vickers indentation (DuraScan-80, Struers) under the load of 1 kg and dwell time of 10 s.

Synthetic Procedures

RAFT polymerization of lauryl acrylate in toluene

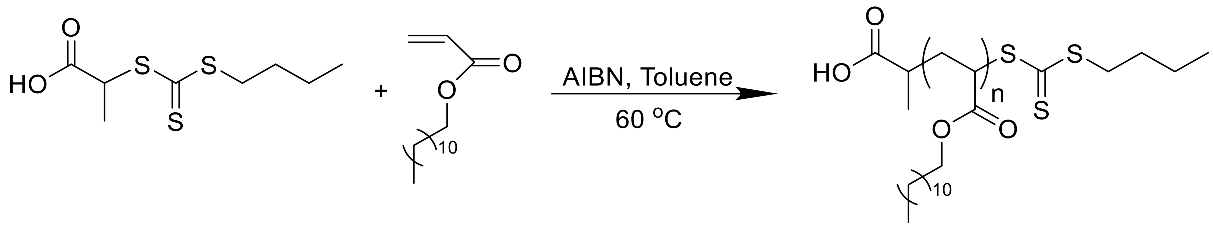

Fig. S1. Synthesis of PLAc$_n$-CTA using RAFT polymerization of lauryl acrylate.

Protocol for the synthesis of PLAc$_{103}$-CTA: lauryl acrylate (30 g, 0.125 mol), BTPA RAFT agent (0.266 g, 1.1×10^{-3} mol) and AIBN (0.2 M solution in toluene, 0.84 mL, 1.7×10^{-4} mol) were dissolved in toluene (60 mL). The mixture was deoxygenated by purging with nitrogen for 90 min, and then polymerized for 15 h at 60 °C. The reaction was stopped by cooling inside a freezer (-20 °C) for 30 min and exposing to air. The polymer solution was concentrated by rotary evaporation and the polymer was recovered by precipitation into large excess of acetonitrile, isolated by centrifugation, and then dried under vacuum using rotary evaporation to get a yellow viscous liquid. Using the same protocol, other PLAc-CTAs were synthesized. The characterizations for PLAc-CTAs are summarized in Table S1.

Table S1. Characterization of PLAc-CTAs synthesized by RAFT-mediated polymerization of lauryl acrylate in toluene.

MacroCTA	Monomer conversion (%)[a]	M_n (theory) (kg/mol)[b]	SEC (RI, THF)[c]		^{1}H NMR[d]		
			M_n (kg/mol)	$Đ$	X_n	M_n (kg/mol)	End-group fidelity, f (%)
PLAc$_{28}$-CTA	93	6.9	5.5	1.13	28	6.9	94
PLAc$_{69}$-CTA	92	16.8	11.5	1.19	69	16.8	92
PLAc$_{103}$-CTA	92	25.0	14.1	1.23	103	25.0	92
PLAc$_{137}$-CTA	91	33.1	19.5	1.20	137	33.1	90

$PLAc_{287}$-CTA	82	69.2	31.3	1.34	287	69.2	88

[a] - Monomer conversion was calculated by ^1H NMR by comparing integrals of polymers (4.05 ppm) and residual monomers (~ 5.8 – 6.5 ppm). [b] - M_n (theory) = ([LAc]/[BTPA]) × conv. (LAc) × MW(LAc) + MW(BTPA). [c] - THF as eluent with polystyrene as calibration standards. [d] - ^1H NMR (400 MHz, toluene-d8) at 298 K. The degree of polymerization (X_n) of PLAc-CTAs was calculated based on the integral value at 4.05 ppm (the peak a in Fig. S2), $X_n = I_{4.05}/I_{3.10}$. M_n (NMR) = X_n(PLAc-CTA) × MW(LAc) + MW(BTPA). End group fidelity was calculated using equation S16:

$$f\ (\%) = \frac{I_{5.28}}{\left(\frac{I_{3.10}}{2}\right)} \times 100\% \qquad \textbf{(Eq. S16)}$$

where $I_{3.35}$ and $I_{4.8}$ are integral values at 3.10 and 5.28 ppm, respectively, which represent the peaks c and d in Fig. S2.

Aminolysis of $PLAc_{103}$-CTA in the presence of methyl acrylate

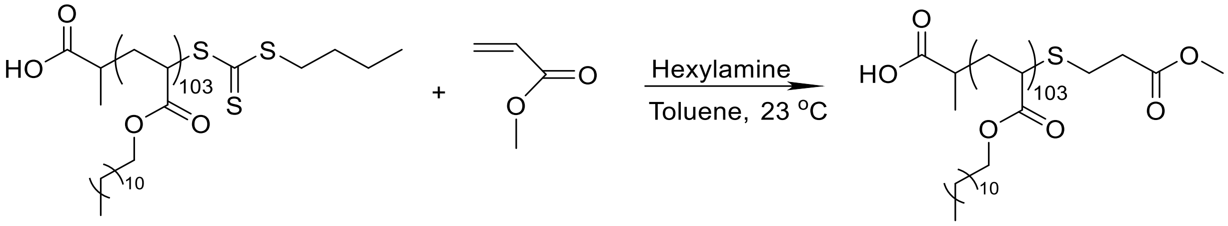

Fig. S2. Synthesis of inert $PLAc_{103}$ via aminolysis of $PLAc_{103}$-CTA in the presence of methyl acrylate.

Protocol: $PLAc_{103}$-CTA (10.95 g, 4.6×10^{-4} mol) was dissolved in toluene (40 mL). The solution was deoxygenated by purging with nitrogen for 60 min. To this solution, methyl acrylate (0.25 mL, 2.7×10^{-3} mol) in 2 mL of toluene was injected via a syringe, followed by the injection of the solution of hexylamine (0.24 mL, 1.8×10^{-3} mol) in 2 mL of toluene. The reaction was allowed to react for 15 h at 22°C. After 15 h, the yellow colour of the RAFT end group was still present, so more methyl acrylate (82 μL, 9.2×10^{-4} mol) and hexylamine (0.72 mL, 5.5×10^{-3} mol) were injected to the reaction solution. The reaction was allowed to react for another 10 h. The reaction solution was then concentrated by rotary evaporation, and the polymer was recovered by precipitation into large excess of acetonitrile, isolated by centrifugation, and then dried under vacuum using rotary evaporation to get a colourless viscous liquid. SEC (RI, THF): M_n = 14.2 kg/mol, $Đ$ = 1.26.

3D printing setup and procedure

A typical procedure for fabricating 3D printed objects is as follows: A 3D object was designed using the Tinkercad 3D modelling software and the object was exported as an .stl file. The .stl file was opened using Photon Workshop where the Z lift speed was set to 3 mm/s and Z retract speed was set to 2 mm/s, while the Z lift distance was set to 6 mm. Printing parameters, such as layer thickness and exposure time, were defined in Photon workshop, sliced, and exported as .pws files for 3D printing. The .pws file copied to a flash drive for use with a masked DLP 3D printer (Anycubic Photon Mono SE) with a violet (λ_{max} = 405 nm) light LED

array (I_0 = 2.0 mW cm^{-2}, as measured at the digital mask surface using a Newport 843-R power meter). For 3D printed samples, the layer thickness was 100 µm, off time was 6 s, layer and bottom exposure times were 60 s, number of bottom layers was 2. Typical 3D printing resin formulations were prepared by combining the calculated amounts of PLAc$_n$-CTA, SMP-10, HDODA, LAc, and BAPO (Table S2). The resin was then added to the 3D printer vat, and the desired print program was run. After 3D printing was completed, the printed objects were separated from the build plate, washed with *n*-hexane, air dried, and post-cured under violet light (λ_{max} = 405 nm) for 40 min.

Pyrolysis of 3D printed preceramic PIMS materials

Pyrolysis was carried out using a tubular furnace GSL-1700X (MTI Corporation). Preceramic materials were first thermally cured (to further cross-link allylic bonds of SMP-10) by heating from room temperature to 160 °C at a rate of 1 °C min^{-1} under argon and held at 160 °C for 2 h in argon. After that, the preceramic materials were heated again from 160 to 230 °C at a rate of 1 °C min^{-1} under argon and held at 230 °C for 2 h in argon. Pyrolysis was carried out by further heating of the preceramic materials to 800 °C at a rate of 1 °C min^{-1} under argon followed by being held at 800 °C for 1 h in flowing argon (100 standard cubic centimetres per minute).

Additional Data

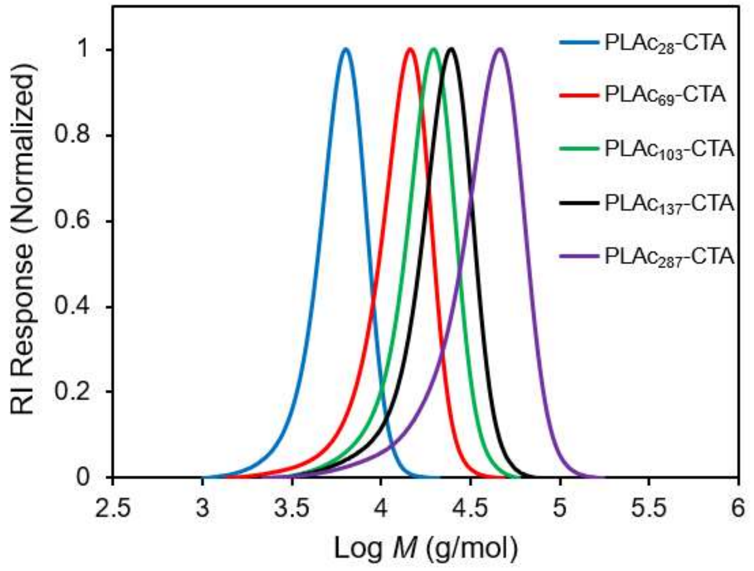

Fig. S1. Molecular weight distributions (MWDs) of PLAc$_n$-CTAs obtained by SEC using a RI detector, with THF as eluent and calibrated using PSTY standards.

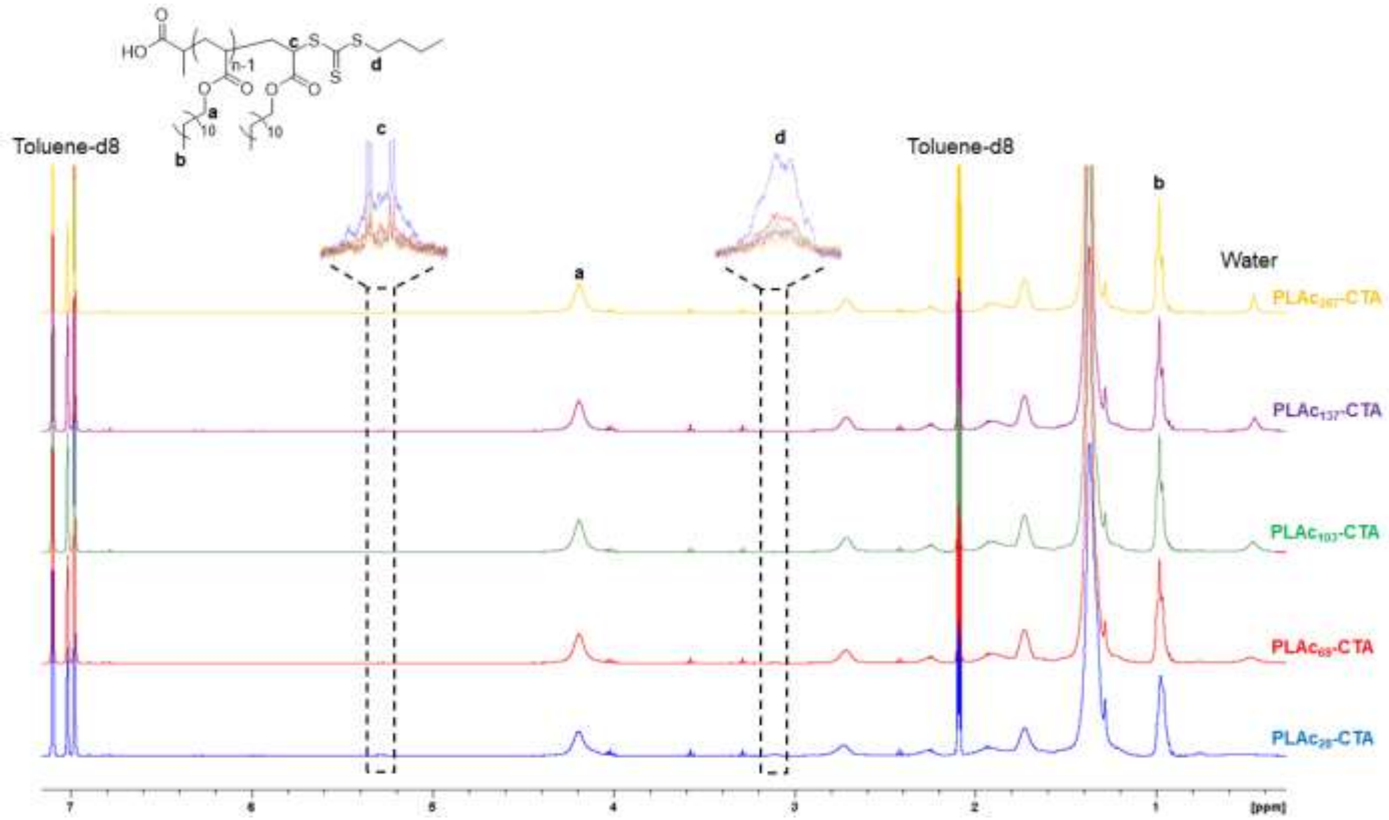

Fig. S2. ^{1}H NMR spectra (400MHz, toluene-d8, 298K) of PLAc$_n$-CTAs. The spectra were normalized by a resonance at 4.05 ppm.

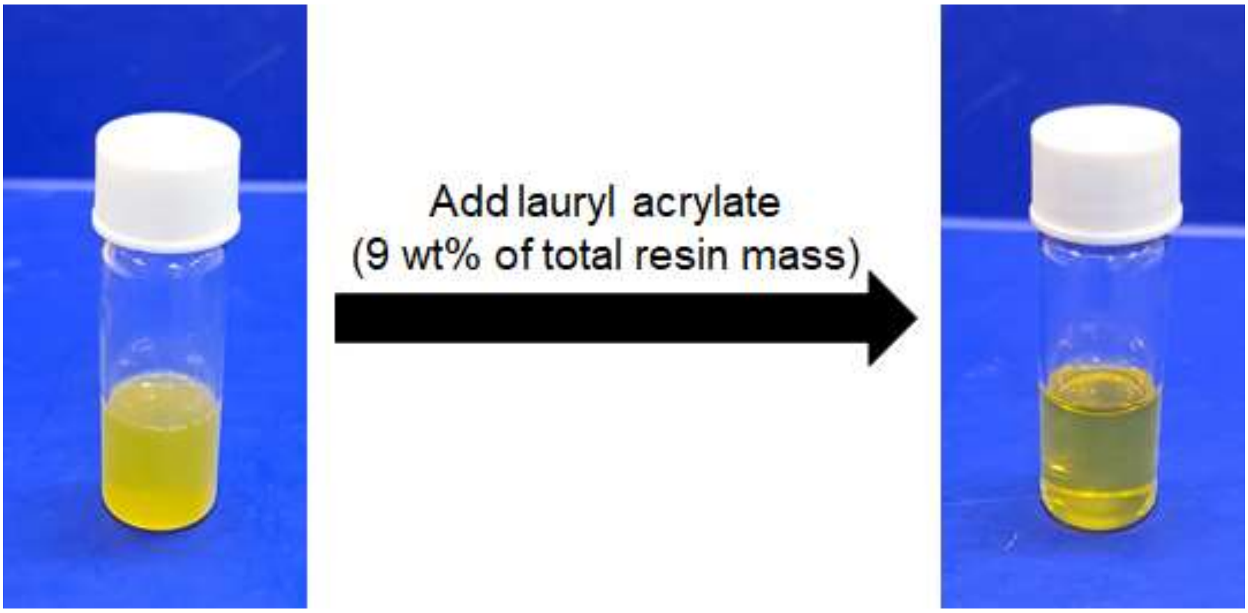

Fig. S3. Representative photos demonstrating that the addition of lauryl acrylate (LAc, 9 wt% of total resin mass) facilitates the solubility of macroCTA (PLAc$_{103}$-CTA) in the mixture of SMP-10/HDODA = 1/1, wt ratio.

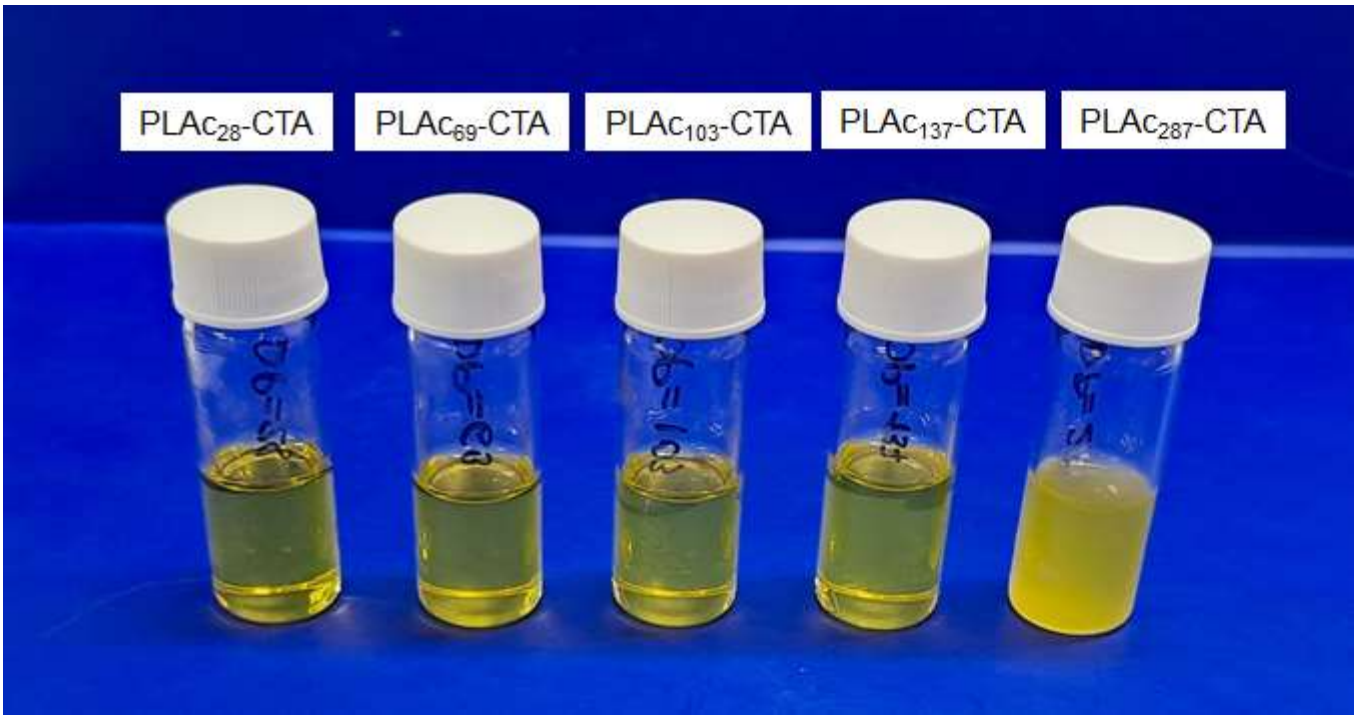

Fig. S4. Photo of resin formulations formed upon mixing all components: SMP-10 (36.9 wt%), HDODA (36.9 wt%), LAc (9.4 wt%), BAPO (1.8 wt%) and PLAc-CTA (14.9 wt%) with various degree of polymerization (X_n), X_n = 28, 69, 103, 137 and 287. The resins formulated using PLAc-CTA X_n = 28, 69, 103 and 137 appeared as homogeneous and optically transparent solutions indicating complete solubilization of the resin components. For the resin formulated using PLAc$_{287}$-CTA, a turbid solution was observed indicating incomplete solubilization of the resin components. This resin formulation was not used for further study.

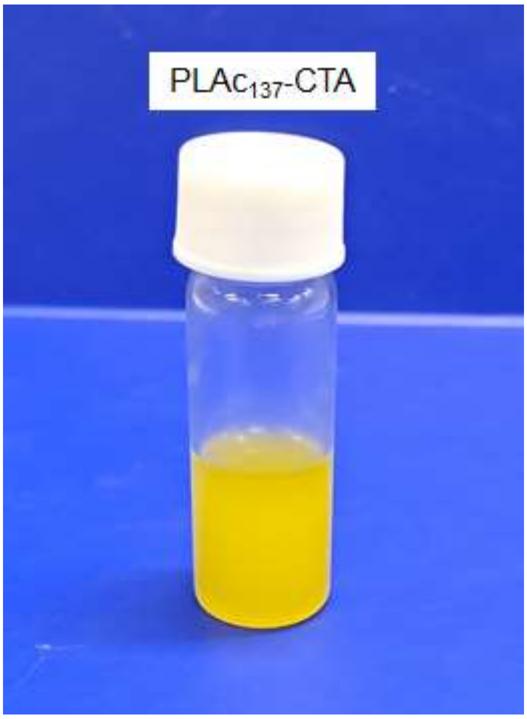

Fig. S5. Photo of resin formulation formed upon mixing all components: SMP-10 (34.4 wt%), HDODA (34.4 wt%), LAc (9.4 wt%), BAPO (1.8 wt%) and PLAc$_{137}$-CTA (20.0 wt%). The resin appeared as a turbid solution

indicating incomplete solubilization of the resin components. This resin formulation was not used for further study.

Table S2. Resin formulations with varying degree of polymerization (X_n) of PLAc-CTA.

Resin #	X_n of PLAc-CTA	Resin component (wt%)						
		SMP-10	HDODA	LAc	PLAc$_n$-CTA	PLAc$_{103}$	BTPA	BAPO
1	28	36.9	36.9	9.4	14.9	-	-	1.8
2	69	36.9	36.9	9.4	14.9	-	-	1.8
3	103	36.9	36.9	9.4	14.9	-	-	1.8
4	137	36.9	36.9	9.4	14.9	-	-	1.8
5	-	36.9	36.9	24.2	-	-	0.1	1.8
6	-	36.9	36.9	24.2	-	-	0.2	1.8
7	-	36.9	36.9	9.4	-	14.8	0.1	1.8
8	-	36.9	36.9	9.4	-	14.9	-	1.8

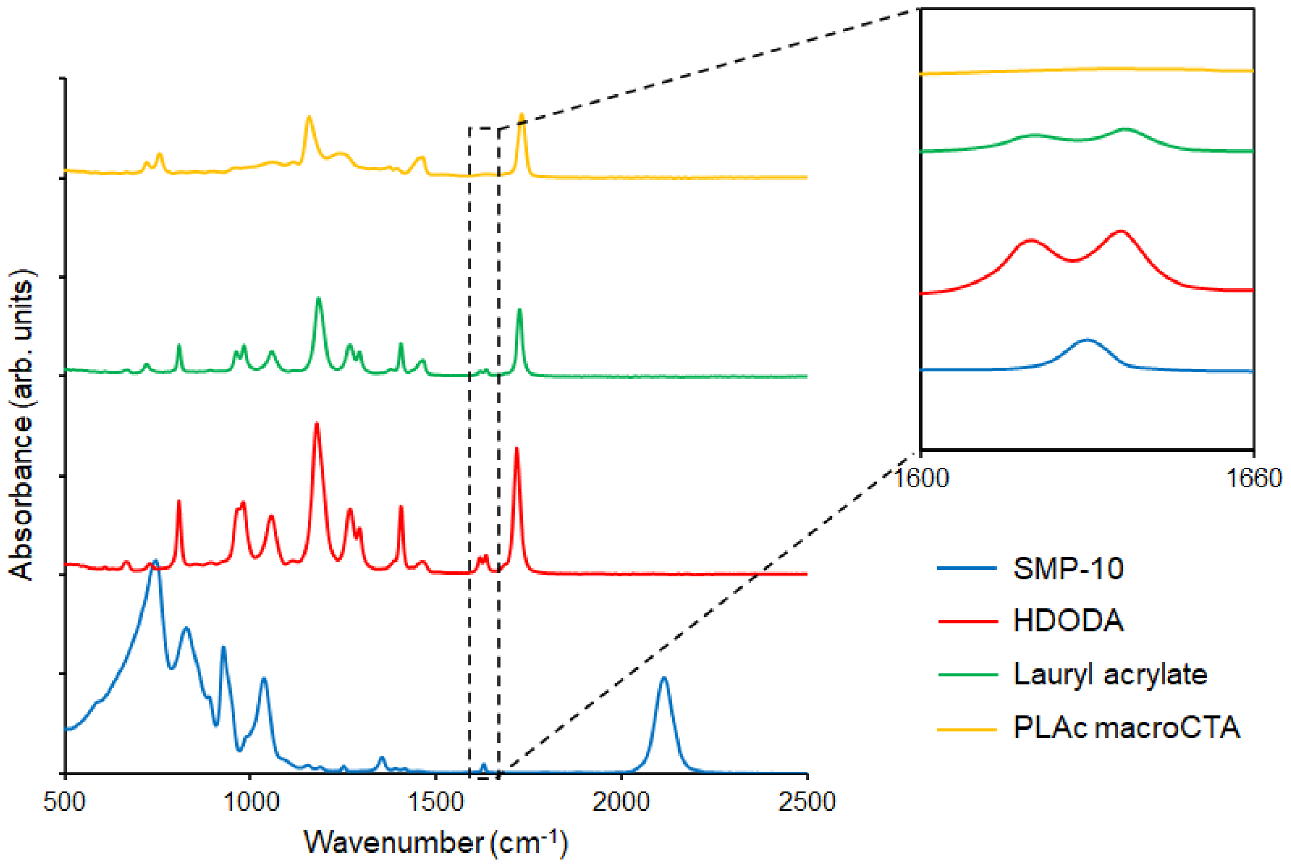

Fig. S6. FTIR spectra of the core resin components: SMP-10, HDODA, lauryl acrylate and PLAc macroCTA. Insert: peak at 1630 cm^{-1} (blue line) indicates Si-C-C=C allyl overtone. Peak at 1600-1650 cm^{-1} indicates C=C stretching overtone of HDODA and lauryl acrylate.

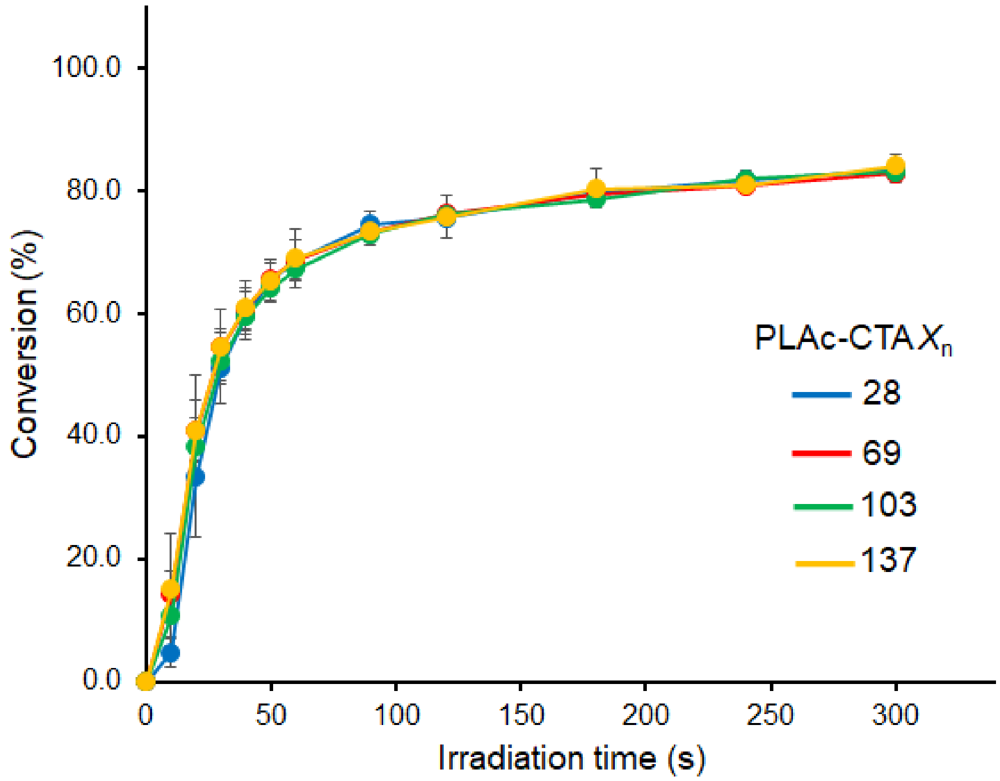

Fig. S7. Polymerization kinetics of preceramic resins under open-to-air conditions. The resins were formulated upon mixing all components: SMP-10 (36.9 wt%), HDODA (36.9 wt%), LAc (9.4 wt%), BAPO (1.8 wt%) and PLAc-CTA (14.9 wt%) with various degree of polymerization (X_n), X_n = 28, 69, 103, 137 and 287. Double bond conversions were determined using ATR-FTIR under 2.06 mW cm^{-2} violet light (λ_{max} = 405 nm). Error bars indicate standard deviation in triplicate measurements. Some error bars fall within the size of the markers.

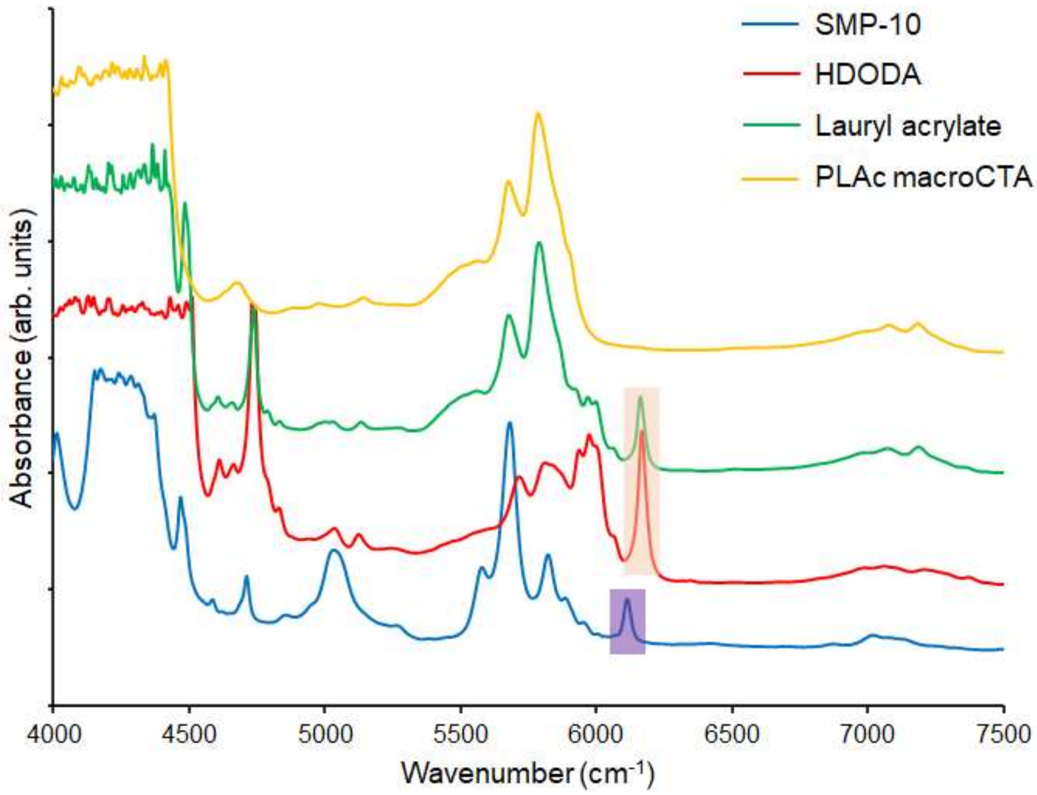

Fig. S8. FTNIR spectra of core resin components: SMP-10, HDODA, lauryl acrylate and PLAc macroCTA. The C-H vinylic stretching overtone (6120-6220 cm^{-1}) of HDODA and lauryl acrylate is highlighted by transparent orange box. The C-H allylic stretching overtone (6080-6150 cm^{-1}) of SMP-10 is highlighted by transparent purple box. The FTNIR spectra were shifted vertically for clarity.

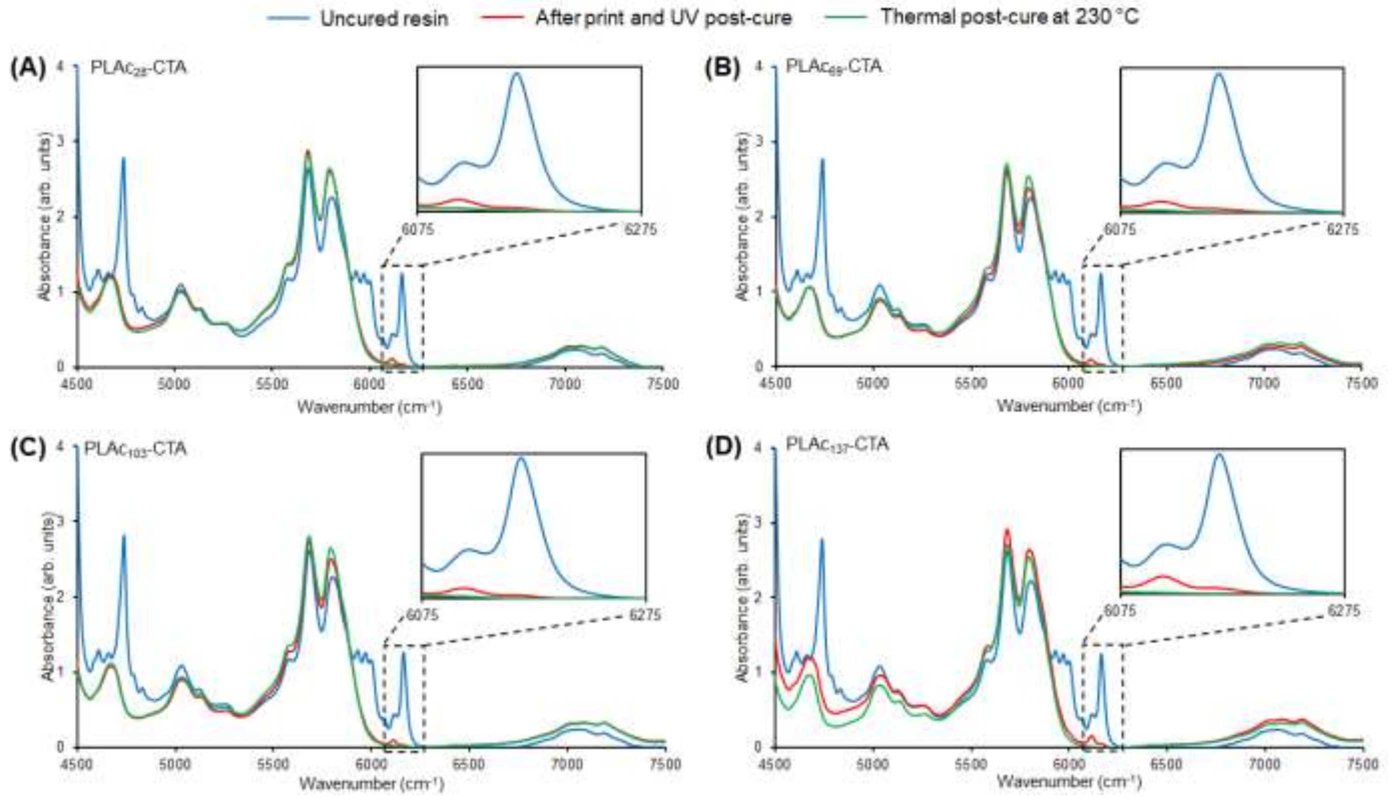

Fig. S9. FTNIR spectra of an uncured resin, a solid 3D printed preceramic material after UV (405 nm) and thermal (160 and 230 °C) post-curing prepared using PLAc-CTA with X_n = **(A)** 28, **(B)** 69, **(C)** 103, and **(D)** 137. Insert: peak at 6080-6220 cm^{-1} which corresponds to the C-H vinylic and allylic overtones decreased upon photoinduced 3D printing and subsequent thermal post-curing, indicating full conversion of vinylic and allylic bonds.

Table S3. SMP-10 and acrylates conversion calculated based on the FTNIR data.

Resin #	X_n of PLAc-CTA	Treatment	Conversion (%)	
			SMP-10	Acrylates (HDODA + LAc)
1	28	After print and UV post-cure	66	99
		After thermal post-cure (230 °C)	97	100
2	69	After print and UV post-cure	67	99
		After thermal post-cure (230 °C)	98	100
3	103	After print and UV post-cure	66	99
		After thermal post-cure (230 °C)	99	100
4	137	After print and UV post-cure	55	98
		After thermal post-cure (230 °C)	99	100

Note: conversion values were calculated using equation S2 (see Characterization methods section).

A

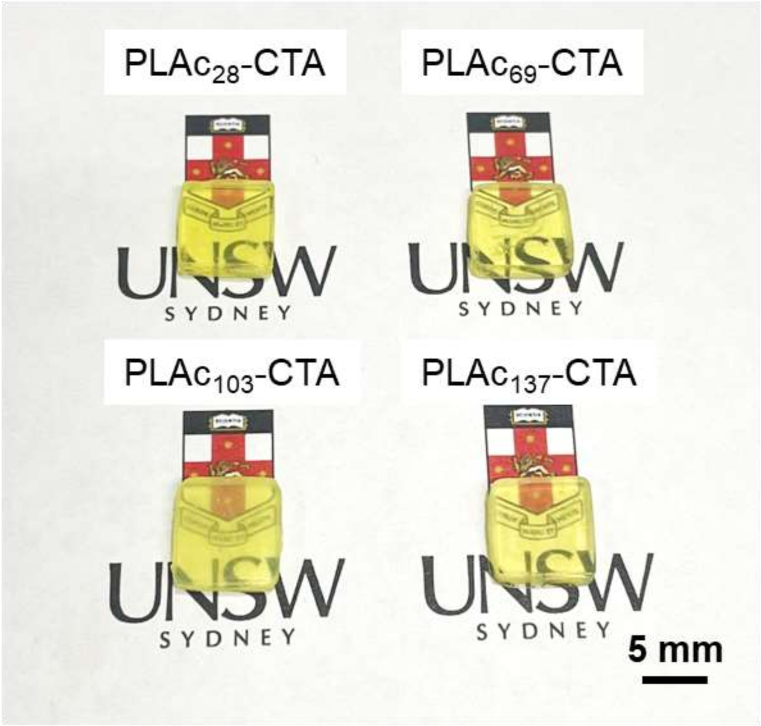

B

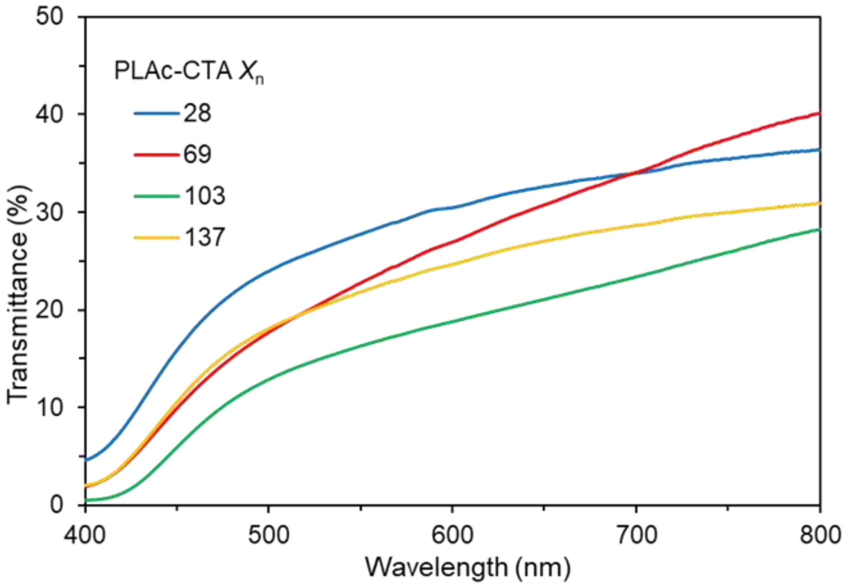

Fig. S10. (**A**) Photo showing preceramic materials 3D printed with various PLAc-CTA X_n. (**B**) Transmittance from 400-800 nm for the preceramic materials 3D printed with various PLAc-CTA X_n. The thickness of 3D printed samples is 3 mm.

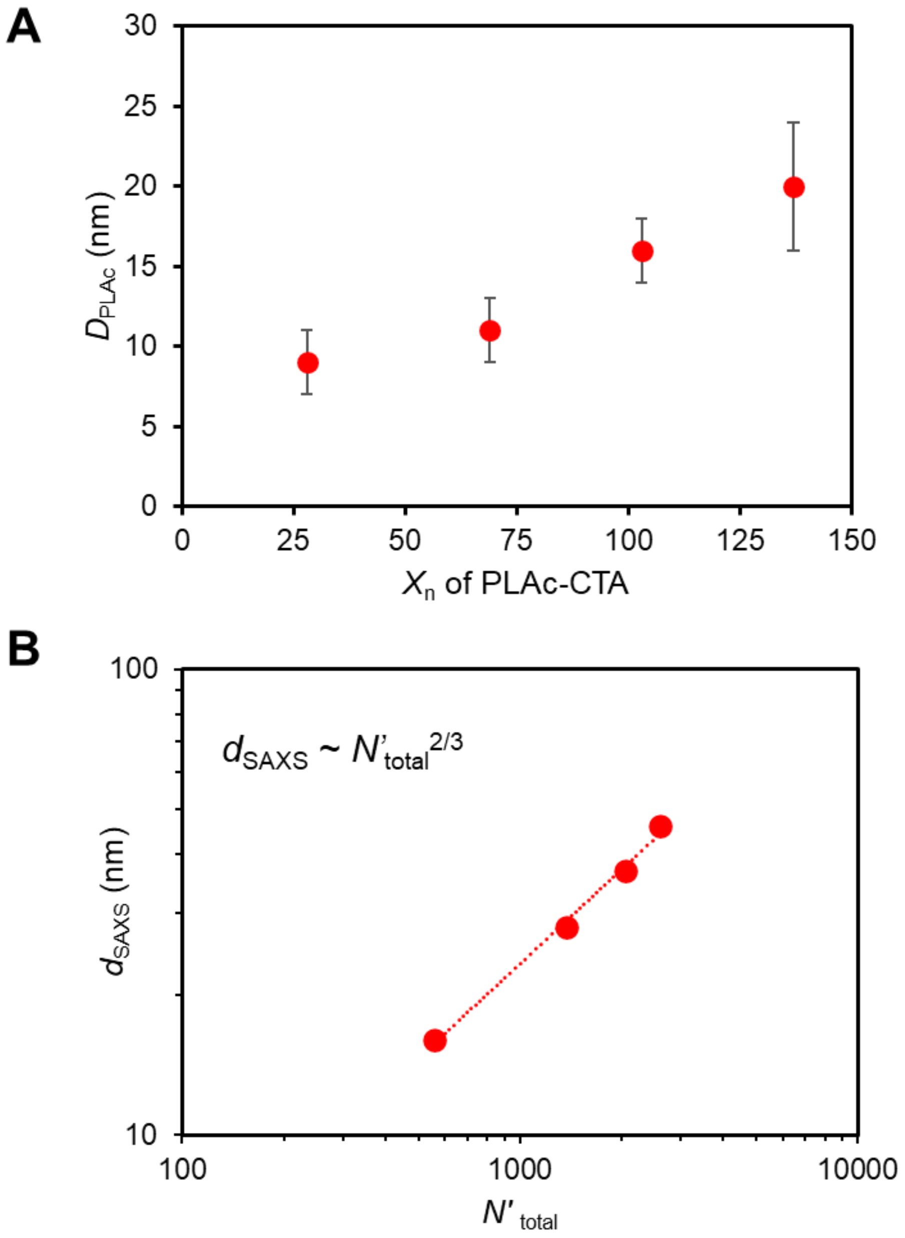

Fig. S11. (A) Dependence of a PLAc-CTA domain size (D_{PLAc}) as a function of PLAc degree of polymerization (X_n). D_{PLAc} values were measured manually by analysing AFM images using the ImageJ software. Error bars represent the standard deviation of at least 50 measurements. **(B)** Power law scaling for domain spacings of 3D printed PIMS preceramic materials. Log-log plot of domain spacing (d_{SAXS}) as a function of total degree of polymerization (N'_{total}). d_{SAXS} determined from SAXS. N'_{total} was calculated based on a common monomer reference volume (118 Å^3).

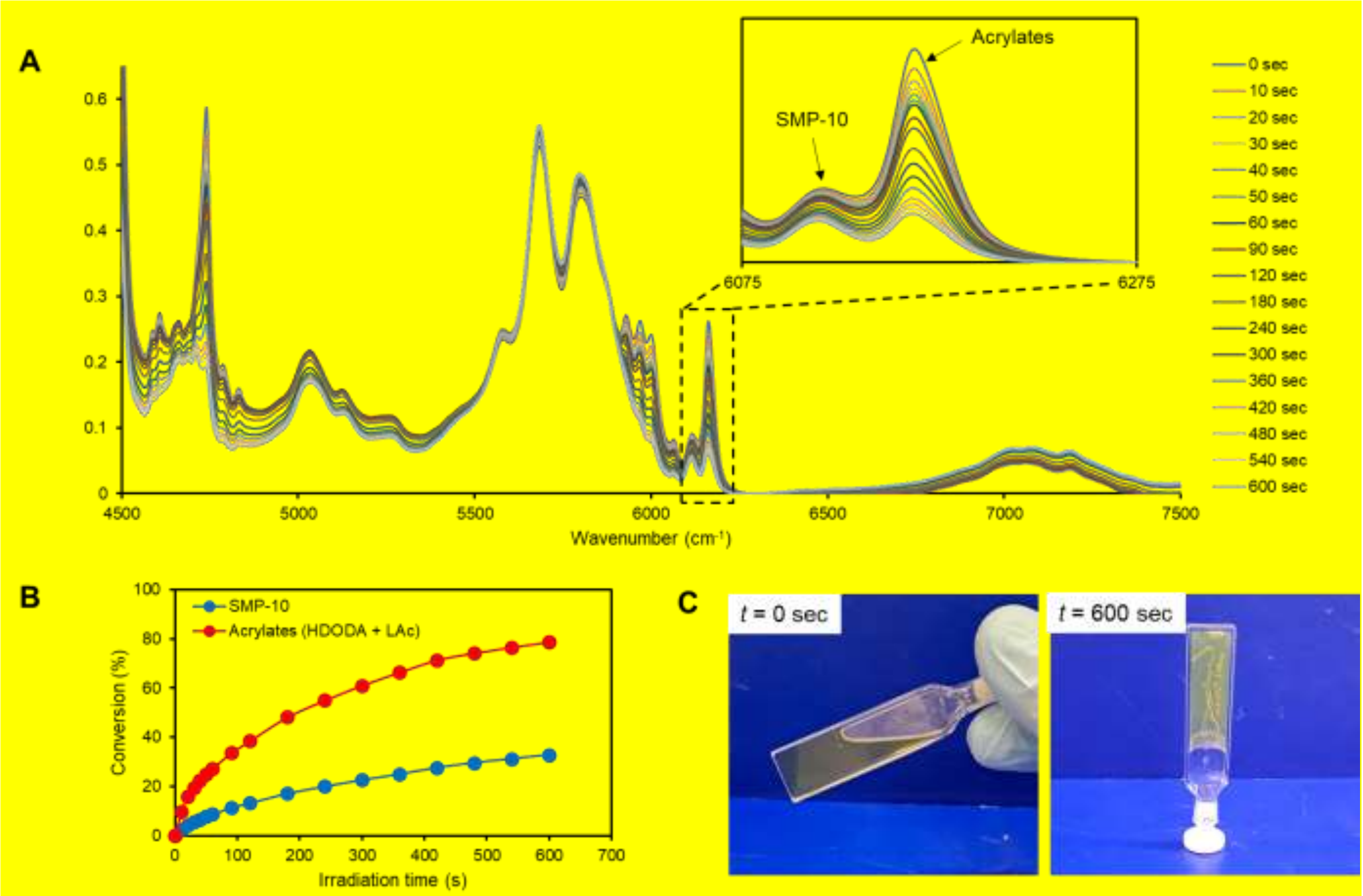

Fig. S12. Kinetics of the preceramic resin formulated with $PLAc_{103}$-CTA (resin #3, Table S2) monitored by FTNIR. The resin (without prior degassing) was exposed to 2.06 mW cm^{-2} violet light ($\lambda_{max} = 405$ nm). (**A**) FTNIR spectra of the resin over time. (**B**) Conversion profiles of SMP-10 and acrylates (HDODA and LAc). (**C**) Photos of the resin at the beginning and the end of the photopolymerization kinetics monitoring.

<u>Note</u>: The conversions of allyl bonds of SMP-10 and vinyl bonds of HDODA and LAc were monitored by FTNIR. As it is technically challenging to monitor the consumption of these bonds during 3D printing, we performed model kinetics reactions by placing the resin in quartz a cuvette. More specifically, we introduced 600 μL of the resin (SMP-10 (36.9 wt%), HDODA (36.9 wt%), LAc (9.4 wt%), BAPO (1.8 wt%) and PLAc103-CTA (14.9 wt%)) into a quartz cuvette with a path length of 2 mm. Subsequently, we obtained an absorption spectrum by scanning the resin from 4000 to 8000 cm^{-1}. After taking an initial reading, we irradiated the resin using a Thorlabs mounted LED with a collimation adapter (λ_{max} = 405 nm, I_0 = 2.06 mW cm^{-2}) and subsequently the absorption spectra were obtained at various time points to determine vinyl and allyl bonds conversions by comparing the integrals of the C-H vinylic stretching overtone at 6120-6220 cm-1 and the C-H allylic stretching overtone at 6080-6150 cm^{-1} at x min of irradiation. It can be seen from Fig. S12B that vinyl bonds of HDODA and LAc were consumed faster than allyl bonds of SMP-10, reaching 79% after 600 s, while SMP-10 conversion was 33%.

We obtained similar results for the conversion of SMP-10 and acrylates (HDODA and LAc) using 3D printed samples (see Table S3 (resin #3)), however, the final conversions of SMP-10 (66%) and acrylates (99%) were higher than those of the irradiated resin in a quartz cuvette. This distinction arises due to differences in photocuring conditions between the FTNIR kinetics study and 3D printing, such as variations in light intensity and distance to a light source.

The difference in conversion profiles for SMP-10 and acrylates (HDODA and LAc) may lead to the co-existence of PLAc-*b*-P(LAc-*stat*-HDODA), unreacted SMP-10, and PLAc-*b*-P(SMP-10-*stat*-HDODA-*stat*-LAc) species in the polymerization mixture in the early stage. It is possible that PLAc-*b*-P(LAc-*stat*-HDODA) and unreacted SMP-10 species can selectively swell PLAc and *net*-P(SMP-10-*stat*-HDODA-*stat*-LAc) nanodomains, respectively, potentially contributing to a slightly larger scaling exponent (δ = 2/3) for domain spacings (Fig. S11B) than the scaling exponent reported for other PIMS systems (δ ~ 3/5).[6]

Table S4. Parameter values obtained from fitting of SAXS peaks using the Teubner-Strey (T-S) model.

X_n of PLAc-CTA[a]	a_2[b]	c_1[b]	c_2[b]	d_{SAXS} (nm)[c]	d_{TS} (nm)[d]	ξ (nm)[e]	ξ/d_{TS}[f]	f_a[g]
28	12.3	-45.6	226.9	16	15	4	0.25	-0.43
69	23.0	-390.4	4886.7	28	27	8	0.31	-0.58
103	18.4	-434.7	8986.2	37	34	10	0.29	-0.53
137	14.9	-475.8	16374.1	46	42	11	0.27	-0.48

[a] – the degree of polymerization of PLAc-CTA; [b] – parameters calculated from SAXS fitting using the T-S model; [c] – domain spacing determined from SAXS; [d] – domain spacing determined from T-S fitting using Equation S5; [e] – correlation length determined from T-S fitting using Equation S6; [f] – The ratio of ξ/d_{TS} is a measure of the domain size polydispersity, the smaller the ratio, the larger the polydispersity, [g] – amphiphilicity factor determined using Equation S7.

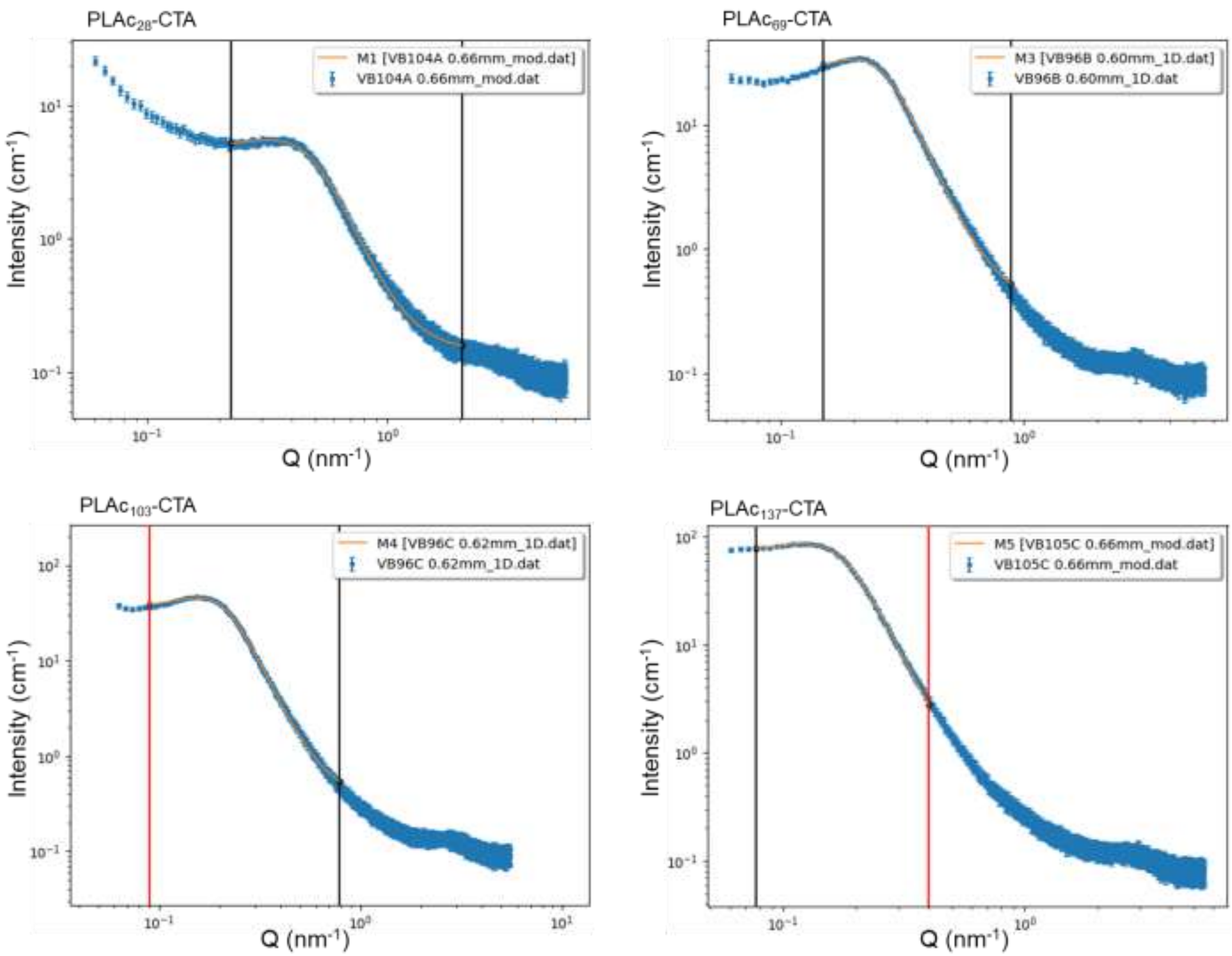

Fig. S13. SAXS peaks fitted using the Teubner-Strey (T-S) model for preceramic samples printed using PLAc$_n$-CTAs. The SAXS scattering data are presented as blue curves and the T-S fits are presented as orange curves.

Note: The additional low-intensity peaks centered around $q \sim 2.8$ nm^{-1}, corresponding to $d_{SAXS} = 2.2$ nm, was observed in the SAXS spectra. This scattering peak can arise due to the electron density contrast between the SMP-10 and HDODA-*stat*-LAc within *net*-P(SMP-10-*stat*-HDODA-*stat*-LAc) domains.

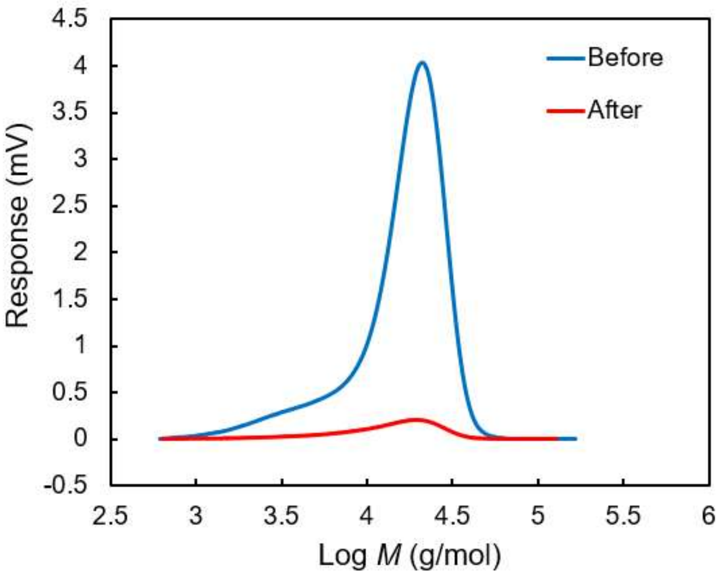

Fig. S14. MWDs of $PLAc_{103}$-CTA before aminolysis (blue line) and after aminolysis (red line) obtained from the UV detector operating at 314 nm. Eluent THF, PSTY standards. The concentration for both samples was 1 mg/mL. The cleavage efficiency was determined to be ≈95%.

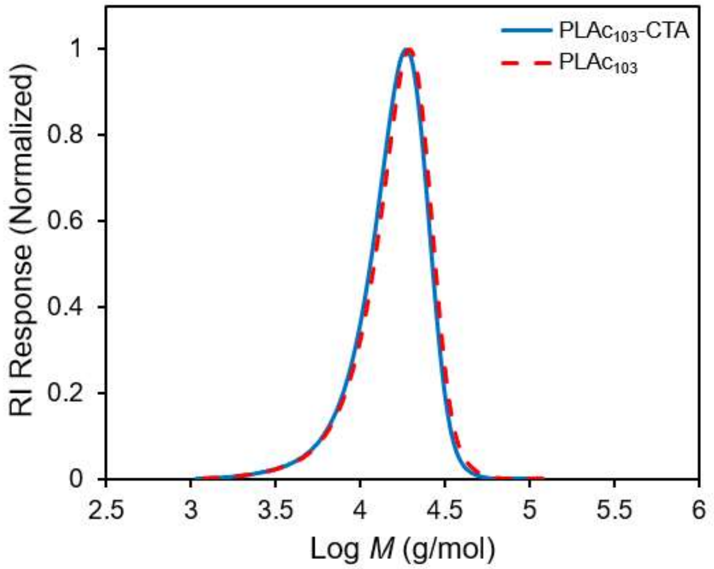

Fig. S15. MWDs of $PLAc_{103}$-CTA before aminolysis (blue line) and after aminolysis (red dashed line) obtained by SEC using a RI detector, with THF as an eluent and calibrated using the PSTY standards.

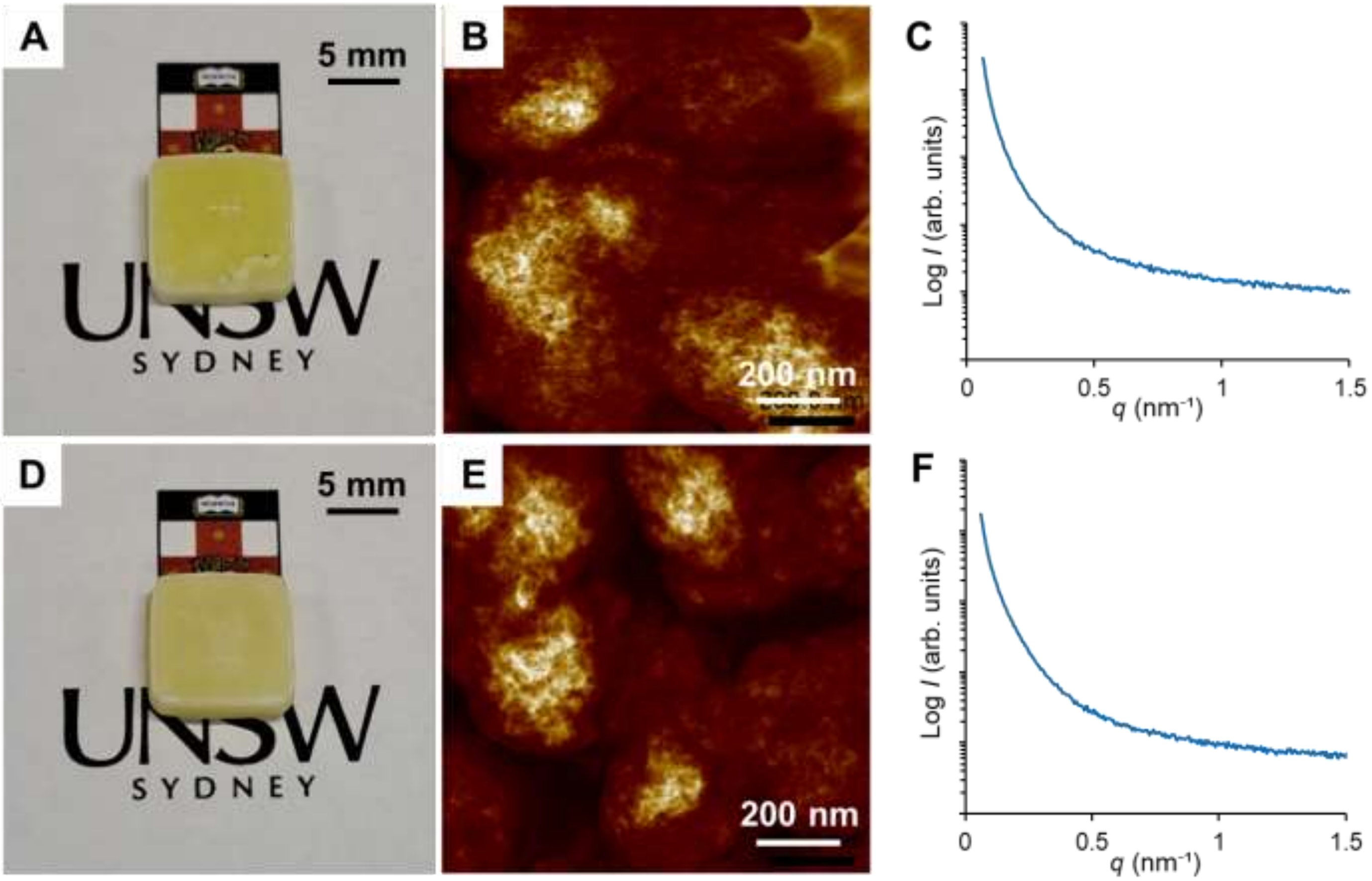

Fig. S16. Characterization of preceramic materials 3D printed in the absence of PLAc-CTA. (**A – C**) Materials 3D printed using the following resin: SMP-10/HDODA/LAc/PLAc$_{103}$/BTPA/BAPO = 36.9/36.9/9.4/14.8/0.1/1.8 (wt%); (**D – F**) SMP-10/HDODA/LAc/PLAc$_{103}$/BAPO = 36.9/36.9/9.4/14.9/1.8 (wt%). (**A**, **D**) Photos demonstrating the physical appearance of the 3D printed objects; (**B**, **E**) PeakForce QNM modulus maps; (**C**, **F**) SAXS profiles.

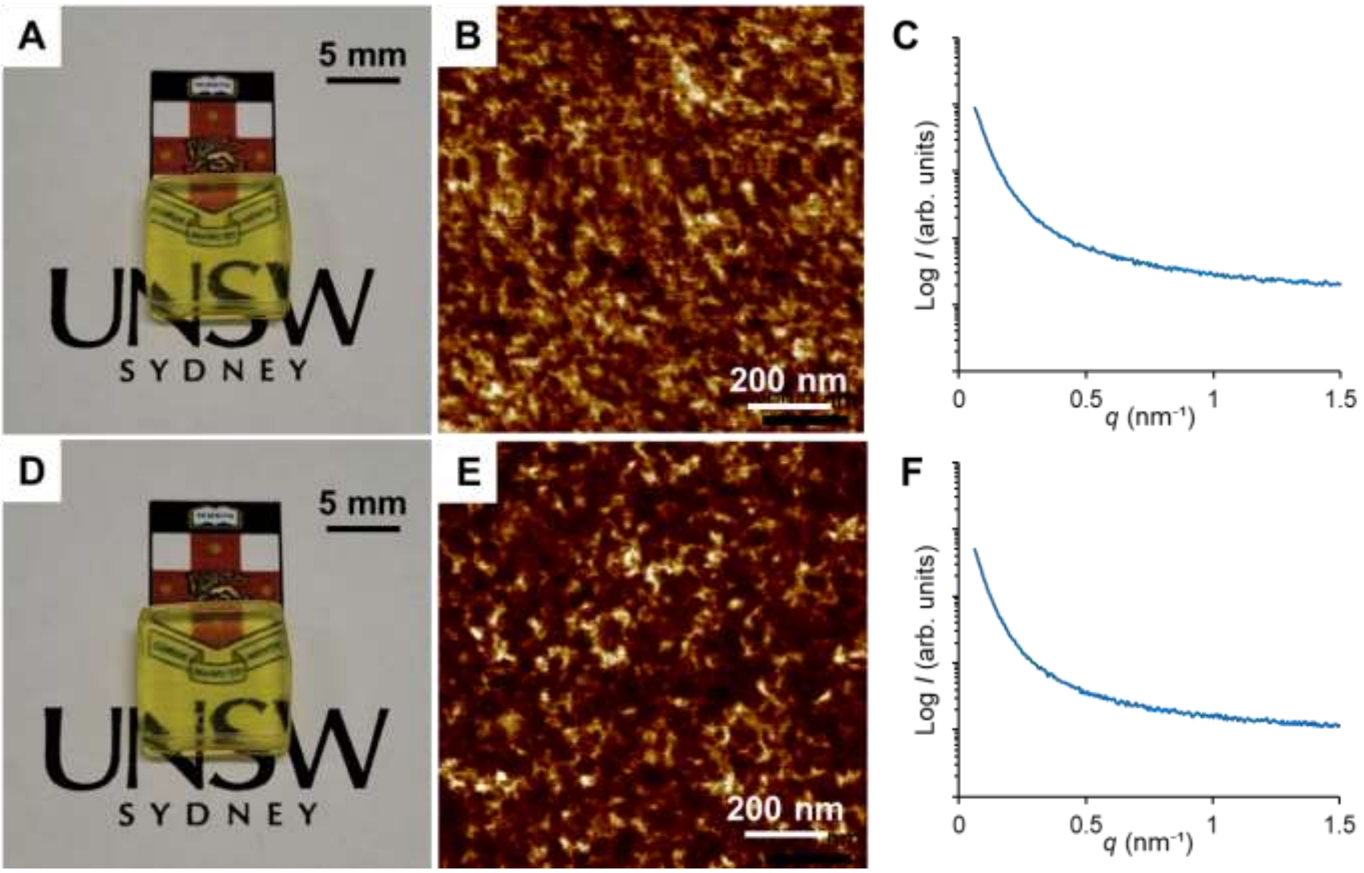

Fig. S17. Characterization of preceramic materials 3D printed in the absence of PLAc-CTA. (**A** – **C**) Materials 3D printed using the following resin: SMP-10/HDODA/LAc/BTPA/BAPO = 36.9/36.9/24.2/0.1/1.8 (wt%); (**D** – **F**) SMP-10/HDODA/LAc/BTPA/BAPO = 36.9/36.9/24.2/0.2/1.8 (wt%). (**A**, **D**) Photos demonstrating the physical appearance of the 3D printed objects; (**B**, **E**) PeakForce QNM modulus maps; (**C**, **F**) SAXS profiles.

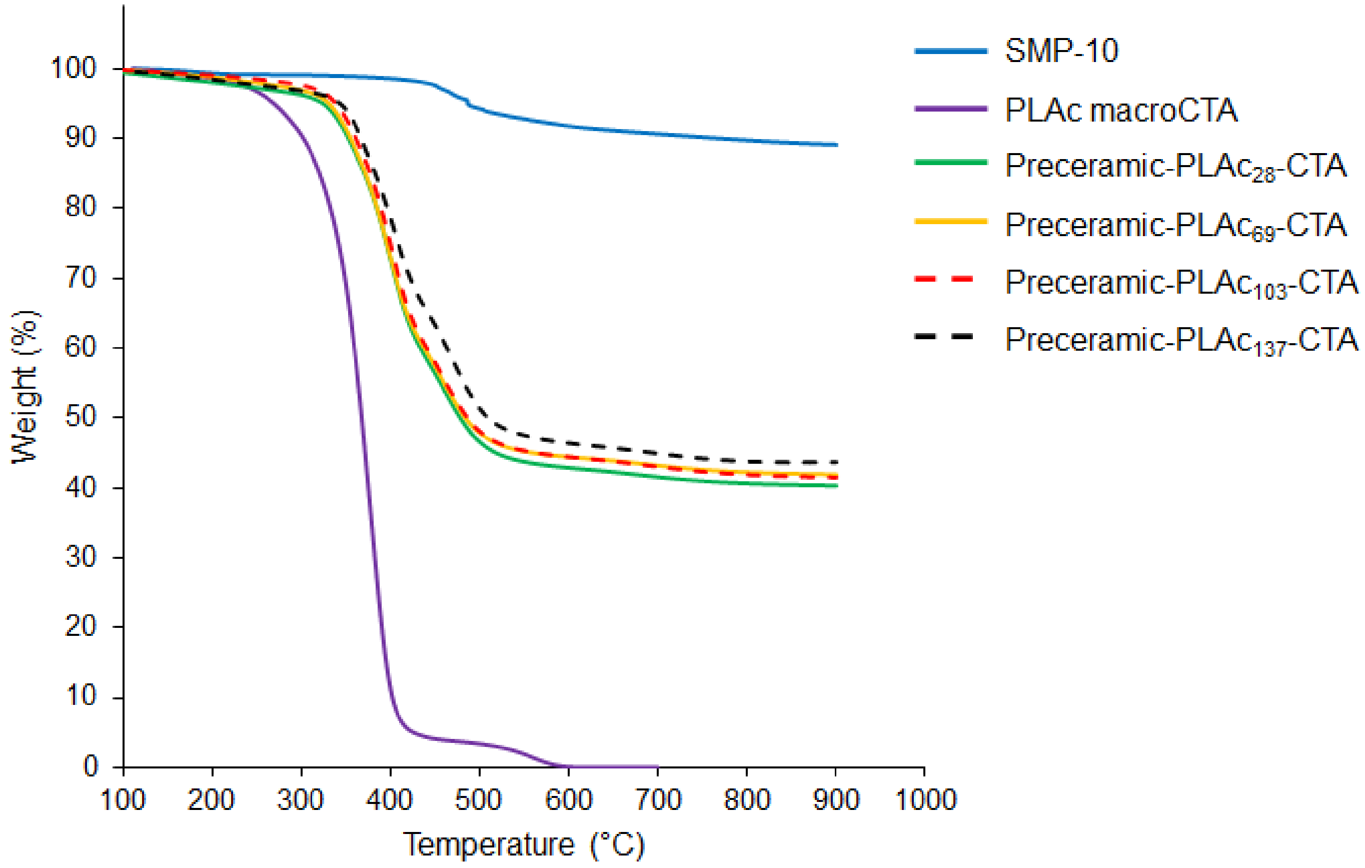

Fig. S18. TGA profiles of SMP-10, PLAc macroCTA (X_n = 137) and 3D printed preceramic PIMS materials prepared using PLAc-CTA with X_n = 28, 69, 103 and 137. The heating rate was 10 °C min^{-1} under nitrogen.

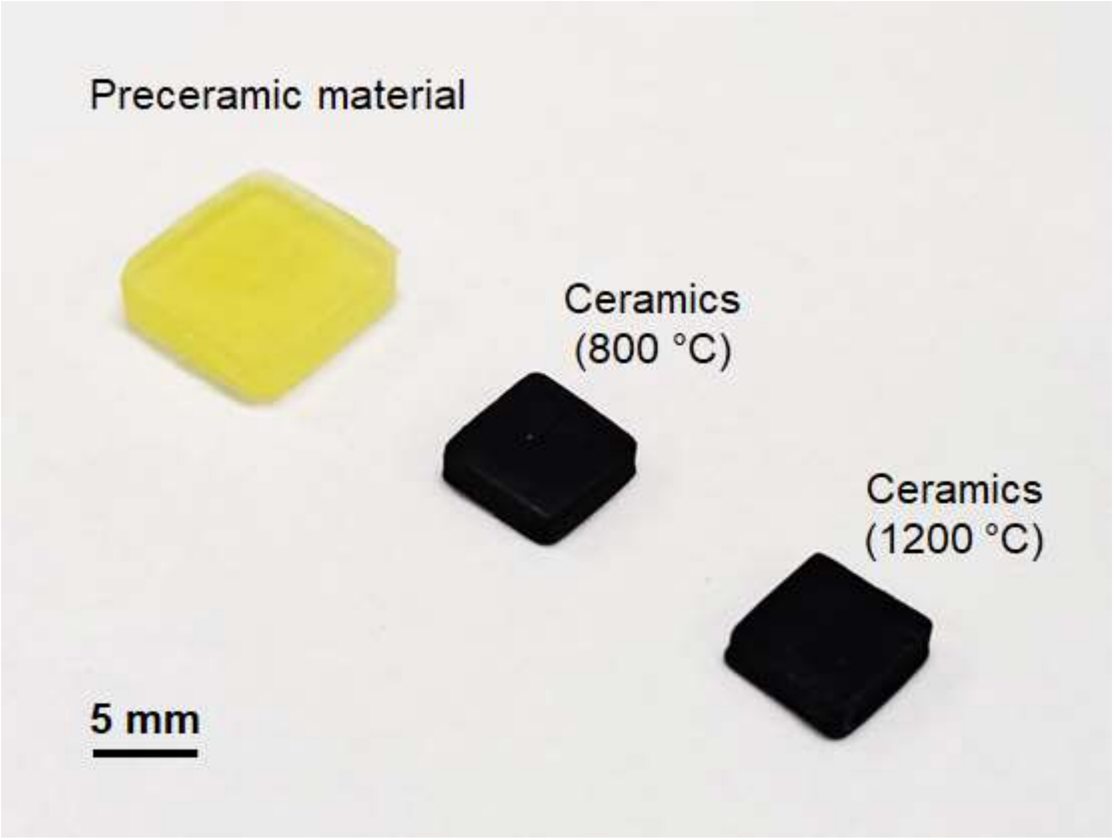

Fig. S19. Representative photo demonstrating physical appearance of 3D printed PIMS preceramic materials before and after pyrolysis (at 800 and 1200 °C). Material retained shape upon pyrolysis with no noticeable deformation.

Table S5. The extent of mass loss and linear shrinkage upon pyrolysis of 3D printed preceramic PIMS materials prepared using PLAc-CTA with various X_n values.

**X_n of PLAc-CTA**	**Pyrolysis temperature (°C)**	**% mass remained**	**Linear shrinkage (%)**		
			**x**	**y**	**z**
28	800	43	31	31	31
	1200	44	35	36	33
69	800	46	31	31	31
	1200	43	33	34	33
103	800	43	30	30	30
	1200	43	34	35	34
137	800	44	30	30	30
	1200	43	34	34	34

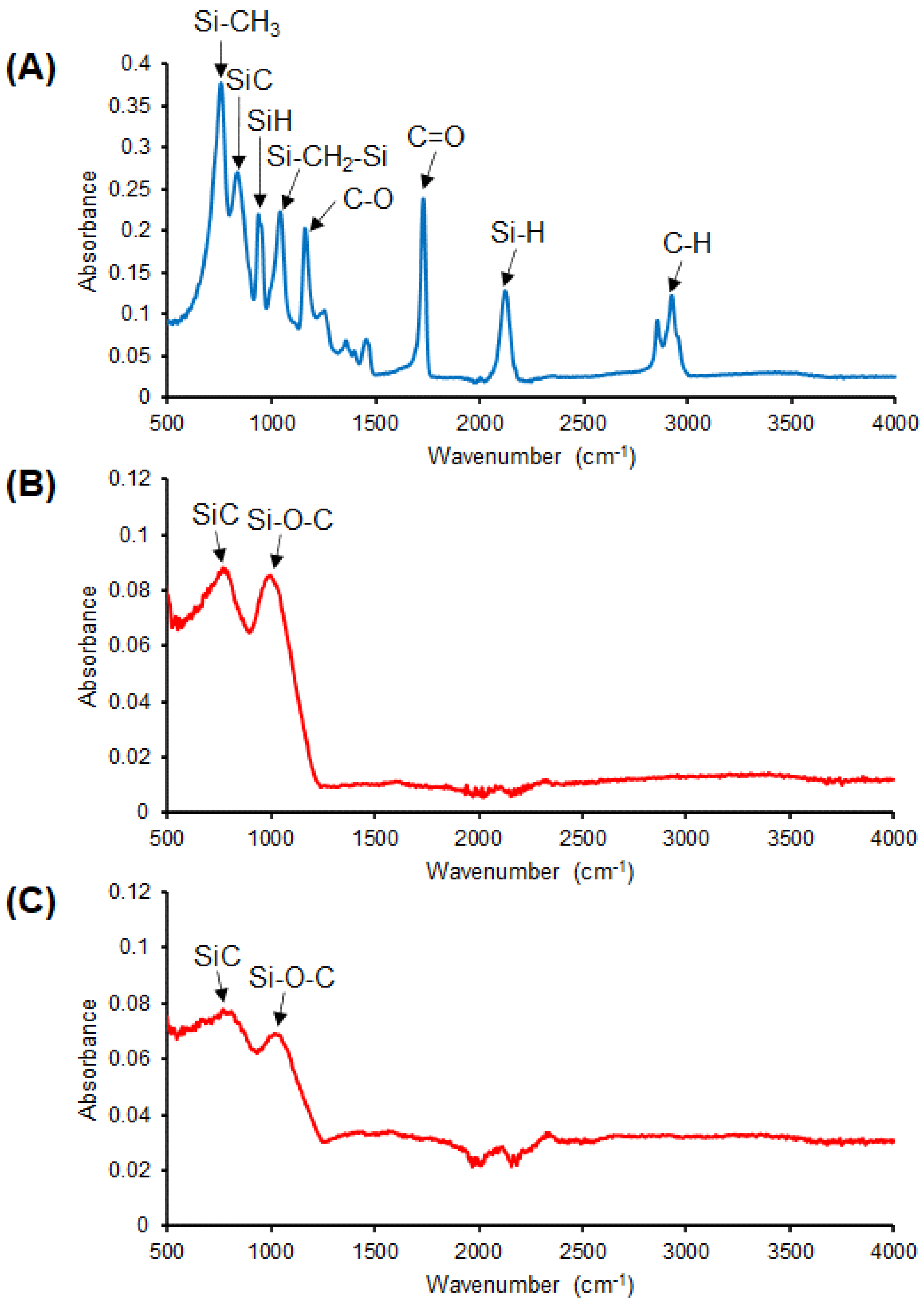

Fig. S20. FTIR spectra of 3D printed preceramic material prepared using $PLAc_{137}$-CTA. **(A)** before pyrolysis, **(B)** after pyrolysis at 800 °C, and **(C)** after pyrolysis at 1200 °C. The low intensity of the ceramic sample spectrum is attributed to the rigid nature of ceramics.[7]

Table S6. Assignments of the NEXAFS peaks observed in the spectra of ceramic materials pyrolyzed at 800 °C.

Edge	Peak	Energy (eV)	Bond assignment	Reference
Si K-edge	Si1	1845	sp^3 ($\sigma^*_{Si\text{-}C}$)	[8]
	Si2	1847	sp^3 ($\sigma^*_{Si\text{-}O}$)	

C K-edge	C1	284.3	sp^2 ($\pi^*_{C=C}$)	[8c, 8e, 9]
	C2	287.0	sp^3 ($\sigma^*_{C\text{-}H}$)	[9b]
	C3	288.4	sp^3 ($\pi^*_{C\text{-}O}$)	[8e, 10]
	C4	289.7	sp^3 ($\sigma^*_{C\text{-}C}$) and/or sp^3 ($\pi^*_{C\text{-}O}$)	[8c, 8f, 9a, 10]
	C5	291.7	sp^3 ($\sigma^*_{C\text{-}Si}$) and/or sp^3 ($\sigma^*_{C\text{-}C}$)	[8c, 8e]
	C6	299.7	sp^3 ($\sigma^*_{C\text{-}Si}$)	[8c, 8e]

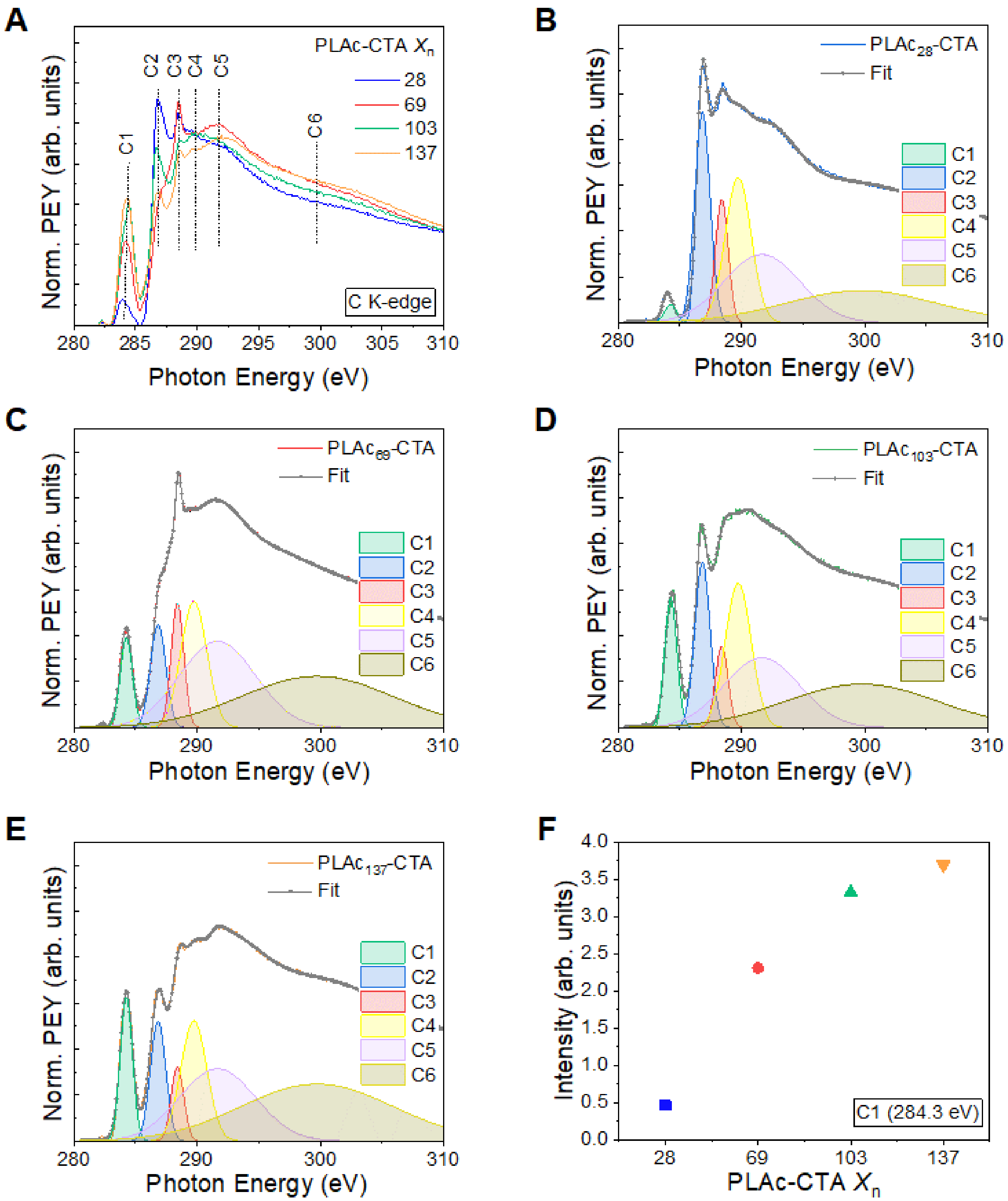

Fig. S21. (**A**) C K-edge NEXAFS of ceramic materials pyrolyzed at 800 °C. Deconvoluted C K-edge NEXAFS spectra of the ceramic materials 3D printed with PLAc-CTA X_n = (**B**) 28, (**C**) 69, (**D**) 103 and (**E**)

137. Assignments of the peaks are given in Table S6 (SI). (**F**) The dependence of C1 (284.3 eV) peak area assigned to sp^2 bonded C on PLAc-CTA with X_n = 28, 69, 103 and 137 pyrolyzed at 800 °C.

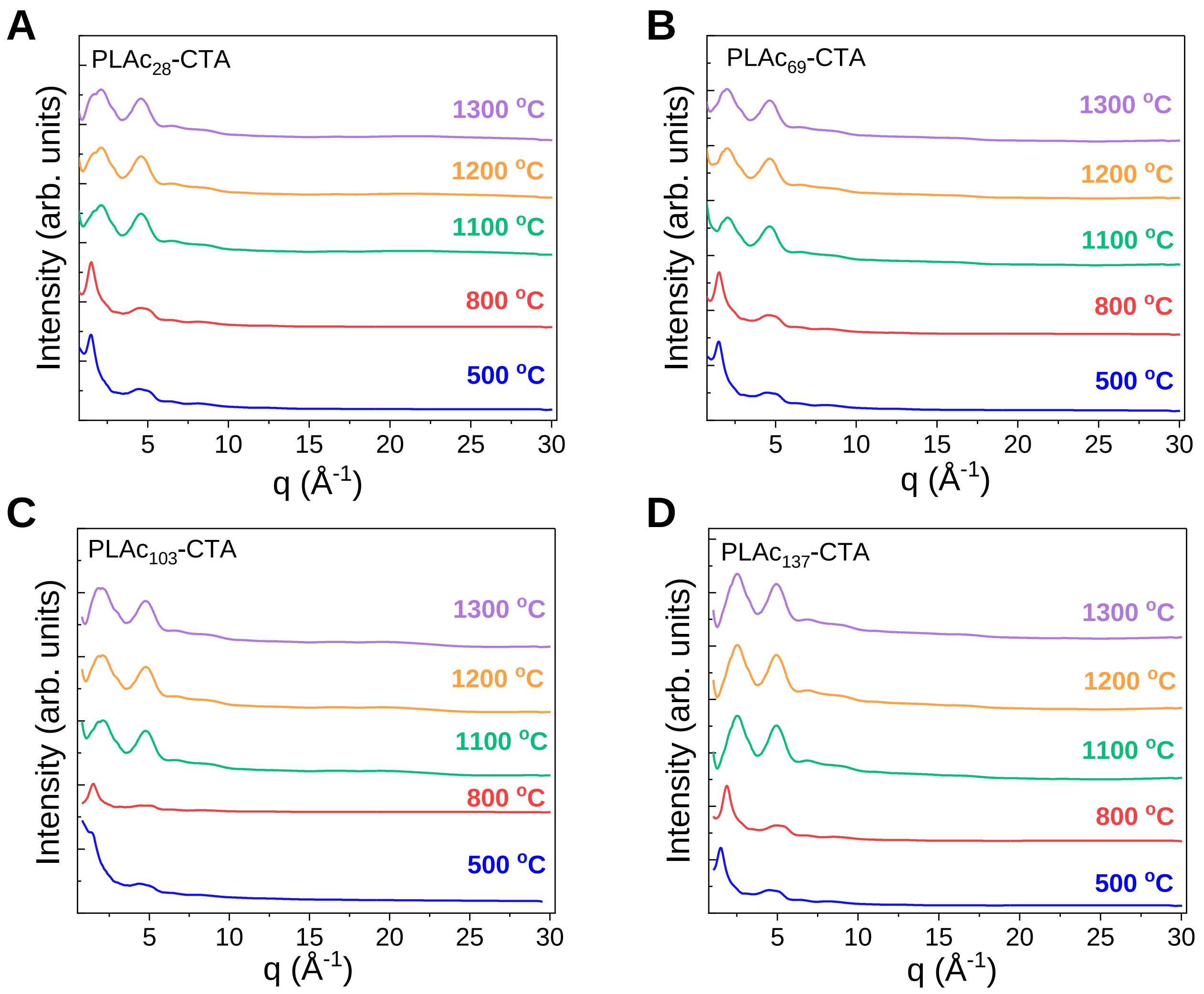

Fig. S22. High-energy X-ray diffraction (HE-XRD) data from synchrotron X-ray study of ceramics materials prepared using PLAc-CTA X_n = (**A**) 28, (**B**) 69, (**C**) 103, and (**D**) 137.

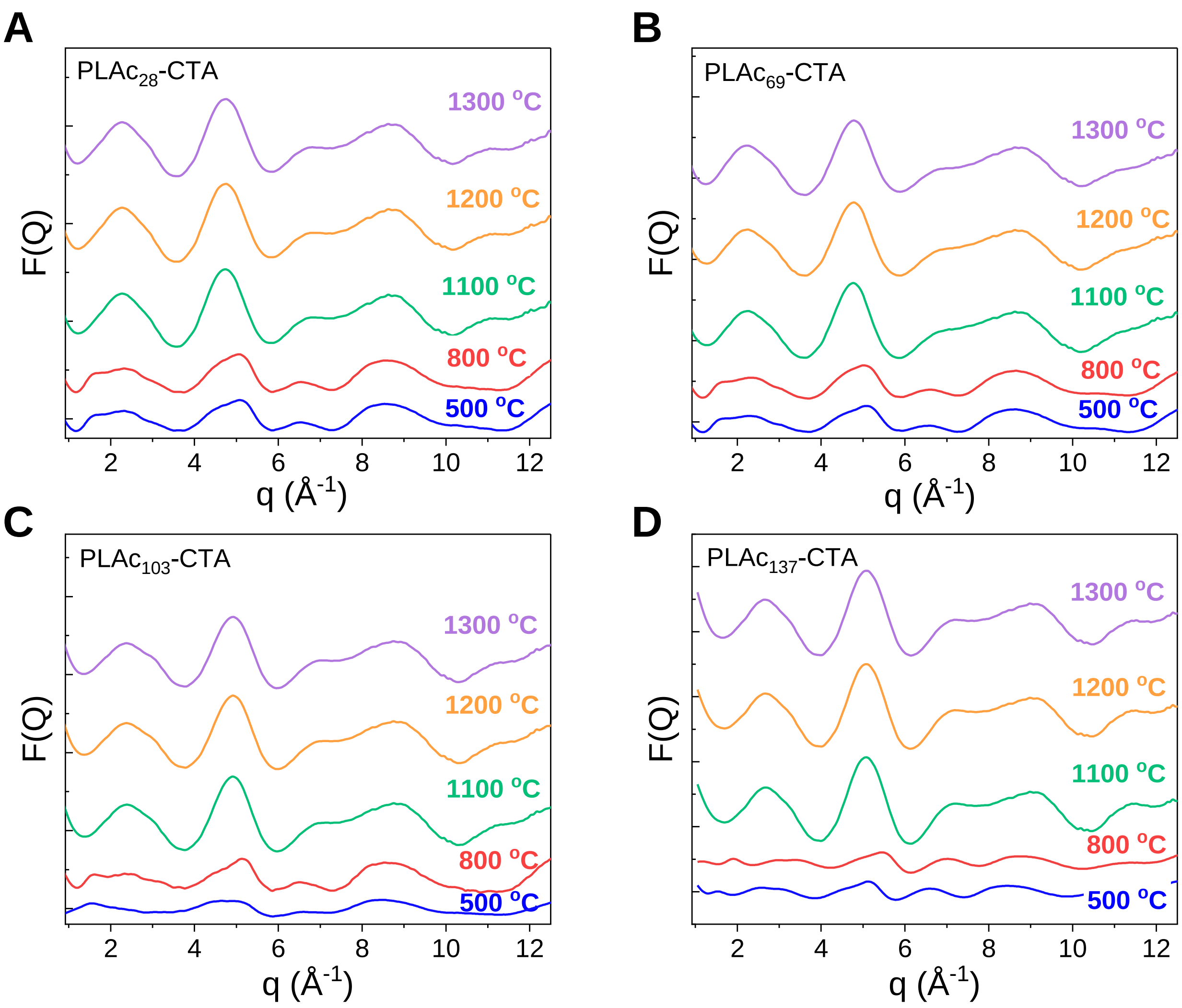

Fig. S23. The structure functions F(Q) of ceramics materials prepared using PLAc-CTA X_n = (**A**) 28, (**B**) 69, (**C**) 103, and (**D**) 137.

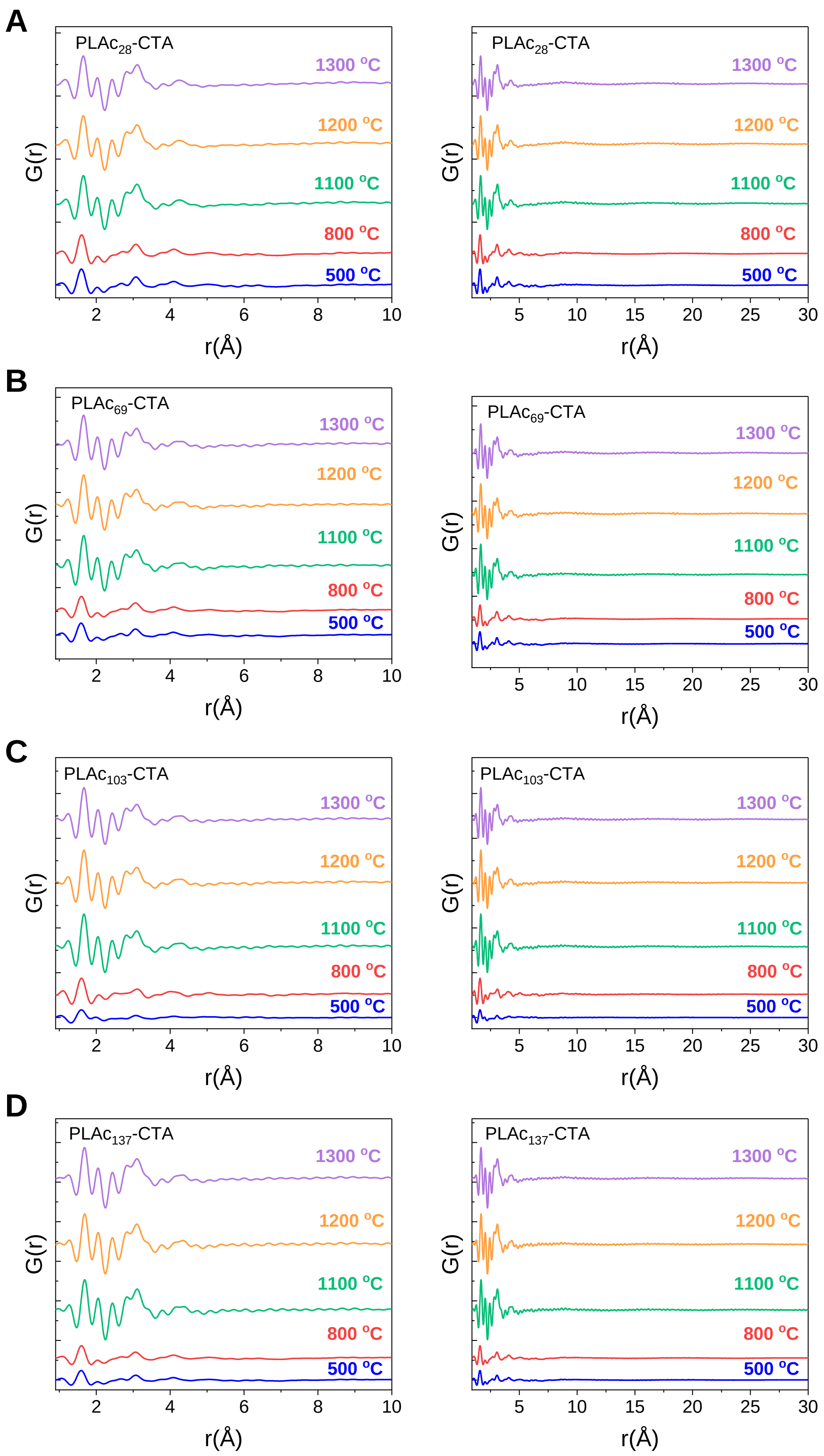

Fig. S24. PDF profiles of the ceramic materials prepared with PLAc-CTA X_n = (**A**) 28, (**B**) 69, (**C**) 137 upon *in situ* heating at different temperatures, shown 10 Å (left side) and 30 Å (right side) distances.

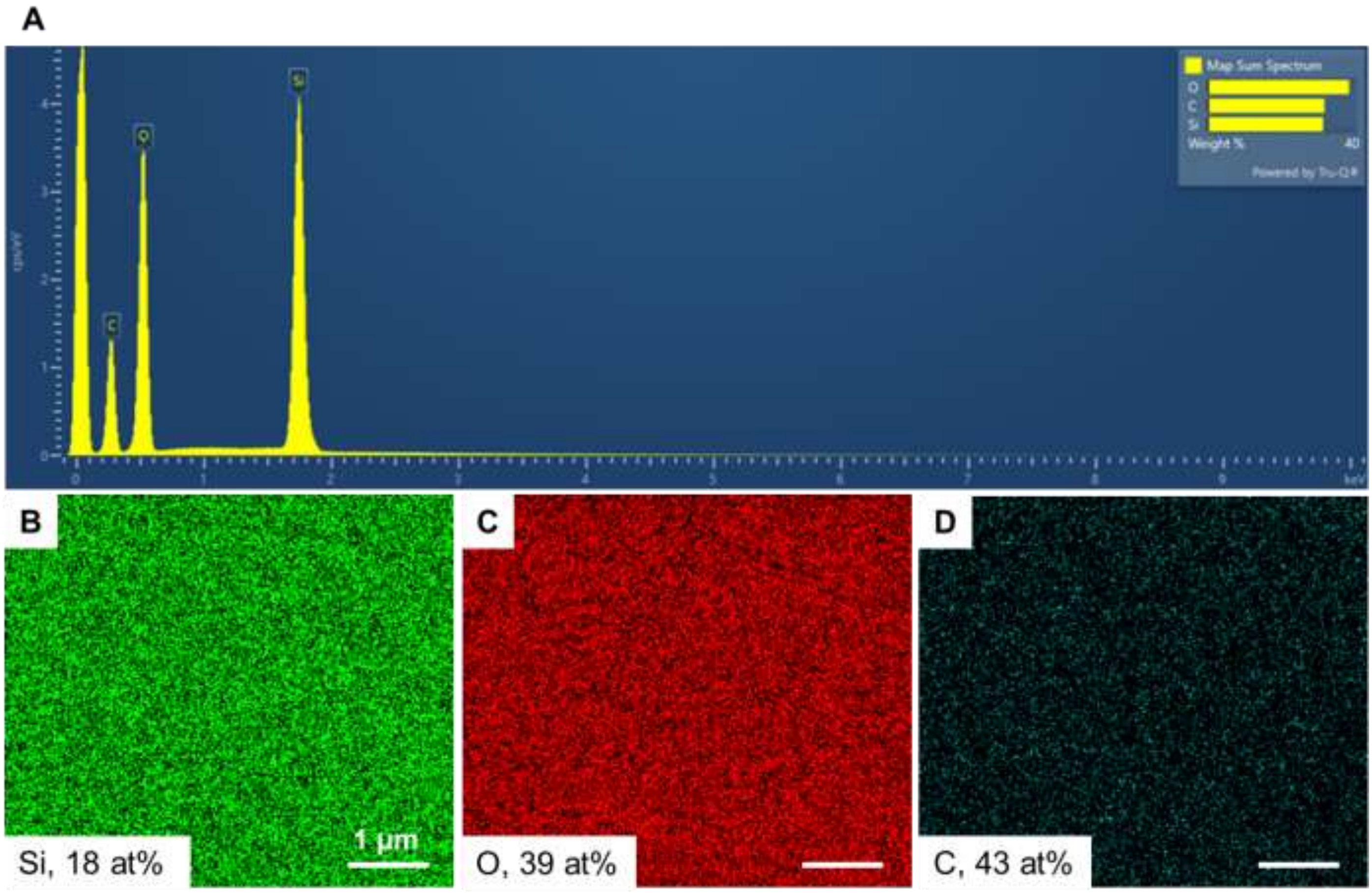

Fig. S25. Energy-dispersive X-ray spectroscopy (EDS) mapping of ceramic materials prepared using pyrolysis at 800 °C. 3D printed preceramic material was prepared using $PLAc_{103}$-CTA. (**A**) EDX spectrum; (**B**) Si; (**C**) O; (**D**) C.

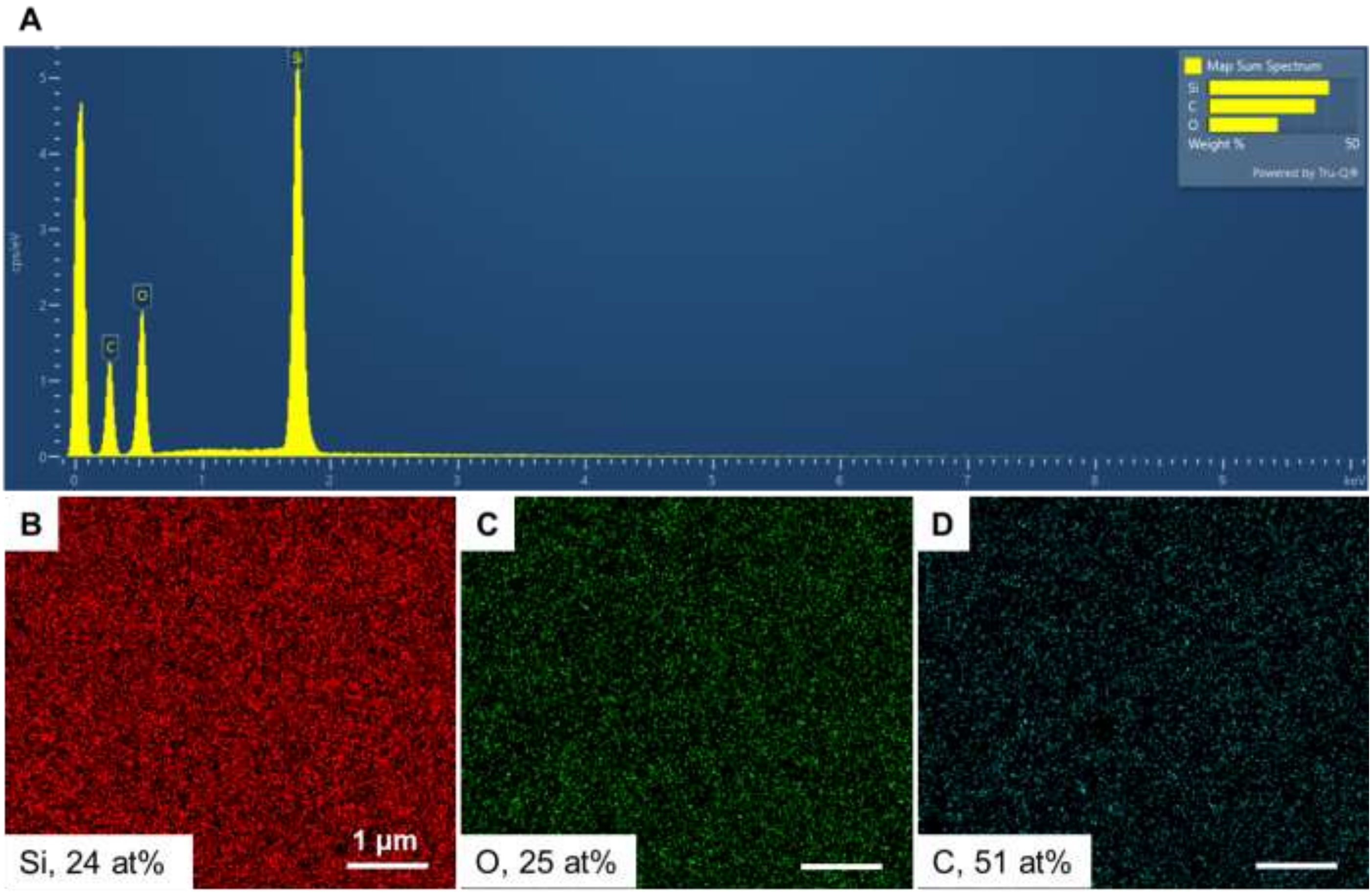

Fig. S26. EDS of ceramic materials prepared using pyrolysis at 1200 °C. 3D printed preceramic material was prepared using $PLAc_{103}$-CTA. (**A**) EDX spectrum; (**B**) Si; (**C**) O; (**D**) C.

***Discussion of EDS results*:** As shown from the EDS spectrum (**Fig. S5A**), the scanned cross-sectional area of the ceramic materials mainly displays the signals of Si, O and C. Elemental mapping revealed uniform distributions of Si, O and C within the analyzed cross-section area with concentrations of 18, 39 and 43 wt%, respectively (**Fig. S25B-D**), suggesting the formation of $SiO_{2.17}C_{2.39}$ ceramic phase. EDS analysis of the ceramic material pyrolyzed at 1200 °C suggested the formation of more silicon- and carbon-rich ceramic phase $SiO_{1.04}C_{2.13}$ compared to the sample pyrolyzed at 800 ºC (**Fig. S26**).

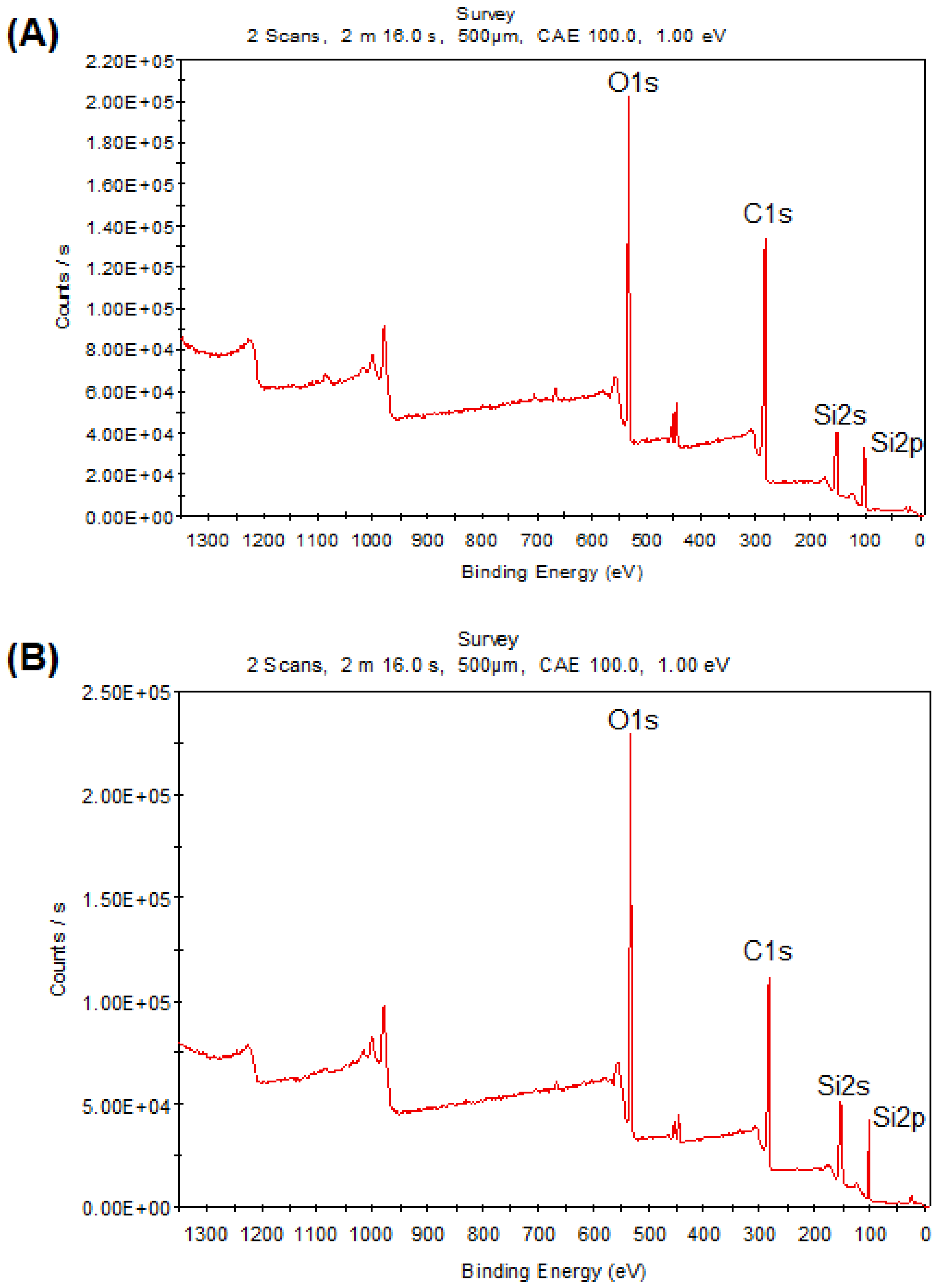

Fig. S27. X-ray photoelectron spectroscopy (XPS) survey spectra for ceramic materials pyrolyzed at (**A**) 800 °C and (**B**) 1200 °C. 3D printed ceramic material was prepared using PLAc$_{103}$-CTA.

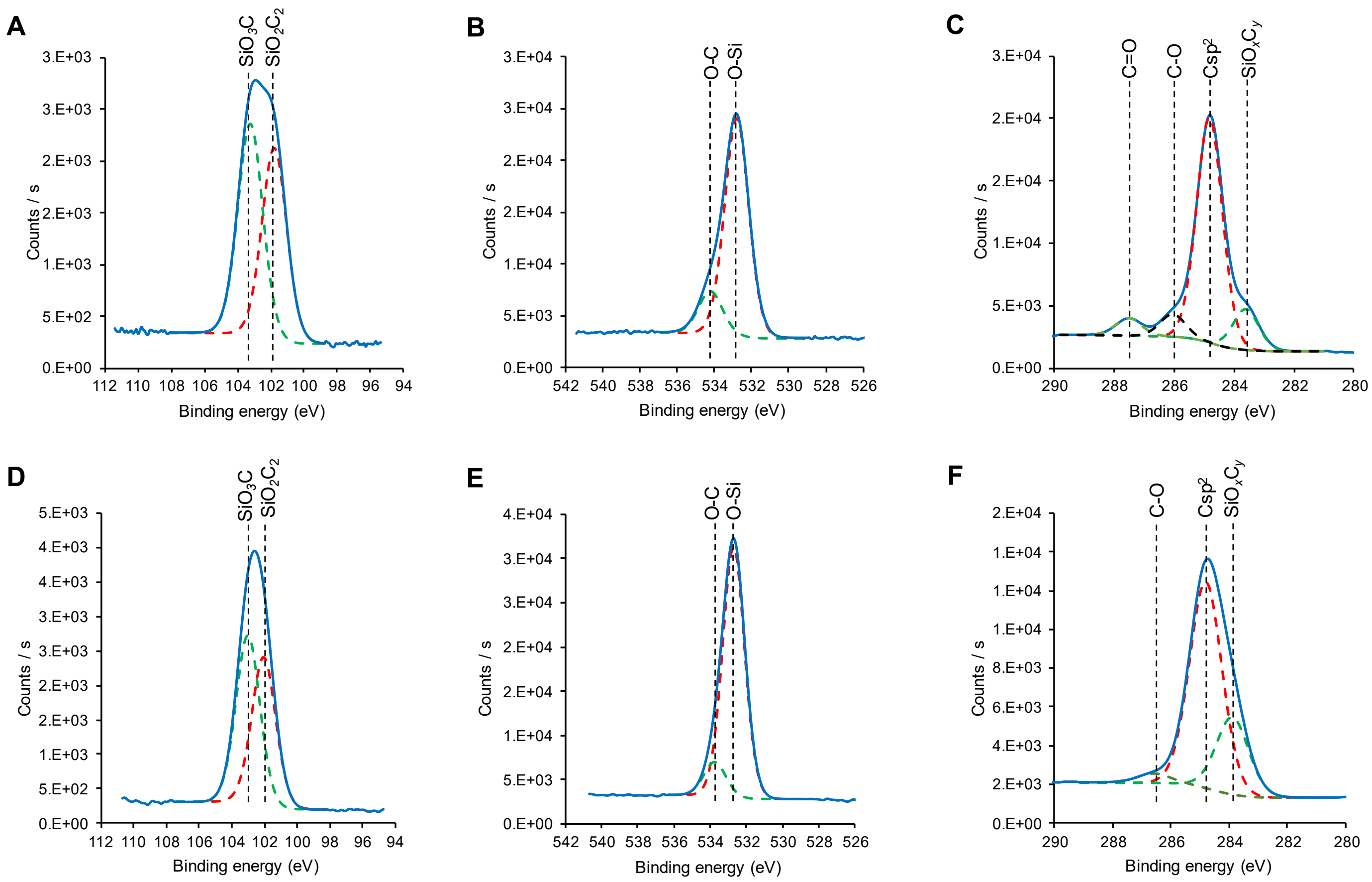

Fig. S28. XPS spectra of ceramics obtained from pyrolysis of 3D printed preceramic PIMS materials at (**A-C**) 800 °C and (**D-F**) 1200 °C. (**A, D**) Si 2p, (**B, E**) O 1s, (**C, F**) C 1s.

Table S7. The compositional analysis, binding energies and the corresponding chemical species of 3D printed nanostructured ceramic materials.

Sample	Peak	Binding energy, eV	Chemical state	Atomic %
3D printed ceramic materials obtained upon pyrolysis at 800 ºC	Si 2p	101.8	SiO_2C_2	7.8
		103.3	SiO_3C	8.6
	O 1s	532.8	O-Si	25.0
		534.2	O-C	4.8
	C 1s	283.6	SiO_xC_y	7.1
		284.8	C sp^2	39.8
		286.0	C-O	4.0
		287.5	C=O	3.0
3D printed ceramic materials obtained upon pyrolysis at 1200 ºC	Si 2p	102.1	SiO_2C_2	9.2
		103.0	SiO_3C	10.3
	O 1s	532.7	O-Si	32.2
		533.7	O-C	4.2
	C 1s	283.9	SiO_xC_y	11.6
		284.8	C sp^2	31.2
		286.6	C-O	1.3

***Discussion of XPS results*:** XPS survey spectra of ceramic materials pyrolyzed at either 800 or 1200 °C were identical and evidenced Si (Si 2s and Si 2p peaks), O (O 1s peak) and C (C 1s peak) bonds (**Fig. S27**). The spectra for Si 2p, O 1s and C 1s were deconvoluted and their respective peaks were assigned according to the literature.[11] The compositional analysis including binding energies (E_b) of identified chemical species are summarized in **Table S7**. For the ceramic materials pyrolyzed at 800 °C, Si exists as the mixture of the two silicon oxycarbide species, i.e., SiO_2C_2 (E_b = 101.8 eV) and SiO_3C (E_b = 103.3 eV) with similar atomic concentrations of 7.8 and 8.6 at% (**Fig. S28A** and **Table S7**). O in the obtained ceramics mainly exists in the form of O-Si species (25 at%, E_b = 532.8 eV) with the presence of low content of O-C form (4.8 at%, E_b = 534.2 eV) (**Fig. S28B**), further suggesting the formation of silicon oxycarbide phase. C largely exists in the form of unsaturated carbon (C sp^2, E_b = 284.8 eV, 39.8 at%), the presence of which can result from the pyrolytic decomposition of organic constituents, e.g., HDODA and LAc, in the crosslinked network of *net*-P(SMP-10-*stat*-HDODA-*stat*-LAc) domains (**Fig. S28C**). Other detected carbon chemical species include SiO_xC_y (E_b = 284.8 eV, 7.1 at%), C-O (E_b = 286.0 eV, 4.0 at%) and C=O (E_b = 287.5 eV, 3.0 at%). The last two chemical species (C-O and C=O) presumably originated from the oxidation of carbon phase. For the ceramic materials pyrolyzed at 1200 °C, the presence of similar chemical species of Si, O and C was observed (**Fig. S28D-F**), although the atomic concentration of C sp^2, C-O and C=O species was lower compared to the material pyrolyzed at 800 °C (**Table S7**). Taken together, the XPS data indicate that the ceramic materials exist in the form of silicon oxycarbide phase (SiO_2C_2 and SiO_3C phases) along with sp^2 C phase.

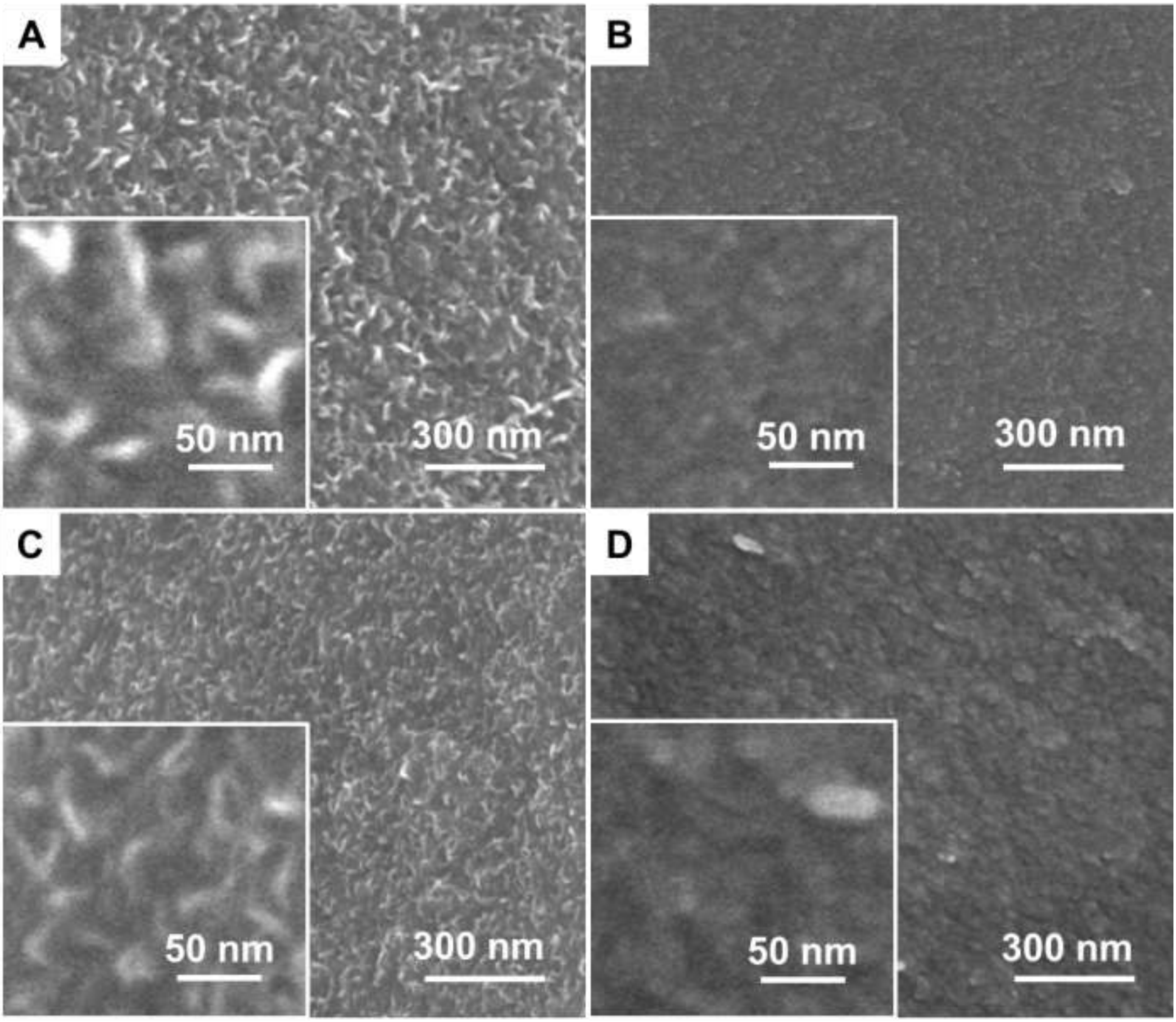

Fig. S29. SEM images (cross-section) of the pyrolyzed preceramic object (1200 °C, under argon) prepared using PLAc-CTA with X_n = (**A**) 28, (**B**) 69, (**C**) 103, and (**D**) 137.

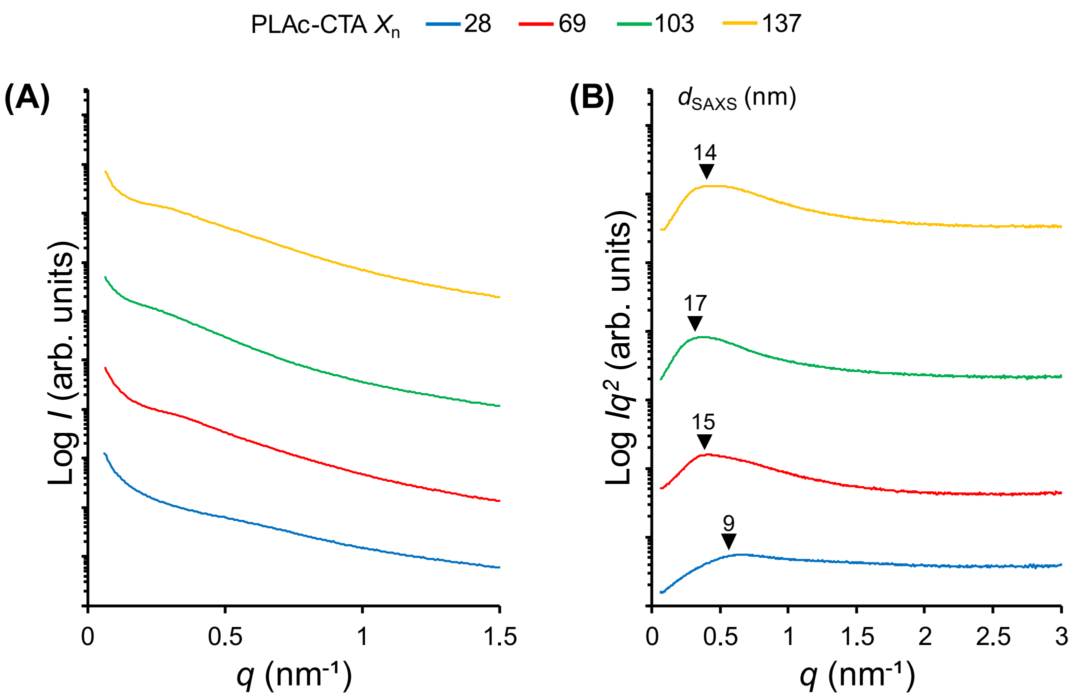

Fig. S30. (**A**) SAXS profiles and (**B**) Lorentz-corrected SAXS profiles and corresponding domain spacing (d_{SAXS}) values of ceramic materials (800 °C, 1 °C min^{-1} under argon) 3D printed using PLAc-CTA with X_n = 28, 69, 103 and 137. SAXS profiles were shifted vertically for clarity.

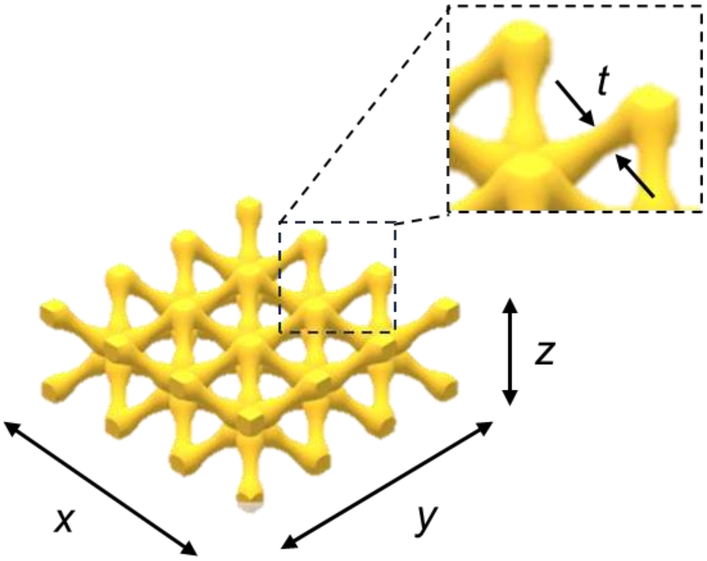

Fig. S31. CAD model of a lattice structure.

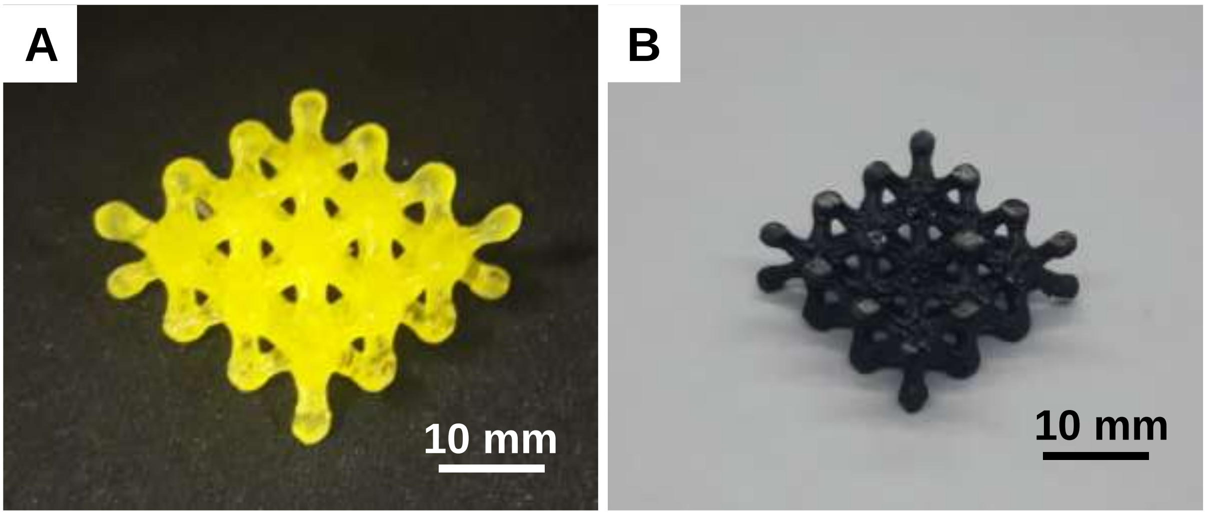

Fig. S32. (**A**) 3D printed PIMS preceramic object. (**B**) Pyrolyzed object (800 °C, 1 °C min^{-1} under argon).

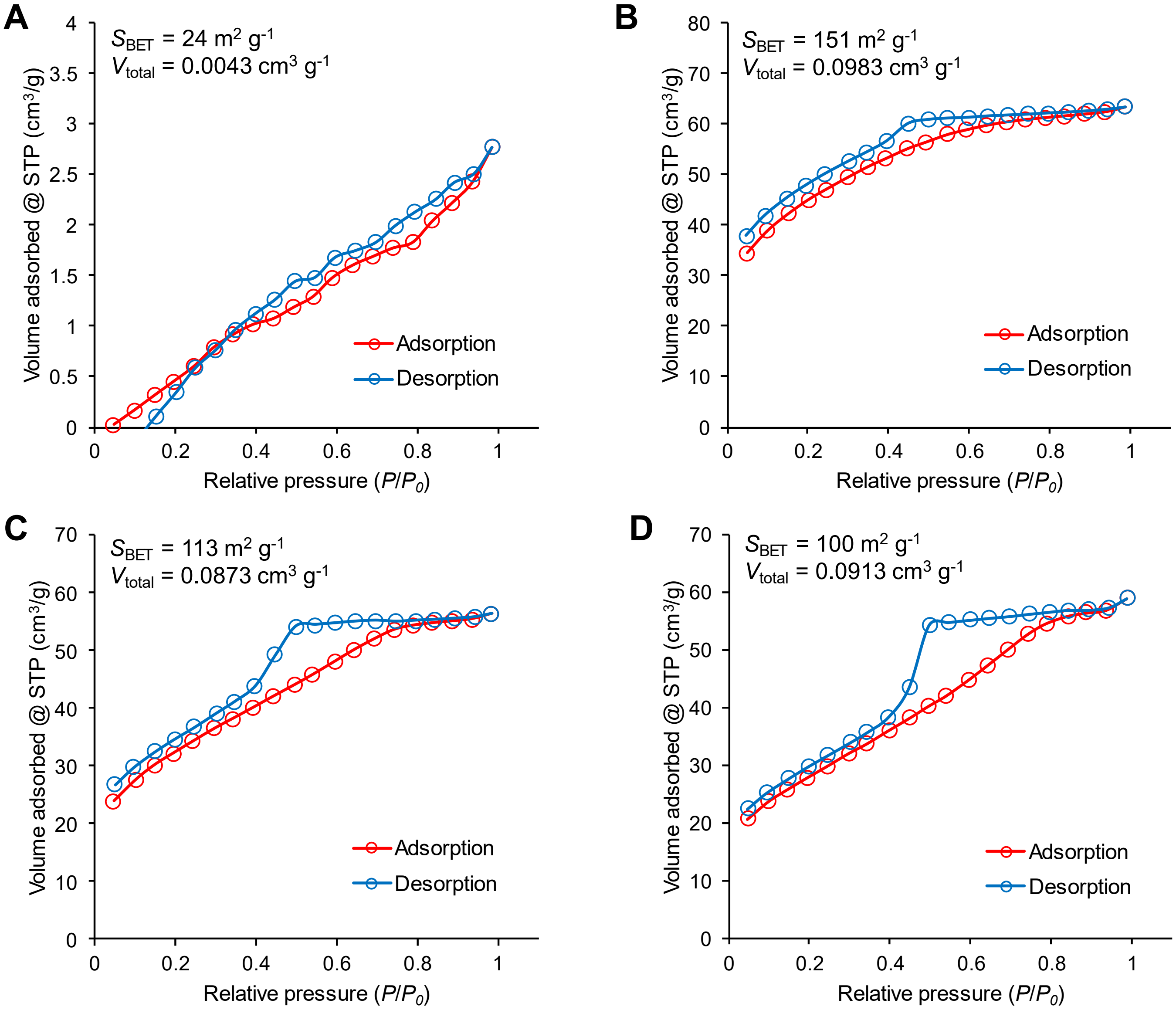

Fig. S33. N_2 at 77 K adsorption – desorption isotherms for ceramic materials prepared using PLAc-CTA with various X_n. X_n = (**A**) 28, (**B**) 69, (**C**) 103, and (**D**) 137. STP, standard temperature and pressure. Pyrolyzed object (1200 °C, 1 °C min^{-1} under argon).

Table S8. The Vickers hardness (HV) values of PIMS ceramic materials.

X_n of PLAc block	Pyrolysis temperature (°C)	Total pore volume (cm^3 g^{-1})	HV (GPa)[a]
28	800	0.11	2.3 ± 0.3
	1200	0.004	11.6 ± 1.4
69	800	0.19	4.4 ± 0.5
	1200	0.10	6.8 ± 2.1
103	800	0.41	3.5 ± 0.3
	1200	0.09	6.7 ± 0.8
137	800	0.20	3.5 ± 0.7
	1200	0.09	8.2 ± 1.0

[a] – Data are shown as mean value ± SD from three independent measurements (*n* = 3).

Note: The Vickers hardness of the 3D printed PIMS ceramic materials increased from 2.3 – 4.4 to 6.8 – 11.6 GPa as the pyrolysis temperature increased from 800 to 1200 °C. This result suggests that the Vickers hardness of ceramics increases as the porosity decreases. Indeed, the ceramic materials obtained at 1200 °C exhibited lower pore volume compared to the materials obtained at 800 °C. This is in agreement with the previous works regarding the Vickers hardness of ceramics.[12] Overall, the measured hardness values for 3D printed PIMS ceramics closely align with the ones reported in the literature for silicon oxycarbide materials.[13] This alignment serves as strong evidence that our 3D printing process successfully fabricates ceramics with mechanical properties comparable to those obtained using conventional approaches.

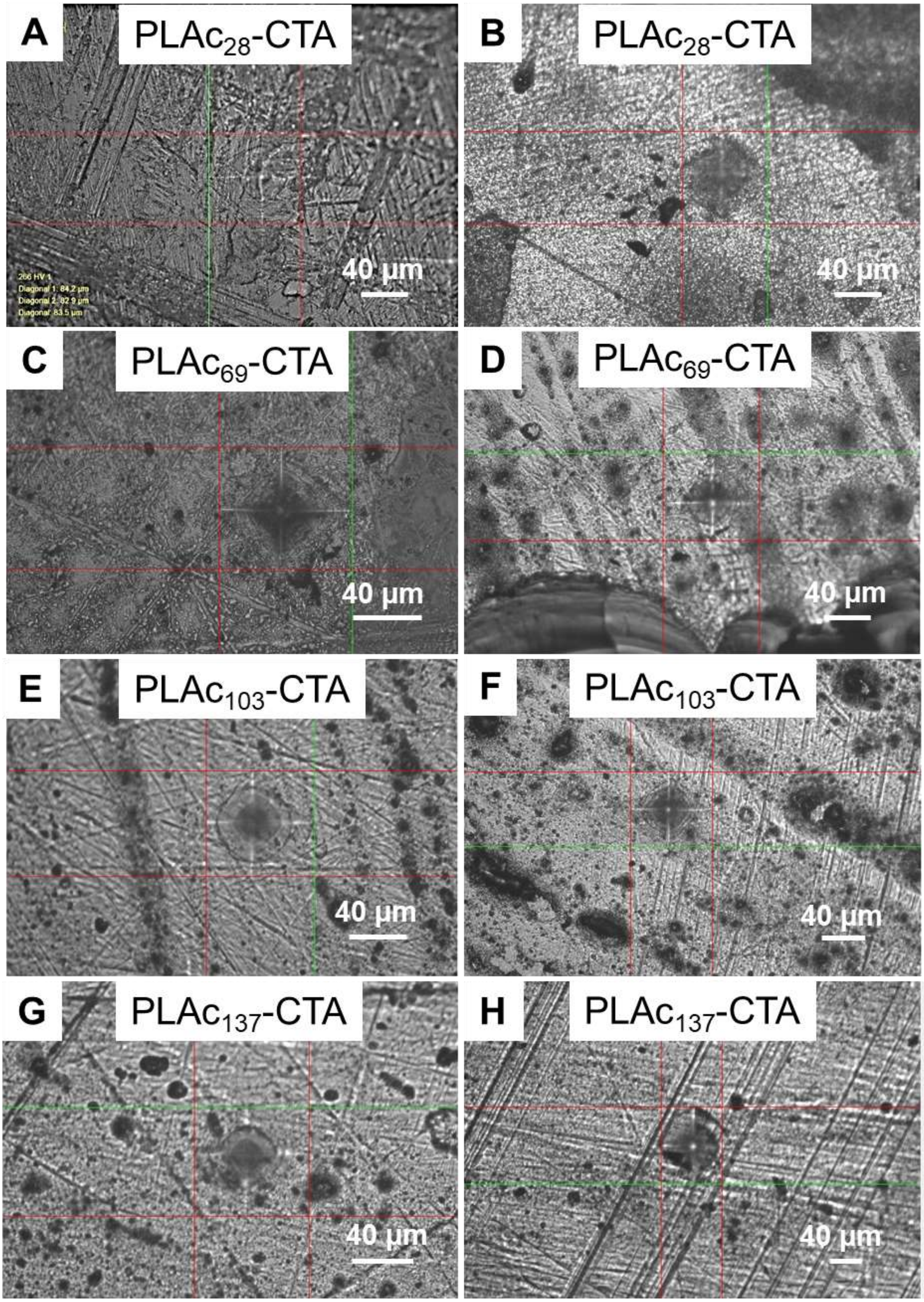

Fig S34. Optical micrographs for the Vickers indentation of the 3D printed PIMS ceramic materials prepared using PLAc-CTA with various X_n and pyrolyzed at (**A, C, E, G**) 800 and (**B, D, F, H**) 1200 °C.

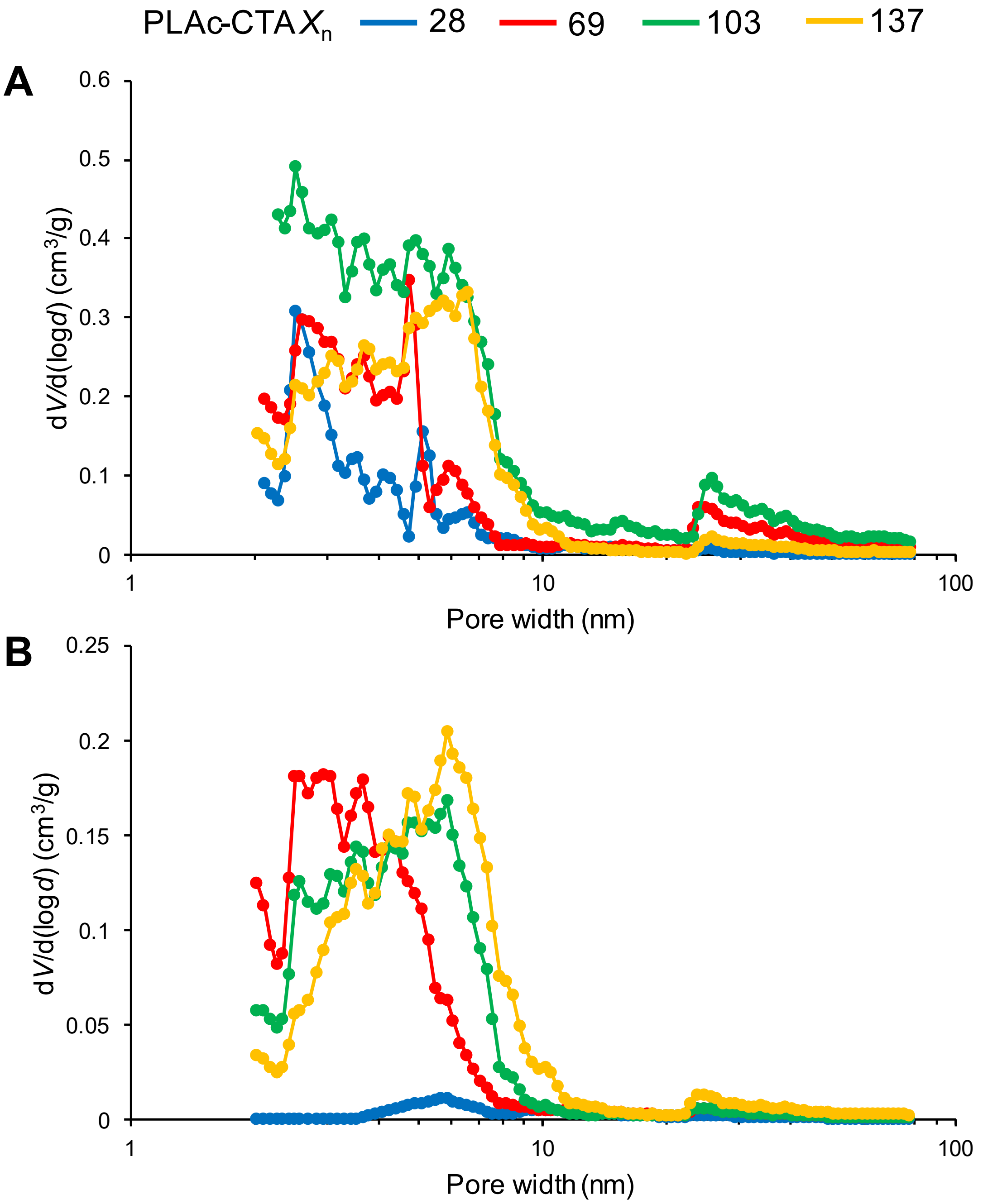

Fig. S35. Calculated pore size distribution based on NLDFT analysis of the adsorbed branch of 3D printed nanostructured ceramic materials prepared using PLAc-CTAs with various X_n. Ceramic materials were pyrolyzed under argon at (**A**) 800 and (**B**) 1200 °C.

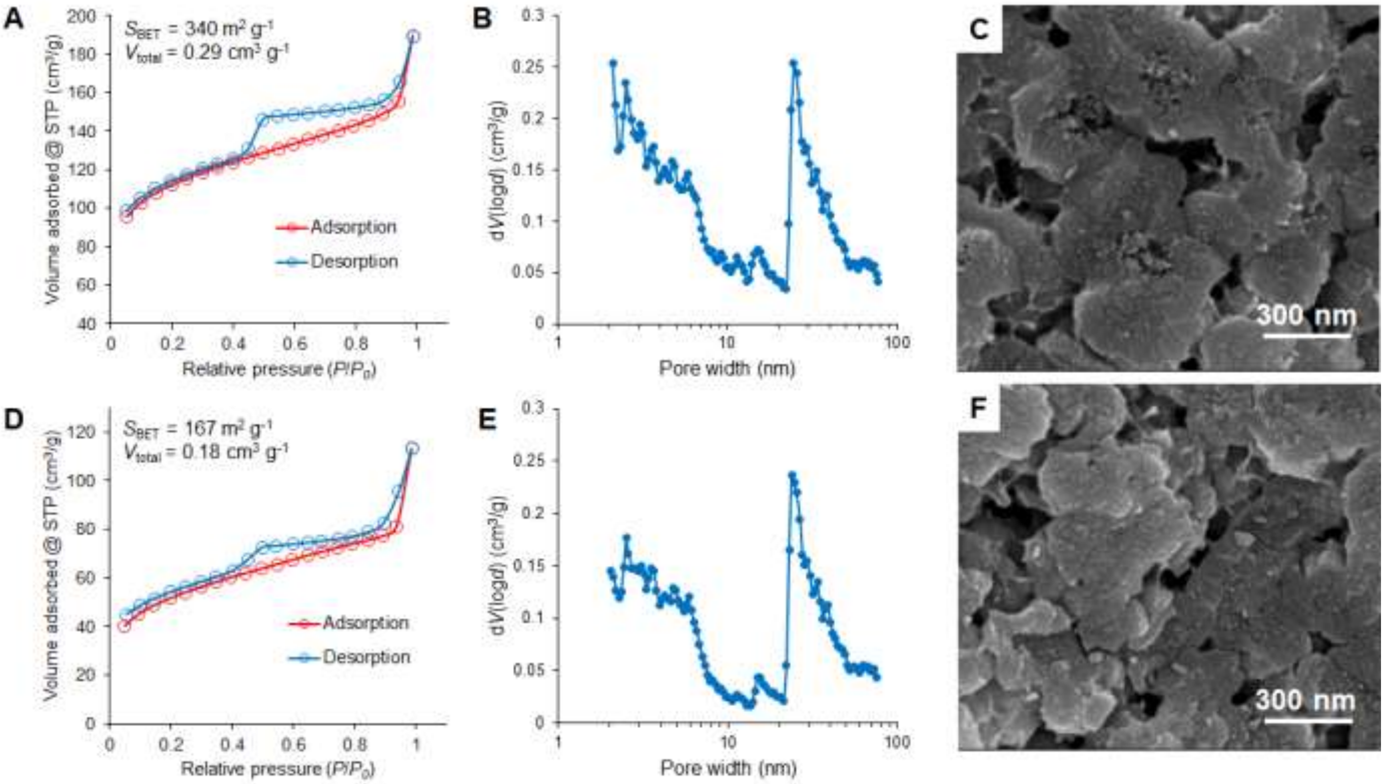

Fig. S36. Characterization of the ceramic materials 3D printed in the absence of PLAc-CTA. Materials 3D printed using the following resin: (**A** – **C**) SMP-10/HDODA/LAc/PLAc$_{103}$/BTPA/BAPO = 36.9/36.9/9.4/14.8/0.1/1.8 (wt%); (**D** – **F**) SMP-10/HDODA/LAc/PLAc$_{103}$/BAPO = 36.9/36.9/9.4/14.9/1.8 (wt%). (**A**, **D**) Nitrogen sorption isotherm; (**B**, **E**) Calculated pore size distribution based on NLDFT analysis of the adsorbed branch; (**C**, **F**) SEM images of the cross-section area.

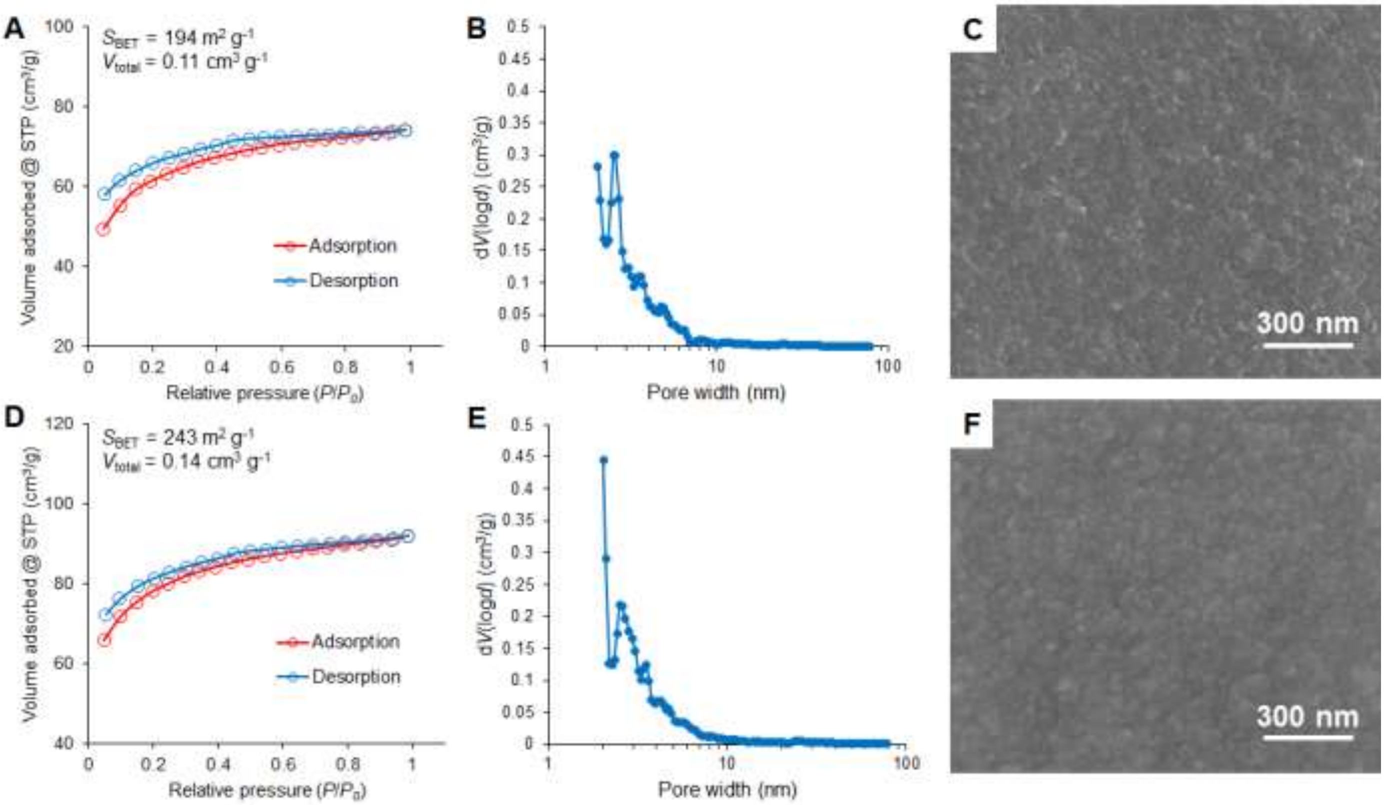

Fig. S37. Characterization of the ceramic materials 3D printed in the absence of PLAc-CTA. Materials 3D printed using the following resin: (**A** – **C**) SMP-10/HDODA/LAc/BTPA/BAPO = 36.9/36.9/24.2/0.1/1.8 (wt%); (**E** – **F**) SMP-10/HDODA/LAc/BTPA/BAPO = 36.9/36.9/24.2/0.2/1.8 (wt%). (**A**, **D**) Nitrogen sorption isotherm; (**B**, **E**) Calculated pore size distribution based on NLDFT analysis of the adsorbed branch; (**C**, **F**) SEM images of the cross-section area.

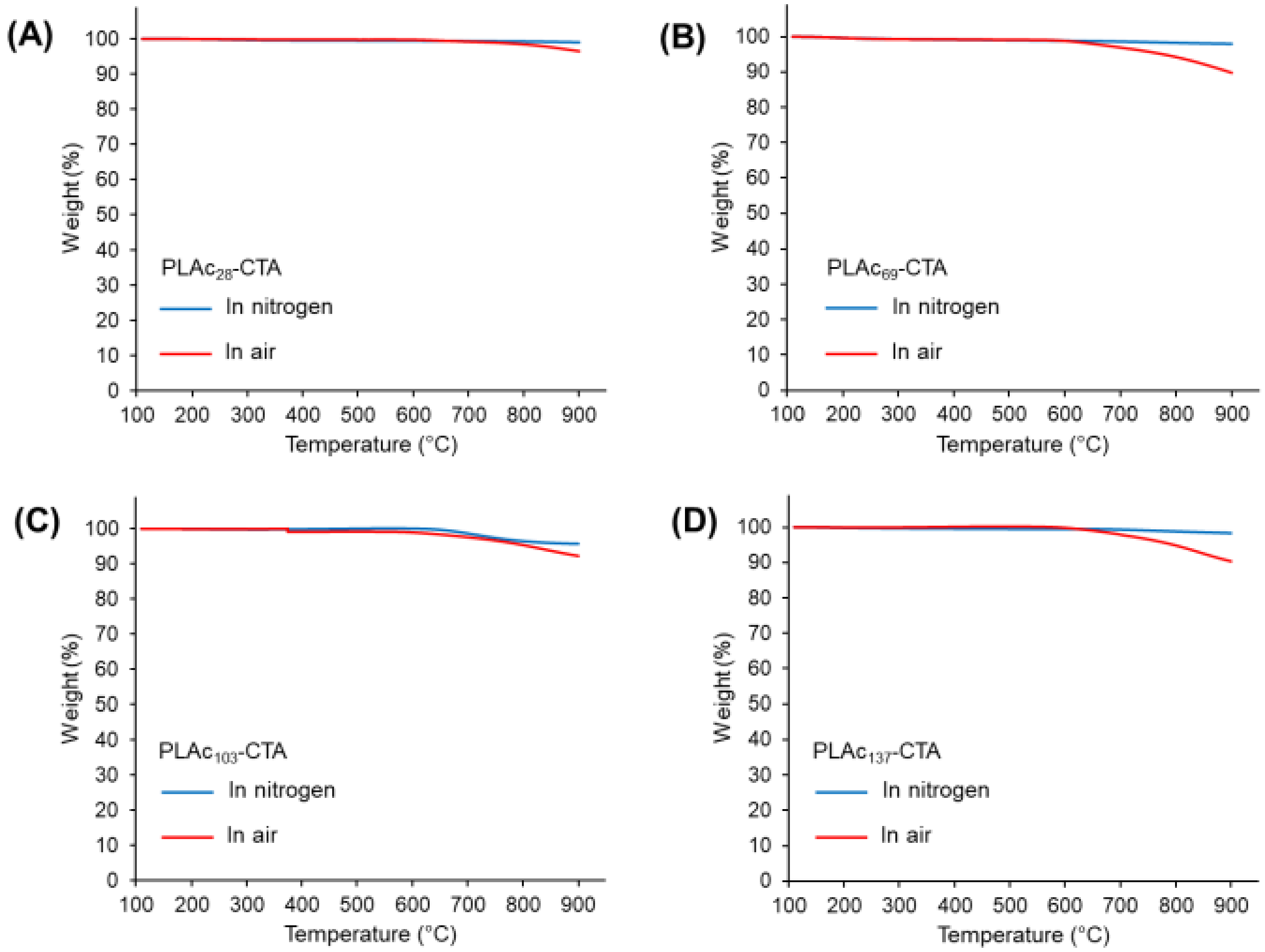

Fig. S38. TGA profiles of ceramic materials derived from the pyrolysis of 3D printed preceramic PIMS materials prepared using PLAc-CTA with X_n = **(A)** 28, **(B)** 69, **(C)** 103, and **(D)** 137. TGA was performed in nitrogen and air. The heating rate was 10 °C min^{-1}.

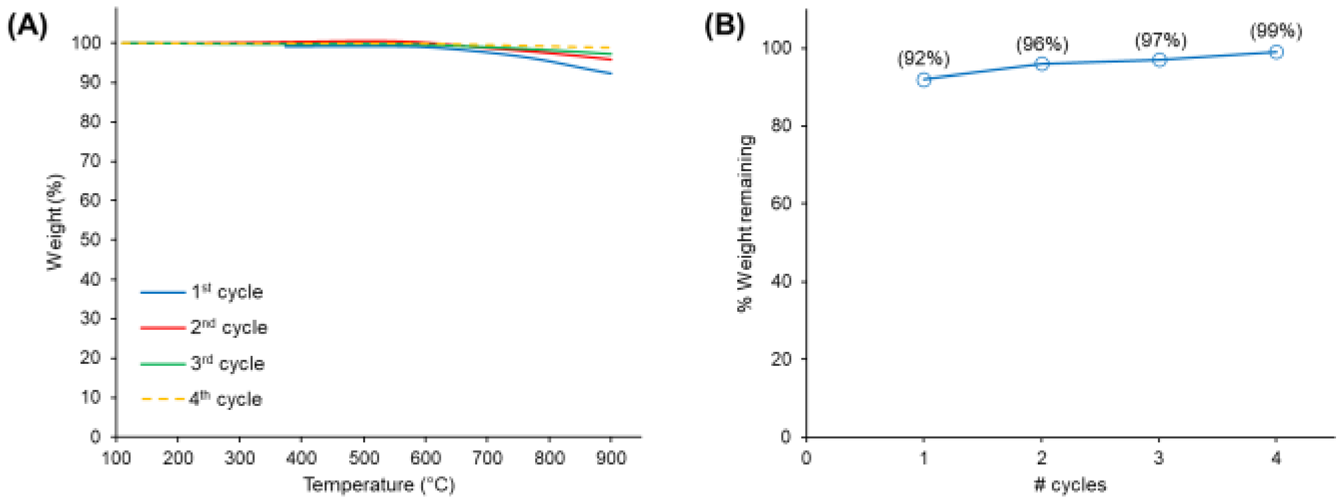

Fig. S39. Thermal stability of ceramic materials derived from the pyrolysis of 3D printed preceramic PIMS materials prepared using $PLAc_{103}$-CTA. **(A)** TGA profiles after 4 cycles of heating in air. The heating rate was 10 °C min^{-1}; **(B)** Remaining mass (%) of ceramics after 4 cycles of heating in air.

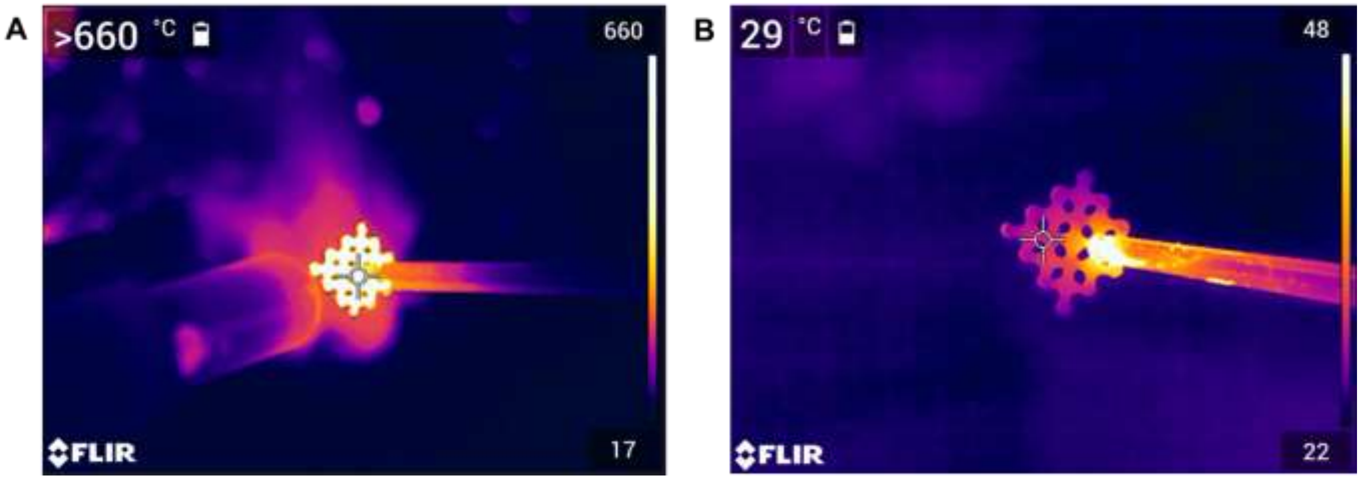

Fig. S40. Infrared thermal images of the 3D printed PIMS ceramic lattice (**A**) during heating in a butane gas flame and (**B**) after heating. PIMS ceramic lattice was prepared using $PLAc_{103}$-CTA and pyrolyzed at 1200 °C under argon (1 °C min^{-1}).

Table S9. Mass and size dimensions of 3D printed PIMS ceramic lattice before and after exposure to a butane gas torch flame. PIMS ceramic lattice was prepared using $PLAc_{103}$-CTA and pyrolyzed at 1200 °C under argon (1 °C min^{-1}).

Exposure to a gas torch flame	Mass (mg)	Size dimensions (mm)[a]			
		x	***y***	***z***	***t***
Before	960	17.93	17.74	6.71	1.2
After	960	17.93	17.74	6.69	1.2

[a] – Size dimensions were determined according to Fig. S31.

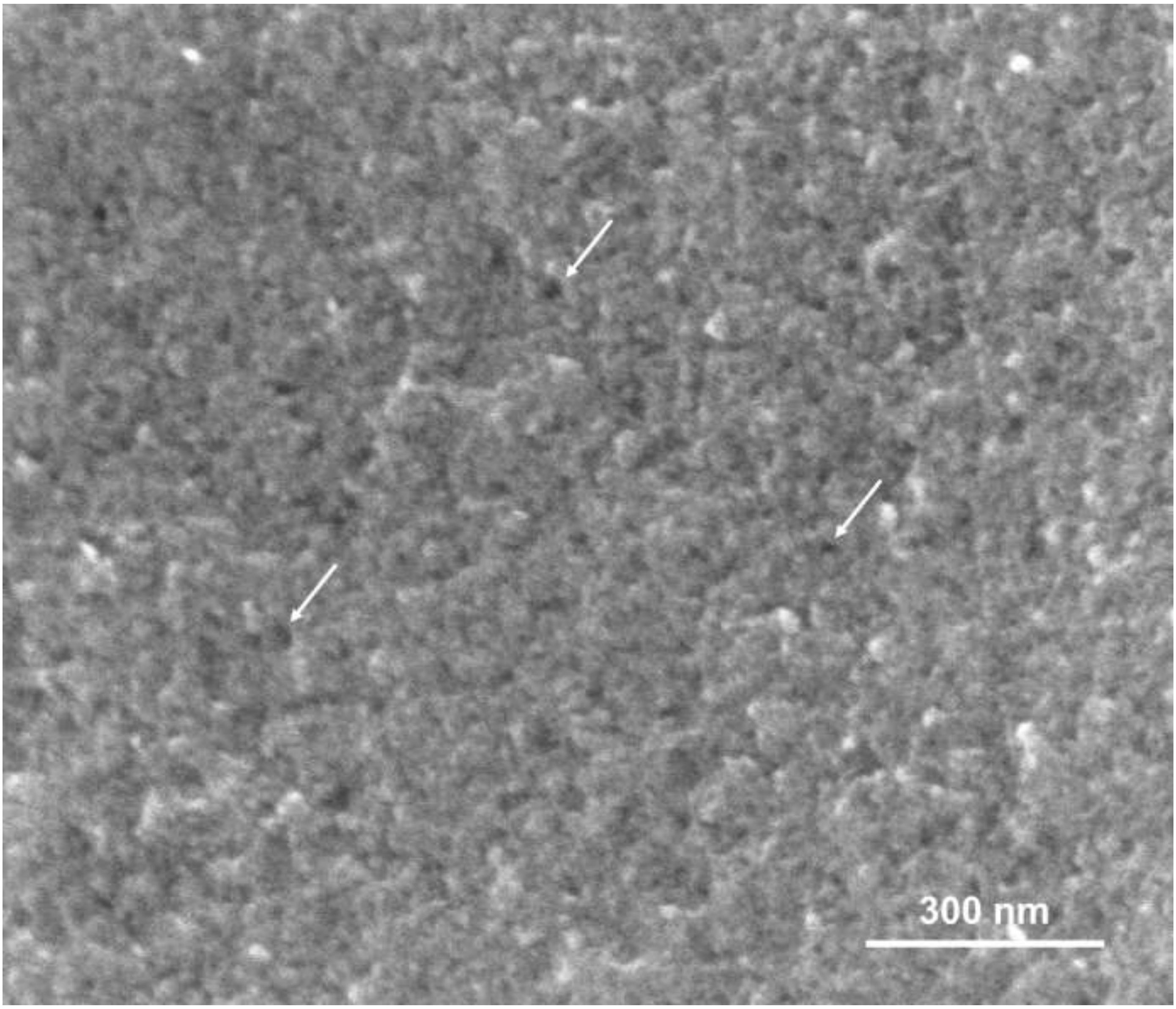

Fig. S41. SEM images (cross-section) of 3D printed PIMS ceramic lattice after exposure to a butane gas torch flame. PIMS ceramic lattice was prepared using $PLAc_{103}$-CTA and pyrolyzed at 1200 °C under argon (1 °C min^{-1}).

Note: After being exposed to a butane gas torch flame with temperature above 1200 °C for 90 s, the 3D printed PIMS ceramic materials maintained nanocoral morphology with the presence of nanoscale voids/pores (indicated by white arrows).

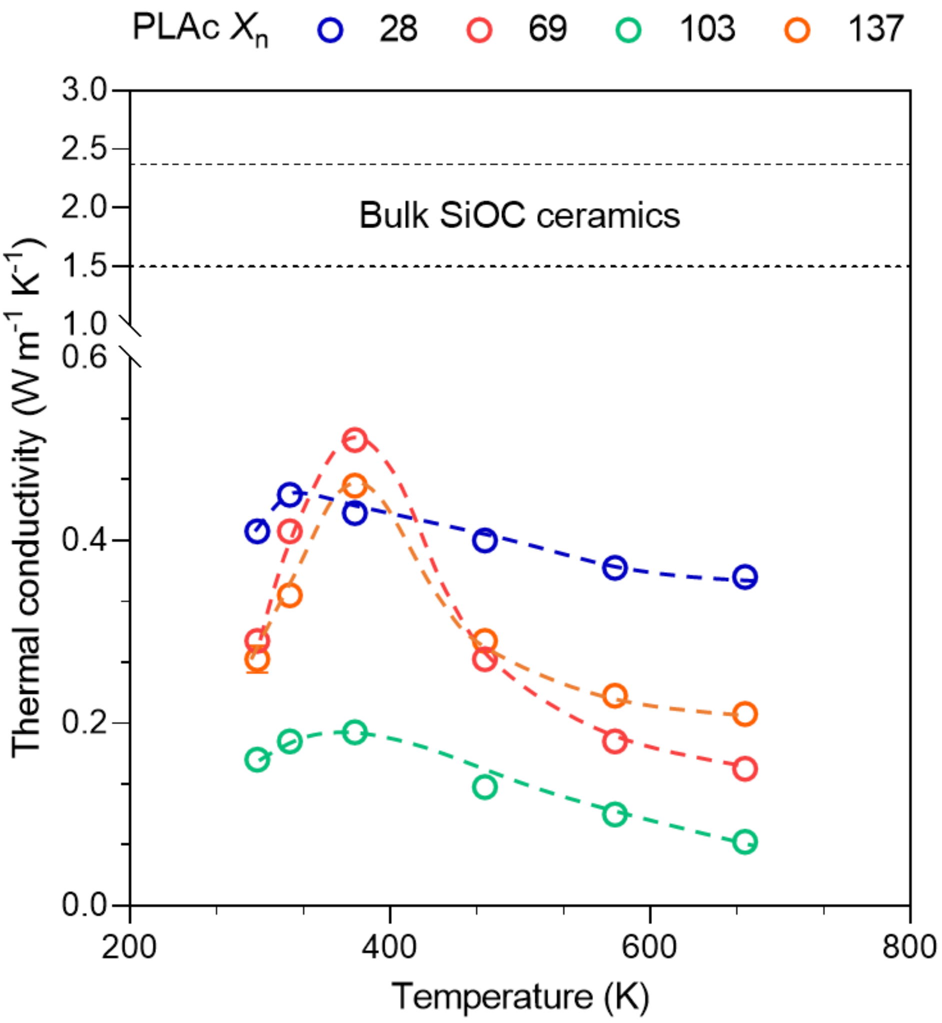

Fig S42. Thermal conductivity values of PIMS SiOC ceramics at different temperature are lower than bulk polymer derived SiOC ceramics (1.5 – 2.4 W m^{-1} K^{-1})[14]. PIMS SiOC ceramics were prepared using PLAc-CTAs with various X_n. Data are shown as mean value ± SD from three independent measurements (n = 3) with dashed line as a guide to the eye. Error bars fall within the size of the markers.

Table S10. Pore characteristics and thermal conductivity values of PIMS ceramic materials.

X_n of PLAc block	Total volume,[a] cm^3	Mass,[b] g	Pore volume (V_{pore}),[c] cm^3	Porosity,[d] %	The degree of pore interconnectivity (η),[e] %	Thermal conductivity at 400 °C (κ), W m^{-1} K^{-1}
28	0.43	0.545	0.06	14	25	0.36 ± 0.004
69	0.47	0.608	0.12	25	43	0.15 ± 0.004
103	0.58	0.588	0.24	42	93	0.07 ± 0.001
137	0.50	0.622	0.12	25	45	0.21 ± 0.003

[a] – Total volume of cylindrical samples was calculated as a volume of cylinder after measuring diameter and thickness of samples using digital caliper. [b] – Mass of samples. [c] – pore volume of samples was calculated by multiplying mass of sample (g) and pore volume (cm^3 g^{-1}, the values are provided in Table 1). [d] – Porosity (%)

was calculated as the ratio of $V_{pore}/V_{total} \times 100\%$. [e] – The degree of pore interconnectivity (η) was estimated as the ratio of $V_{pore}/V_{pore,\ expected}$ according to previously published work.[15] $V_{pore,\ expected}$ was calculated in the Characterization methods section. Note: the pore characteristics of the ceramic materials were determined based on open porosity.

References

[1] M. Teubner, R. Strey, *The Journal of Chemical Physics* **1987**, 87, 3195.

[2] a)E. Gann, C. R. McNeill, M. Szumilo, H. Sirringhaus, M. Sommer, S. Maniam, S. J. Langford, L. Thomsen, *The Journal of Chemical Physics* **2014**, 140, 164710; b)E. Gann, C. R. McNeill, A. Tadich, B. C. C. Cowie, L. Thomsen, *Journal of Synchrotron Radiation* **2016**, 23, 374.

[3] P. J. Chupas, K. W. Chapman, C. Kurtz, J. C. Hanson, P. L. Lee, C. P. Grey, *Journal of Applied Crystallography* **2008**, 41, 822.

[4] a)J. Ilavsky, F. Zhang, R. N. Andrews, I. Kuzmenko, P. R. Jemian, L. E. Levine, A. J. Allen, *Journal of applied crystallography* **2018**, 51, 867; b)J. Bednarcik, J. Gamcova, H. Liermann, *DESY Annual Report* **2012**, 1.

[5] P. Juhas, T. Davis, C. L. Farrow, S. J. L. Billinge, *Journal of Applied Crystallography* **2013**, 46, 560.

[6] a)V. A. Bobrin, Y. Yao, X. Shi, Y. Xiu, J. Zhang, N. Corrigan, C. Boyer, *Nature Communications* **2022**, 13, 3577; b)J. Lee, M. Seo, *ACS Nano* **2021**, 15, 9154.

[7] J. J. Bowen, S. Mooraj, J. A. Goodman, S. Peng, D. P. Street, B. Roman-Manso, E. C. Davidson, K. L. Martin, L. M. Rueschhoff, S. N. Schiffres, W. Chen, J. A. Lewis, M. B. Dickerson, *Materials Today* **2022**, 58, 71.

[8] a)L. Liu, Y. M. Yiu, T. K. Sham, L. Zhang, Y. Zhang, *The Journal of Physical Chemistry C* **2010**, 114, 6966; b)J. Chaboy, A. Barranco, A. Yanguas-Gil, F. Yubero, A. R. González-Elipe, *Physical Review B* **2007**, 75, 075205; c)Y. K. Chang, H. H. Hsieh, W. F. Pong, M. H. Tsai, T. E. Dann, F. Z. Chien, P. K. Tseng, L. C. Chen, S. L. Wei, K. H. Chen, J. J. Wu, Y. F. Chen, *Journal of Applied Physics* **1999**, 86, 5609; d)M. Narisawa, H. Hokazono, K. Mitsuhara, H. Inoue, T. Ohta, *Journal of the Ceramic Society of Japan* **2016**, 124, 1094; e)T. Morresi, M. Timpel, A. Pedrielli, G. Garberoglio, R. Tatti, R. Verucchi, L. Pasquali, N. M. Pugno, M. V. Nardi, S. Taioli, *Nanoscale* **2018**, 10, 13449; f)K. Kanda, S. Suzuki, M. Niibe, T. Hasegawa, T. Suzuki, H. Saitoh, *Coatings* **2020**, 10.

[9] a)Z. Khatami, L. Bleczewski, J. J. Neville, P. Mascher, *ECS Journal of Solid State Science and Technology* **2020**, 9, 083002; b)F. Mangolini, Z. Li, M. A. Marcus, R. Schneider, M. Dienwiebel, *Carbon* **2021**, 173, 557.

[10] L. Espinal, M. L. Green, D. A. Fischer, D. M. DeLongchamp, C. Jaye, J. C. Horn, M. A. Sakwa-Novak, W. Chaikittisilp, N. A. Brunelli, C. W. Jones, *The Journal of Physical Chemistry Letters* **2015**, 6, 148.

[11] a)E. Buet, C. Sauder, S. Poissonnet, P. Brender, R. Gadiou, C. Vix-Guterl, *Journal of the European Ceramic Society* **2012**, 32, 547; b)U. Okoroanyanwu, A. Bhardwaj, V. Einck, A. Ribbe, W. Hu, J. M.

Rodriguez, W. R. Schmidt, J. J. Watkins, *Chemistry of Materials* **2021**, 33, 678; c)K. Shimoda, J.-S. Park, T. Hinoki, A. Kohyama, *Applied Surface Science* **2007**, 253, 9450.

[12] a)G. Zhao, C. Huang, N. He, H. Liu, B. Zou, *International Journal of Refractory Metals and Hard Materials* **2016**, 61, 13; b)Z. Yin, S. Yan, W. Xu, J. Yuan, *Ceramics International* **2018**, 44, 1034; c)J. Song, L. Cao, J. Gao, G. Liang, S. Wang, M. Lv, *Journal of Alloys and Compounds* **2018**, 753, 85.

[13] a)G. D. Sorarù, E. Dallapiccola, G. D'Andrea, *Journal of the American Ceramic Society* **1996**, 79, 2074; b)M. A. Mazo, D. Soriano, J. Rubio, *Ceramics International* **2023**, 49, 12866; c)H.-J. Kleebe, C. Turquat, G. D. Sorarù, *Journal of the American Ceramic Society* **2001**, 84, 1073; d)C. Moysan, R. Riedel, R. Harshe, T. Rouxel, F. Augereau, *Journal of the European Ceramic Society* **2007**, 27, 397.

[14] a)A. Gurlo, E. Ionescu, R. Riedel, D. R. Clarke, *Journal of the American Ceramic Society* **2016**, 99, 281; b)C. Stabler, A. Reitz, P. Stein, B. Albert, R. Riedel, E. Ionescu, *Materials*, 10.3390/ma11020279

[15] Z. Zhou, T. Liu, A. U. Khan, G. Liu, *Science Advances*, 5, eaau6852.
